# The German trial on Aciclovir and Corticosteroids in Herpes-simplex-virus-Encephalitis (GACHE): a multicenter, randomized, double-blind, placebo-controlled trial

**DOI:** 10.1186/s42466-019-0031-3

**Published:** 2019-09-12

**Authors:** U. Meyding-Lamadé, C. Jacobi, F. Martinez-Torres, T. Lenhard, B. Kress, M. Kieser, C. Klose, K. Einhäupl, J. Bösel, M-B Mackert, V. Homberg, C. Koennecke, G. Weißheit, D. Claus, B. Kieseier, J. Bardutzky, T. Neumann-Haefelin, M. W. Lorenz, H. Steinmetz, C. Gerloff, D. Schneider, A. Grau, M. Klein, R. Dziewas, U. Bogdahn, W. Jakob, R. Linker, K. Fuchs, A. Sander, S. Luntz, T. Hoppe-Tichy, D. F. Hanley, R. von Kummer, E. Craemer

**Affiliations:** 1 Department of Neurology, Krankenhaus Nordwest, Frankfurt, Germany; 2 Department of Neurology, University of Heidelberg, Heidelberg, Germany; 3 Department of Neuroradiology, Krankenhaus Nordwest, Frankfurt, Germany; 4 Institute of Medical Biometry and Informatics, University of Heidelberg, Heidelberg, Germany; 5 Department of Neurology, Charité Universitätsmedizin Berlin, Berlin, Germany; 6 Department of Neurology, Vivantes Auguste-Viktoria-Klinikum, Berlin, Germany; 7 Department of Neurology, Vivantes Klinikum im Friedrichshain, Berlin, Germany; 8 Department of Neurology, Klinikum Darmstadt, Darmstadt, Germany; 9 Department of Neurology, Universitätsklinikum Frankfurt, Frankfurt, Germany; 10 Department of Neurology, Universitätsklinikum Hamburg-Eppendorf, Hamburg, Germany; 11 Department of Neurology, Klinikum Kassel, Kassel, Germany; 12 Department of Neurology, Universitätsklinikum Leipzig, Leipzig, Germany; 13 Department of Neurology, Klinikum der Stadt Ludwigshafen am Rhein, Lugwigshafen, Germany; 14 Department of Neurology, Klinikum der Ludwig-Maximilians- Universität München, Großhadern, Germany; 15 Department of Neurology, Universitätsklinikum Regensburg, Regensburg, Germany; 16 Koordinierungszentrum für Klinische Studien (KKS), University of Heidelberg, Heidelberg, Germany; 17 Department of Neurology, Klinikum Fulda, Fulda, Germany; 18 Praxis Dr. Meyer & Prof. Claus, Bensheim, Germany; 19 Department of Neurology, Zentralklinik Bad Berka, Bad Berka, Germany; 20 Department of Pharmacy Heidelberg, Heidelberg University Hospital, Heidelberg, Germany; 21 Department of Neurology, University of Freiburg, Freiburg, Germany; 22 Universitätsklinikum Carl Gustav Carus, Dresden, Germany; 23 Division of Brain Injury Outcomes, John Hopkins University, Baltimore, MD USA

## Abstract

**Introduction:**

Comprehensive treatment of Herpes-simplex-virus-encephalitis (HSVE) remains a major clinical challenge. The current therapy gold standard is aciclovir, a drug that inhibits viral replication. Despite antiviral treatment, mortality remains around 20% and a majority of survivors suffer from severe disability. Experimental research and recent retrospective clinical observations suggest a favourable therapy response to adjuvant dexamethasone. Currently there is no randomized clinical trial evidence, however, to support the routine use of adjuvant corticosteroid treatment in HSVE.

**Methods:**

The German trial of Aciclovir and Corticosteroids in Herpes-simplex-virus-Encephalitis (GACHE) studied the effect of adjuvant dexamethasone versus placebo on top of standard aciclovir treatment in adult patients aged 18 up to 85 years with proven HSVE in German academic centers of Neurology in a randomized and double blind fashion. The trial was open from November 2007 to December 2012. The initially planned sample size was 372 patients with the option to increase to up to 450 patients after the second interim analysis. The primary endpoint was a binary functional outcome after 6 months assessed using the modified Rankin scale (mRS 0–2 vs. 3–6). Secondary endpoints included mortality after 6 and 12 months, functional outcome after 6 months measured with the Glasgow outcome scale (GOS), functional outcome after 12 months measured with mRS and GOS, quality of life as measured with the EuroQol 5D instrument after 6 and 12 months, neuropsychological testing after 6 months, cranial magnetic resonance imaging findings after 6 months, seizures up to day of discharge or at the latest at day 30, and after 6 and 12 months.

**Results:**

The trial was stopped prematurely for slow recruitment after 41 patients had been randomized, 21 of them treated with dexamethasone and 20 with placebo. No difference was observed in the primary endpoint. In the full analysis set (*n* = 19 in each group), 12 patients in each treatment arm achieved a mRS of 0–2. Similarly, we did not observe significant differences in the secondary endpoints (GOS, mRS, quality of life, neuropsychological testing).

**Conclusion:**

GACHE being prematurely terminated demonstrated challenges encountered performing randomized, placebo-controlled trials in rare life threatening neurological diseases. Based upon our trial results the use of adjuvant steroids in addition to antiviral treatment remains experimental and is at the decision of the individual treating physician. Unfortunately, the small number of study participants does not allow firm conclusions.

**Trial registration:**

EudraCT-Nr. 2005–003201-81.

**Electronic supplementary material:**

The online version of this article (10.1186/s42466-019-0031-3) contains supplementary material, which is available to authorized users.

## Introduction

Herpes-simplex-virus-encephalitis (HSVE) is the most common cause of sporadic encephalitis in humans. Worldwide HSVE accounts for 5–10% of all cases of encephalitis. The annual incidence is reported to be between 2 and 4 cases/1,000,000 at any age. Mortality is around 70% and disability is highly prevalent in untreated patients. Poor outcome is more likely with an initial severe course of the encephalitis such as a Glasgow coma scale score (GCS) less than 6 and symptoms already present for more than 4 days [1–3].

Aciclovir is the current standard antiviral therapy for HSVE. In the mid-1980s two large randomized clinical trials showed that the use of intravenous aciclovir reduced the 6-month mortality rate by about 20% and significantly decreased morbidity. However, almost 2/3rds of the survivors display residual neurologic deficits. Even after early antiviral treatment, less than 20% of patients are able to go back to work and the majority of patients suffer from severe disability [1, 2, 4, 5].

Secondary mechanisms of injury beyond the direct virus-mediated tissue damage have been discussed to play a significant role in the pathogenesis of HSVE. On magnetic resonance imaging (MRI) HSVE patients may show late chronic progressive tissue damage despite early treatment with acyclovir [6]. The mechanisms underlying this virus-independent structural damage are believed to be autoimmune in nature [1, 7]. In experimental HSVE, the expression of immunologic Nitric oxid synthase, matrix metalloproteinases and chemokines has been described in the brain and hypothesized to be involved as a secondary mechanism [8–10]. Initial viral load does not correlate with the extent of cranial MRI abnormalities in patients with HSVE [11]. Experimental models of HSVE have shown that viral load is not influenced by treatment with corticosteroids and that the corticosteroids have no adverse effect on the encephalitic process. In a mouse model of HSVE the combination of aciclovir together with steroids has reduced progressive MRI changes compared to aciclovir treatment alone [7, 12, 13].

In humans, steroid treatment of HSVE was used before aciclovir was developed and beneficial effects were described [14, 15]. The only evidence on the adjuvant treatment with steroids in HSVE has been published in case reports or in a retrospective clinical study [2, 16–18]. The non-randomized retrospective study included 45 patients with HSVE. A poorer outcome was present in patients without corticosteroid treatment [17]. Thus far, no prospective or randomized trial evidence is available on the adjuvant treatment with steroids in HSVE.

To further address this question, we designed and initiated a prospective, multicenter, randomized, double-blind, placebo-controlled trial (EudraCT-Nr. 2005–003201-81).

## Methods

### Study design

The German trial of Aciclovir and Corticosteroids in Herpes-simplex-virus-Encephalitis (GACHE) was a prospective, multicenter, randomized, double blind, placebo-controlled, parallel group clinical trial in adult patients with recent onset of HSVE to evaluate the effect of early adjuvant corticosteroids (dexamethasone) on morbidity and mortality in the treatment of adult patients with HSVE [1]. Patients were planned to be recruited in 27 Departments of Neurology of academic medical centers in Germany, Austria and The Netherlands; only centers in Germany actually recruited patients. Recruitment rate was based on the incidence of HSVE (2–4 cases/1,000,000). In addition a survey was conducted in all participating centers estimating the anticipated number of cases. All centers expected between two and five yearly cases of HSVE. Full ethical approval for this study was obtained from all responsible Ethics Committees. An independent data safety monitoring board reviewed the study regularly.

### Study patients

All patients between 18 and 85 years of age with focal neurological signs for not more than 5 days prior to admission to the respective study center were consecutively screened for eligibility and, if considered eligible, invited to participate in the study. Inclusion criteria were age > 18 to < 85 years, laboratory-proven HSVE (detection of HSV-DNA in the cerebrospinal fluid (CSF) by polymerase chain reaction (PCR) or a positive CSF HSV-specific antibody-index) and focal neurological signs for not more than 5 days prior to admission. For women of childbearing potential a negative pregnancy testing in urine was necessary [1]. Exclusion criteria are listed in Table 1. Written informed consent was obtained from each subject or from the subject’s legal representative or designee before any protocol-specific procedures were performed.

**Table 1:** Exclusion criteria

• History of hypersensitivity to corticosteroids	
• Systemic corticosteroid treatment within the last six months or at present time (> 20 mg p.o. or generally intravenous intake)	
• Two fixed dilated pupils	
• Pre-event score mRS more than two or Barthel Index less than 95	
• Pregnancy	
• Breast feeding women	
• Recent history of active tuberculosis or systemic fungal infection	
• Recent head trauma/neurosurgery/peptic ulcer disease	
• Life expectancy less than three years	
• Other serious illness that confound treatment assessment	
• Simultaneous participation in another clinical trial	
• Previous participation in another clinical trial in the last 30 days	
• Previous participation in this clinical trial	
• Women of childbearing potential who are not using a highly effective birth control method	
• Acute viral infections other than HSVE (herpes zoster, poliomyelitis, chickenpox)	
• Hepatitis B surface Antigen-positive chronic active hepatitis	
• Approximately eight weeks before to two weeks after prophylactic vaccination	
• Lymphadenitis following Bacille Calmette Guérin vaccination.	

### Randomization and blinding

Study treatment allocation was done in a 1:1 ratio by the method of minimization considering two factors, study center and GCS (dichotomized: ≤8, > 8). Randomization was performed using the web-based software randomizer (http://www.randomizer.at). The trial was conducted in double-blind fashion with the patient, the treating physician, the observer and all other site personnel as well as the monitor being unaware of the treatment assignment. Dexamethasone and placebo were provided in identically appearing vials and study kits.

### Treatment arms

All patients were treated with aciclovir intravenously at a dosage of 10 mg/kg body weight every 8 hours with an infusion time of 1 h, if patients had a normal renal function. In case of reduced creatinine clearance (< 60 ml/min) the aciclovir dosage was adapted. In addition, they received the study medication, either intravenous Dexamethasone at a dosage of 40 mg every 24 h for 4 days, or placebo that was identical in appearance to the active drug.

All concomitant medications were allowed with exception of actual or long-standing corticosteroid treatment. Gastric protection with an antacid medication was obligatory during the administration of the study medication.

### Endpoints

Functional outcome at 6 months (±14 days) after randomization was assessed by the modified Rankin scale (mRS), a seven-point-scale (0 to 6 points) with 0 indicating normal neurological function, 6 indicating death and the points in between different grades of disability and dependence. This scale is frequently used in ischemic stroke outcome trials.

The primary endpoint was the mRS dichotomized at 0–2 vs 3–6, with 0–2 regarded as favorable outcome without dependency. A mRS of 3 to 6 was interpreted as unfavorable outcome.

Secondary endpoints included mortality after 6 and 12 months, functional outcome after 6 months measured with the Glasgow outcome scale (GOS), functional outcome after 12 months measured with mRS and GOS, quality of life as measured with the EuroQol 5D instrument (EQ-5D) after 6 and 12 months, neuropsychological testing including Mini-Mental test (MMT), Rey Complex Figure Test, Auditory Verbal Learning and Memory Test (AVLT I and II), Digit Test, Trail-Making Test, Word Fluency Test, Hospital Anxiety and Depression Scale (HADS) after 6 months, cranial MRI findings after 6 months, seizures up to day of discharge or at the latest at day 30, and after 6 and 12 months.

### Statistical analysis

The statistical design was that of a group sequential design with a maximum of three stages, rejection boundaries according to O’Brien and Fleming [19] and a potential sample size adjustment for the last stage [20]. Initial assumptions for sample size calculation were a failure rate of 40% in the aciclovir monotherapy group and a failure rate of 25% for the combination therapy of aciclovir plus corticosteroids. For a specified type I error rate of 5% and equally spaced stages this treatment difference can be detected with a power 80% if a maximum of 52 patients per group and stage are included, i.e. the maximum total sample size is 312 patients (ADDPLAN; Release 3). Assuming a rate of loss to follow-up of 15% it was planned to include 62 patients per group in each stage which results in a maximum of 372 patients for the entire study. In the second interim analysis a sample size recalculation based on the pooled data of the first and second stage was planned with the option to adapt the sample size to a maximum of 450 patients for the whole study. For more details see [1].

Due to slow recruitment, the study was terminated prematurely before the first interim analysis. Therefore, the final analysis was done according to a fixed sample size design and all results will be interpreted only descriptively.

The primary analysis is based on the full analysis set (FAS) following the intention-to-treat principle. It comprises all patients treated with at least one dose of study medication (aciclovir and dexamethasone/placebo) and analyzed in the group randomized to. As sensitivity analysis, the primary endpoint was additionally analyzed as per protocol (PP). Patients were included into this PP analysis only if all inclusion and exclusion criteria were fulfilled and all medical procedures/visits have been carried out according to the protocol (dexamethasone/placebo over 4 days, if not dead: 6-month visit within ±14 days). In case of randomization through randomization list without entering into the randomization tool afterwards, the patient was excluded from the PP set. The affiliation to the PP set in disputable cases was fixed prior to database closure in a separate document.

For the FAS analysis, missing values for the primary endpoint were imputed using the last observation-carried-forward approach. Patients who died between randomization and the 6 months visit were evaluated with mRS = 6 at the 6 months visit. In addition, worst case and best case scenarios were evaluated based on the FAS meaning that in both.

treatment groups the worst (mRS > 2) or best (mRS ≤ 2) outcome was assumed in case of missing values.

Patients who died between randomization and the 12 months visit were evaluated with mRS = 6 at the 12 months visit. HADS defines two subscales, the anxiety score and the depression score. Each score was calculated adding up the values given for seven items. In case of one missing value within a subscale, this value was imputed using the mean value of the six other items. If more than one item was missing within a subscale, no imputation was done. The subscales were categorized into negative (0–7), neutral (8–10) and positive (≥11) and tabulated by treatment. For further secondary endpoints no imputation was done. For the PP set no missing values were imputed.

All secondary endpoints were analyzed based on the FAS and the PP set. In case of categorical variables, *p*-values correspond to the Chi^2^-test, in case of continuous variables, p-values are given for the nonparametric U-test. Mortality was evaluated through Kaplan Meier curves and log rank tests were performed.

Due to the small number of randomized patients and the partly high amount of missing data (e.g. for GCS Scale at baseline) no further analyses such as regression analyses were conducted.

The main safety parameter is death which was analyzed as secondary endpoint. Additionally to that, death was analyzed in the same manner based on the safety analysis set which contains all patients who were treated at least for 1 day (aciclovir dexamethasone/placebo) and patients were categorized into groups as treated.

All analyses have been done using validated SAS® Version 9.1 run on a Linux system. Stacked bar graphs for mRS have been created using R Version 3.5.1.

## Results

### Demographics and baseline characteristics

**Fig. 1:**
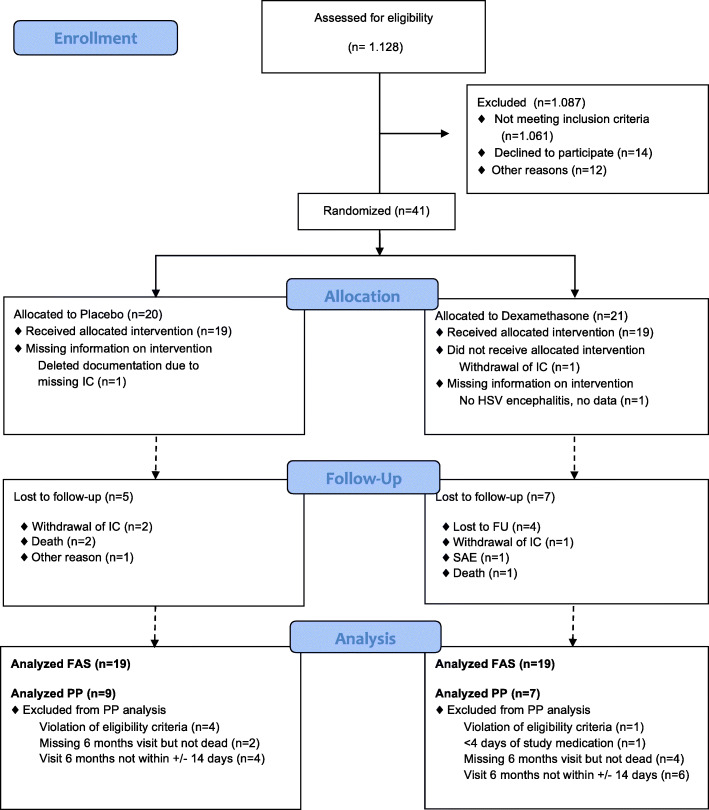
Flow chart of enrolled patients. Shows the flow chart or patient enrollment. We have screened 1.128 Patients, after exclusion of 1.087 patients, finally 41 patients were enrolled in the trial.

One thousand one hundred twenty-nine patients were screened by 27 centers between November 2007 and December 2012. After exclusion of 1087 patients, finally 41 patients were randomized to dexamethasone plus aciclovir (*N* = 21) (dexamethasone group) or to placebo plus aciclovir) (*N* = 20) (placebo group) (Fig. 1). A CONSORT flow chart is presented in Fig. 1. We had to exclude 3 patients from FAS after randomization because of informed consent withdrawal (*N* = 2) and screening failure (*N* = 1). Both treatment groups were characterized using descriptive methods based on the FAS and PP set. In this paper we describe the FAS group, for further details of the PP set we refer to the online published data set (see electronical Additional file 1). Table 2 summarizes the demographics for the patients in the FAS which consist of 19 patients in each group. No severe group differences were present at baseline. Patients in the Dexamethasone group were on average 3 years older and had more often neurological signs at onset (78.9% vs. 63.2%). The pre-encephalitis mRS was also minimally higher in the Dexamethasone group (mRS 0: Placebo *n* = 17, Dexamethasone *n* = 14; mRS ≥1: Placebo *n* = 2, Dexamethasone *n* = 5).

**Table 2:** Baseline characteristic of the patients in the FAS

	Placebo	Dexamethasone	Total
	*N* = 19	*N* = 19	*N* = 38
Gender			
male	10 (52.6%)	11 (57.9%)	21 (55.3%)
female	9 (47.4%)	8 (42.1%)	17 (44.7%)
Age			
Mean +/− SD	58.6 +/− 15.0	61.6 +/− 12.1	60.1 +/− 13.6
Focal neurological signs			
yes	12 (63.2%)	15 (78.9%)	27 (71.1%)
Seizures within last 5 days			
yes	11 (57.9)	11 (57.9%)	22 (57.9%)
Result of PCR			
positive	19 (100.0%)	19 (100.0%)	38 (100.0%)

### Primary endpoint

**Fig. 2:**
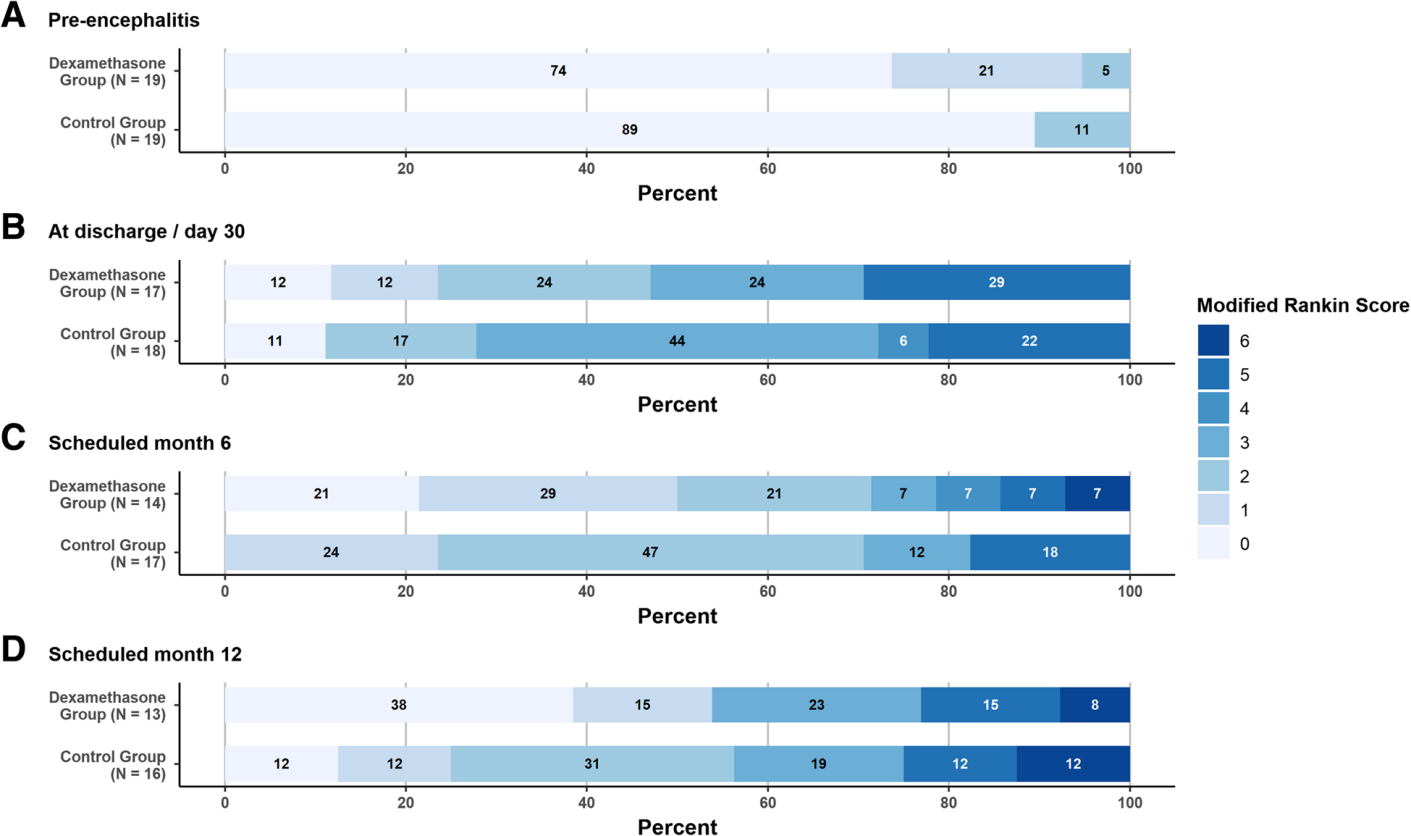
Functional outcome (mRS), Stacked bar plots mRS. Shows the functional outcome as stacked bar plots at Day 30, after 6 and 12 months in percentage.

No differences in the dichotomized mRS outcome scale were found between the Placebo and Dexamethasone groups. In both groups, 12 patients had a mRS ≤2, seven patients had > 2 (*p* = 1.0), respectively. This was confirmed in the sensitivity analyses. Table 3 and Fig. 2 show the mRS at 6-month visit, for both study groups, placebo and dexamethasone.

**Table 3:** Results for the FAS after 6 and 12 months (mRS, mortality)

	Placebo	Dexamethasone	Total	*p*-value
	*N* = 19	*N* = 19	*N* = 38	
Dichotomized mRS at 6 months				
< = 2	12 (63.2%)	12 (63.2%)	24 (63.2%)	1.0000
> 2	7 (36.8%)	7 (36.8%)	14 (36.8%)	
Mortality at 6 months				
Alive	18 (100%)	14 (93.3%)	32 (97.0%)	0.2660
Dead	0 (0)%)	1 (6.7%)	1 (3.1%)	
Missing	1	4	5	
Dichotomized mRS at 12 months				
< = 2	9 (56.3%)	7 (53,8%)	16 (55.2%)	0.8970
> 2	7 (43.8%)	6 (46.2%)	13 (44.8%)	
Missing	3	6	9	
Mortality at 12 months				
Alive	13 (86.7%)	11 (91.7%)	24 (88.9%)	0.6812
Dead	2 (13.3%)	1 (8.3%)	3 (11.1%)	
Missing	4	7	11

### Secondary endpoints

Results for secondary endpoints are given in Tables 3 and 4. Overall, 3 patients died within 12 months. In the dexamethasone group, one patient died within 6 months. In the placebo group 2 patients died within 12 months. No differences in the mortality rates could be detected (Table 3). Neither after six (*p* = 0.266), nor after 12 months (*p* = 0.681). Similarly, no relevant differences in the functional outcome after six and 12 months were found. The GOS after 6 months (*p* = 0.46) and 12 months (*p* = 0.773) turned out to be similar in both groups. Furthermore, the quality of life was not different between the groups after 6 months [EQ-5D-Index (*p* = 0.243) and EQ-5D-VAS (*p* = 0.184)] and 12 months [EQ-5D-Index (*p* = 0.812) and EQ-5D-VAS (*p* = 0.296)] as well as the modified ranking scale after 12 months (*p* = 0.897). No relevant differences were found in all neuropsychological tests after 6 months (see Table 4). Seizure activity was similar in both groups up to day of discharge or at the latest at day 30 (*p* = 0.632), and after 6 months (*p* = 0.713) and 12 months (*p* = 0.271) as well.

**Table 4:** Results for the FAS after 6 months (neuropsychology)

	Placebo	Dexamethasone	Total	*p*-value
	Mean +/− SD	Mean +/− SD	Mean +/− SD	
MMT (total score)	26,7 +/− 2,6	26,7 +/− 2,6	26,6 +/−3,1	0,775
Rey Complex Figure Test (sum of copy/immediate/recall)	43,8 +/− 11,3	41,1 +/− 9,9	42,6 +/−10,5	0,602
AVLT I and II				
Learning Trial	42,1 +/− 16,9	30,6 +/− 12,3	42,6 +/−10,5	0,103
Interference trial	7,4 +/− 5,6	4,2 +/− 4,2	5,8 +/−5,1	0,163
Delayed recall	6,1 +/− 4,7	3,8 +/− 4,3	5,0 +/−4,5	0,244
Digit Span				
forward Total	5,8 +/− 2,4	8,0 +/− 2,3	6,9 +/−2,5	0,051
backward Total	5,2 +/− 2,0	5,6 +/− 1,9	5,4 +/−1,9	0,585
Trail Making Test				
Time Test A (sec.)	45,5 +/− 45,3	60,0 +/− 40,4	52,7 +/−42,7	0,101
Time Test B (sec.)	155,3 +/− 198,4	160,0 +/− 95,0	157,8 +/− 150,2	0,314
Word Fluency (Total)	32,5 +/− 10,6	28,8 +/− 10,4	30,7 +/−10,5	0,572
HADS-D				
Anxiety-Score	7,6 +/− 4,5	7,4 +/− 4,0	7,5 +/−4,2	0,572
Depression-Score	7,5 +/− 5,2	6,8 +/− 4,5	7,2 +/−4,7	0,572

## Discussion

We designed and initiated GACHE as a prospective multicentre, multinational, randomized, double-blind, placebo-controlled trial. Due to slow recruitment, the study was terminated prematurely. All centers recruited less patients than initially stated in the evaluation. Further studies should take into account the possibly overestimated incidence of the disease. The study was massively underpowered and its primary and secondary endpoints can only be interpreted with caution. Although there were no differences in mortality and the dichotomized mRS at 6 months between both groups, treatment with adjuvant dexamethasone did not have unexpected side effects which harmed patients in our study. Specifically, 21 patients suffering from HSVE were treated with combination of dexamethasone and aciclovir without serious side effects.

HSVE is a life threatening infectious disease of the central nervous system. Untreated HSVE mortality exceeds 70% and remains at 20% with antiviral treatment. Aciclovir, a synthetic guanosine analogue, is used for treating herpes simplex virus infection. Intravenous aciclovir provides modest bioavailability of 15–30% and is the first line treatment for HSVE [2–4]. One limitation of HSVE therapy is failure of early initiation of aciclovir therapy. Several studies indicate that in many cases, there is an unacceptable delay between clinical presentation and antiviral drug therapy. This delay is related to a poorer clinical outcome. The significant morbidity rates in HSVE warrant the active investigation of improved therapeutic approaches [2, 3, 5].

After introduction of antiviral therapy, reports indicated progressive tissue damage in later phases of the disease, also demonstrated in animal studies [6, 7]. Autoimmune mechanisms were proposed as a possible cause [1, 7]. Recently also immune-mediated antibody-driven mechanisms (e.g. anti NMDA-R-IgG, anti-N-type-VGCC) in a subgroup of patients following HSVE were described [21, 22].

In the pre-aciclovir era, corticosteroids were used in HSVE, because of their anti-inflammatory potency. Perceived but not proven corticosteroids effects were attributed to its suppressive mechanism concerning cerebral edema and local inflammation [2, 14, 15].

Currently the use of steroids in HSVE is an off-label treatment. A few case studies have concluded that patients treated with additional dexamethasone may have a better long term outcome [2, 16, 17]. There have been no large cohort studies or prospective trials indicating that the use of adjunctive steroids in HSVE improves long term outcome and inhibits ongoing tissue damage. GACHE is the first prospective multicenter, multinational, randomized, double blind trial studying the effect of adjuvant dexamethasone versus placebo on top of standard aciclovir treatment in adult patients, but was terminated due to slow recruitment. However, a study in UK analyzes additional dexamethasone in HSVE and is still on-going [23]. A prospective registry for patients receiving dexamethasone adjunct therapy may also help to further assess the safety of this treatment approach.

## Conclusion

GACHE studied the effect of adjuvant dexamethasone versus placebo on top of standard aciclovir treatment in adult patients aged 18 up to 85 years with proven HSVE in German academic centers of Neurology in a randomized and double blind fashion. GACHE being prematurely terminated demonstrated problems encountered performing randomized, placebo-controlled trials in life threatening neurological diseases. Unfortunately, the small number of study participants does not allow firm conclusions. Despite all this, GACHE shows the difficulties in patient recruitment for such a rare life-threatening disease. Based upon our trial results the use of adjuvant steroids in addition to antiviral treatment remains experimental and is at the decision of the individual treating physician.

## Additional file

Additional file 1: Statistic report. (PDF 4057 kb)

## Data Availability

The authors confirm that the data supporting the findings of this study are available within the article and its Additional file 1.

## Abbreviations

AVLT: Auditory Verbal Learning and Memory Test
CSF: Cerebrospinal fluid
EQ-5D: Quality of life as measured with the EuroQol 5D instrument
FAS: Full analysis set
GACHE: German trial of aciclovir and corticosteroids in Herpes simplex virus encephalitis
GCS: Glasgow coma scale score
GOS: Glasgow outcome scale score
HADS: Hospital anxiety and depression scale
HSVE: Herpes-simplex-virus-encephalitis
MMT: Mini-Mental Test
MRI: Magnetic resonance imaging
mRS: modified Ranking Scale
PCR: Polymerase chain reaction
PP: Per protocol
