# Supplementary material for: The German trial on Aciclovir and Corticosteroids in Herpes-simplex-virus-Encephalitis (GACHE): a multicenter, randomized, double-blind, placebo-controlled trial
Source: Neurol Res Pract. 2019 Sep 12;1:26. doi: 10.1186/s42466-019-0031-3 (PMC7650106; doi:10.1186/s42466-019-0031-3)
Supplement: Supplementary file 1 — Statistic report. (PDF 4057 kb) [file 42466_2019_31_MOESM1_ESM.pdf]

Contents

Signatures 2
1 Abbreviations and definitions 5
1.1 Tables 6
1.2 Figures 8
2 Objective of the trial 9
3 Study design 9
4 Treatment 9
5 Aims of the trial 9
6 Analysis sets 10
7 Data handling 10
8 Handling of unresolved data issues 10
9 Statistical methods 11
9.1 Demographics and baseline characteristics 11
9.2 Primary endpoint 11
9.2.1 Interim analysis 11
9.2.2 Sensitivity analyses for the primary endpoint 11
9.3 Secondary endpoints 12
9.4 Safety 13
9.5 Differences to trial protocol specified in the SAP 13
9.6 Analyses not preprespecified, more detailed specifications and differences to the SAP 14
9.7 Interpretation of results 14
9.8 Software 14
10 Results 15
10.1 Flow chart of enrolled patients 15
10.2 Inclusion/exclusion criteria 16
10.3 Demographics and baseline characteristics 17
10.3.1 Full analysis set 17
10.3.2 Per protocol set 28
10.4 Primary endpoint 38
10.4.1 Full analysis set 38
10.4.2 Sensitivity analyses 38

10.5 Secondary endpoints ... 40
10.5.1 Mortality ... 40
10.5.2 Glasgow outcome scale: GOS ... 42
10.5.3 Quality of life (EuroQol 5D) ... 43
10.5.4 Functional outcome (mRS) ... 47
10.5.5 Neuropsychological testing at month 6 ... 51
10.5.6 Barthel index ... 64
10.5.7 NIH-scale ... 74
10.6 Safety ... 92
10.6.1 Physical examination and vital signs ... 92
10.6.2 Adverse events ... 100
10.7 Applied study medication ... 125
10.7.1 Full analysis set ... 125
10.7.2 Per protocol set ... 126
10.8 Study termination ... 127
10.9 Other analyses ... 128
10.9.1 Seizures ... 128
11 References ... 129
12 Listings ... 130
12.1 Excluded patients ... 130
12.2 Adverse events/serious adverse event ... 132
13 Appendix ... 138
13.1 Original SAS® output for primary endpoint ... 138
Unresloved data issues ... 139

1 Abbreviations and definitions

Abbreviation	Definition
AE	Adverse Event
AVLT	Verbal Learning and Memory Test
BW	Body Weight
CF	Rey Complex Figure Test
EudraCT	European Union Drug Regulating Authorities Clinical Trials
FAS	Full analysis set
GOS	Glasgow Outcome Scale
HADS	Hospital Anxiety and Depression Scale
HLT	High Level Term
ITT	Intention to treat
LOCF	Last Observation Carried Forward
mRS	Modified Rankin Scale
MedDRA	Medical Dictionary for Regulatory Activities
PP	Per protocol
SAE	Serious Adverse Event
SAP	Statistical Analysis Plan
SD	Standard deviation
SOP	Standard Operating Procedure
SOC	System Organ Class
SUSAR	Suspected unexpected serious adverse reaction
TMT	Trail Making Test

1.1 Tables

Table 10.2.1: Listing of patients violating inclusion/exclusion criteria ... 16
Table 10.3.1: Demographics (FAS) ... 17
Table 10.3.2: Educational status (FAS) ... 17
Table 10.3.3: Vital signs (FAS) ... 18
Table 10.3.4: Physical examination (FAS) ... 19
Table 10.3.5: Diagnosis (FAS) ... 20
Table 10.3.6: Seizures (FAS) ... 20
Table 10.3.7: Listing of seizures (FAS) ... 21
Table 10.3.8: Glasgow Coma Scale on admission (FAS) ... 22
Table 10.3.9: Glasgow Coma Scale - pre-randomization (FAS) ... 23
Table 10.3.10: Pre-encephalitis Barthel index (FAS) ... 24
Table 10.3.11: Pre-encephalitis mRS (FAS) ... 25
Table 10.3.12: NIH scale day 0 (FAS) ... 25
Table 10.3.13: Mini-Mental test day 0 (FAS) ... 27
Table 10.3.14: Demographics (PP set) ... 28
Table 10.3.15: Educational status (PP set) ... 28
Table 10.3.16: Vital signs (PP set) ... 29
Table 10.3.17: Physical examination (PP set) ... 30
Table 10.3.18: Diagnosis (PP set) ... 31
Table 10.3.19: Seizures (PP set) ... 31
Table 10.3.20: Listing of seizures (PP set) ... 31
Table 10.3.21: Glasgow Coma Scale on admission (PP set) ... 32
Table 10.3.22: Glasgow Coma Scale - pre-randomization (PP set) ... 33
Table 10.3.23: Pre-encephalitis Barthel index (PP set) ... 34
Table 10.3.24: Pre-encephalitis mRS (PP set) ... 34
Table 10.3.25: NIH scale day 0 (PP set) ... 35
Table 10.3.26: Mini-Mental test day 0 (PP set) ... 37
Table 10.4.1: Binary mRS at scheduled month 6 visit – LOCF (FAS) ... 38
Table 10.4.2: Binary mRS at scheduled month 6 visit – best case imputation (FAS) ... 38
Table 10.4.3: Binary mRS at scheduled month 6 visit – worst case imputation (FAS) ... 38
Table 10.4.4: Binary mRS at month 6 (PP set) ... 39
Table 10.4.5: Binary mRS at month 6 +- 14 days – LOCF imputation (FAS) ... 39
Table 10.5.1: Mortality at month 6 (Chi²-test) (FAS) ... 40
Table 10.5.2: Mortality at month 12 (Chi²-test) (FAS) ... 40
Table 10.5.3: Mortality at month 6 (Chi²-test) (PP set) ... 41
Table 10.5.4: Mortality at month 12 (Chi²-test) (PP set) ... 41
Table 10.5.5: Glasgow outcome scale (FAS) ... 42
Table 10.5.6: Glasgow outcome scale (PP set) ... 42
Table 10.5.7: EQ-5D month 6 (FAS) ... 43
Table 10.5.8: EQ-5D month 12 (FAS) ... 44
Table 10.5.9: EQ-5D month 6 (PP set) ... 45

Table 10.5.10: EQ-5D month 12 (PP set) ... 46
Table 10.5.11: mRS at scheduled visit times (FAS) ... 47
Table 10.5.12: Binary mRS at scheduled visit month 12 (FAS) ... 47
Table 10.5.13: mRS at scheduled visit times (PP set) ... 49
Table 10.5.14: Binary mRS at scheduled visit times (PP set) ... 49
Table 10.5.15: Available reasons for missing neuropsychological testing ... 51
Table 10.5.16: Mini-Mental test (FAS) ... 52
Table 10.5.17: Complex Figure (FAS) ... 53
Table 10.5.18: Auditory Verbal Learning Test (FAS) ... 54
Table 10.5.19: Digit Span (FAS) ... 55
Table 10.5.20: Trail Making Test (FAS) ... 56
Table 10.5.21: Word Fluency (FAS) ... 56
Table 10.5.22: Hospital Anxiety and Depression Scale HADS-D (FAS) ... 57
Table 10.5.23: Categorized Hospital Anxiety and Depression Scale HADS-D (FAS) ... 57
Table 10.5.24: Mini-Mental test (PP set) ... 58
Table 10.5.25: Complex Figure (PP set) ... 59
Table 10.5.26: Auditory Verbal Learning Test (PP set) ... 60
Table 10.5.27: Digit Span (PP set) ... 61
Table 10.5.28: Trail Making test (PP set) ... 62
Table 10.5.29: Word Fluency (PP set) ... 62
Table 10.5.30: Hospital Anxiety and Depression Scale HADS-D (PP set) ... 63
Table 10.5.31: Categorized Hospital Anxiety and Depression Scale HADS-D (PP set) ... 63
Table 10.5.32: Barthel index total (FAS) ... 64
Table 10.5.33: Barthel index items at discharge/day 30 (FAS) ... 65
Table 10.5.34: Barthel index items at month 6 (FAS) ... 66
Table 10.5.35: Barthel index items at month 12 (FAS) ... 68
Table 10.5.36: Barthel index total (PP set) ... 69
Table 10.5.37: Barthel index items at discharge/day 30 (PP set) ... 70
Table 10.5.38: Barthel index items at month 6 (PP set) ... 71
Table 10.5.39: Barthel index items at month 12 (PP set) ... 73
Table 10.5.40: NIH total scores (FAS) ... 74
Table 10.5.41: NIH scale single items day 7 (FAS) ... 75
Table 10.5.42: NIH scale single items at discharge/day 30 (FAS) ... 77
Table 10.5.43: NIH scale single items at month 6 (FAS) ... 79
Table 10.5.44: NIH scale single items at month 12 (FAS) ... 81
Table 10.5.45: NIH total scores (PP set) ... 83
Table 10.5.46: NIH scale single items day 7 (PP set) ... 84
Table 10.5.47: NIH scale single items at discharge/day 30 (PP set) ... 86
Table 10.5.48: NIH scale single items at month 6 (PP set) ... 88
Table 10.5.49: NIH scale single items at month 12 (PP set) ... 90
Table 10.6.1: Vital signs day 7 (FAS) ... 92
Table 10.6.2: Vital signs at discharge/day 30 (FAS) ... 93
Table 10.6.3: Vital signs month 6 (FAS) ... 94

Table 10.6.4: Vital signs month 12 (FAS) 95
Table 10.6.5: Physical examination day 7 (FAS) 96
Table 10.6.6: Physical examination discharge/day 30 (FAS) 97
Table 10.6.7: Physical examination month 6 (FAS) 98
Table 10.6.8: Physical examination month 12 (FAS) 99
Table 10.6.9: Patients with (serious) adverse events 100
Table 10.6.10: Characteristics of serious adverse events 100
Table 10.6.11: Characteristics of adverse events 101
Table 10.6.12: System Organ Class (SOC) according to severity - group: Placebo 102
Table 10.6.13: System Organ Class (SOC) according to severity - group: Dexamethasone 103
Table 10.6.14: SOC/PT according to severity - group: Placebo 104
Table 10.6.15: SOC/PT according to severity - group: Dexamethasone 107
Table 10.6.16: System Organ Class (SOC) according to causality to study drug - group: Placebo 109
Table 10.6.17: System Organ Class (SOC) according to causality to study drug - group: Dexamethasone 110
Table 10.6.18: SOC / PT according to causality to study drug - group: Placebo 111
Table 10.6.19: SOC / PT according to causality to study drug - group: Dexamethasone 114
Table 10.6.20: System Organ Class (SOC) according to outcome - group: Placebo 117
Table 10.6.21: System Organ Class (SOC) according to outcome - group: Dexamethasone 118
Table 10.6.22: SOC / PT according to outcome - group: Placebo 119
Table 10.6.23: SOC / PT according to outcome - group: Dexamethasone 122
Table 10.7.1: Acyclovir administration (FAS) 125
Table 10.7.2: Dexamethasone/Placebo administration (FAS) 125
Table 10.7.3: Acyclovir administration (PP set) 126
Table 10.7.4: Dexamethasone/Placebo administration (PP set) 126
Table 10.8.1: Study termination (FAS) 127
Table 10.9.1: Seizures since last visit (FAS) 128
Table 10.9.2: Seizures since last visit (PP set) 128

1.2 Figures

Figure 10.5.1: Kaplan-Meier estimate for survival probability and p-value of log-rank test (FAS) 40
Figure 10.5.2: Kaplan-Meier estimate for survival probability and p-value of log-rank test (PP set) 41
Figure 10.5.3: Stacked bar plots mRS (FAS) 48
Figure 10.5.4: Stacked bar plots mRS (PP set) 50

2 Objective of the trial

GACHE aims to evaluate the effect of early adjuvant corticosteroids (dexamethasone) on morbidity and mortality in the treatment of adult patients with herpes-simplex-virusencephalitis.

3 Study design

The study design of GACHE is that of a multicenter, randomized, double-blind, placebo-controlled, parallel group clinical trial in adult patients with herpes-simplex-virus-encephalitis. The statistical design was planned as a group-sequential design with a maximum of three stages, rejection boundaries according to O´Brien and Fleming (1979) and a potential sample size adjustment for the last stage (Müller and Schäfer, 2001).
Due to slow recruitment, the study was terminated before the first interim analysis. The final analysis is conducted as done according to a fixed sample size design.

4 Treatment

Experimental group (‘Dexamethasone’):
10 mg/kg BW acyclovir (i.v., 1 hour infusion) every 8 hours for 14 days (dosage adaptation in case of decreased creatinine clearance) + dexamethasone 40 mg intravenously every 24 hours for 4 days

Control group (‘Placebo’):
10 mg/kg BW acyclovir (i.v., 1 hour infusion) every 8 hours for 14 days (dosage adaptation in case of decreased creatinine clearance) + Placebo every 24 hours for 4 days

5 Aims of the trial

Primary endpoint

The primary endpoint is the binary functional outcome 6 months after randomization (measured by the modified Rankin scale (mRS), a seven-point-scale 0-6). An mRS of 3 to 6 is seen as an unfavourable outcome (=failure). Patients who died between randomization and 6 months follow-up are evaluated with mRS=6. The mRS score at 6 months is derived based on the “Structured Interview for the Modified Rankin Scale” (Wilson, 2002).

Secondary endpoints

1. Mortality at 6 months after randomization.
2. Mortality at 12 months after randomization.
3. Functional outcome (Glasgow outcome scale: GOS) and quality of life (EuroQol 5D) 6 months after randomization.
4. Functional outcome (mRS: based on the “Structured Interview for the Modified Rankin Scale”, GOS) and quality of life (EuroQol 5D) 12 months after randomization.
5. Neuropsychological testing 6 months after randomization (defined in more detail later).
6. Barthel Index at discharge (at latest day 30), at 6 months visit and 12 months visit
7. NIH-scale at day 7, at discharge (at latest day 30), at 6 months visit and 12 months visit

6 Analysis sets

The full analysis set (FAS) based on the intention to treat principle (ITT) is the primary analysis set. It comprises all patients treated with at least one dose of study medication (acyclovir and dexamethasone/placebo) and analyzed in the group randomized to. A secondary analysis is performed as a repetition of the primary analysis using all patients who were treated and observed as outlined in the protocol. Patients are included into this per protocol (PP) analysis only if all inclusion and exclusion criteria are fulfilled and all medical procedures/visits have been carried out according to the protocol (dexamethasone/placebo over 4 days, if not dead: 6-month visit within ± 14 days). In case of randomization through randomization list without entering into the randomization tool afterwards, the patient is excluded from the PP set. The affiliation to the PP set in disputable cases was fixed in the document 'Unresolved Data Issues' prior to database closure (see section 'Unresloved data issues' there). The safety analysis set contains all patients who were treated at least for one day (acyclovir dexamethasone/placebo) and patients are categorized into groups as treated.

7 Data handling

Due to slow recruitment, the study was terminated after 55 months of recruitment. In this time, 42 randomizations have been carried out. One patient was randomized but PCR result for HSV encephalitis was negative. No data were documented for this patient. For one further patient, randomization was not performed via the randomization tool but next blinded study medication was given. This is in accordance to the procedure defined in the study protocol. However, the randomization entry created afterwards for this patient was incorrect. Therefore, this entry was deleted and a correct entry was created. For a further patient, the treatment code he was randomized to was expired and a new entry was necessary. In all, this resulted in 39 randomized patients. One further patient had been included into the study without randomization and without entering into the randomization tool afterwards. One patient was randomized into the study without legally valid written informed consent. After formal instruction, the data for this patient were deleted and CRFs were destroyed.

For reporting of SUSARs, the blind has been broken in two cases for managing the safety data. The blinding was maintained for the clinical study personal. One further case of unblinding was reported in the CRF (reason not given).

Missing values
For the ITT analysis set, missing values for the primary endpoint were imputed using the last-observation-carried-forward approach (LOCF). Patients who died between randomization and the 6 months visit were evaluated with mRS=6 at the 6 months visit. Patients who died between randomization and the 12 months visit were evaluated with mRS=6 at the 12 months visit. Imputation strategy for the Hospital Anxiety and Depression Scale (HADS) is described below. For further secondary endpoints no imputation was done. For the PP set no missing values were imputed.

8 Handling of unresolved data issues

The handling of remaining unresolved implausible values was fixed in the document 'Unresolved Data Issues' prior to database closure (see section 'Unresloved data issues' there). The unresolved data issues

are handled as described in this document. The mRS of patient 12-033 at visit 5 is set to 0 instead of 1 as stated in this document. This correction was made due to a typographic error in the document 'Unresolved data issues' and corresponds to the intention of the document.

For patient 07-102, no date of last contact was available. In this case, the date of last documented visit (visit 3) was used as date of last contact/last day in study.

9 Statistical methods

9.1 Demographics and baseline characteristics

Both treatment groups were characterized using descriptive methods based on the FAS and PP set. A flowchart is given to illustrate patient numbers included in the different analysis sets. Deviations from the inclusion and exclusion criteria were listed individually.

9.2 Primary endpoint

The primary endpoint analysis was based on the FAS and follows the intention-to-treat principle.
The null hypothesis of equal failure rates $p_{control}$ (acyclovir monotherapy group) and p_{exp} (group of acyclovir and corticosteroids) after 6 months was tested by a (two-sided) Chi²-test assessing the following test problem:

$$H_0:\ p_{control} = p_{exp} \quad vs. \qquad H_1:\ p_{control} \neq p_{exp}$$

The two-sided significance level is set to α=0.05.

In the Placebo group missing information on the primary endpoint was present in 2 patients and in the Dexamethason group in 5 patients. For primary analysis these values were imputed according to the last-observation-carried-forward approach (LOCF) (see section 7 Data handling).

In case of a significant result in the above described analysis, it was pre-planned to test the same null-hypothesis of equal rates for the first secondary endpoint (mortality after 6 months), applying the same significance level. Again, if this hypothesis can be rejected, the same null hypothesis should be tested for the second secondary endpoint (mortality after 12 months). Due to the a priori ordering of the three hypotheses, this strategy allows confirmatory conclusions for all tests without increase of the overall type I error rate. Since no significant difference for the primary endpoint could be shown, there was in fact no further testing performed.

9.2.1 Interim analysis

Due to unplanned termination of the study, no interim analysis was conducted. This final analysis was performed according to a fixed sample size design.

9.2.2 Sensitivity analyses for the primary endpoint

As sensitivity analysis, the primary endpoint was analyzed additionally based on the PP set in the same manner.
In addition, worst case and best case scenarios were evaluated based on the FAS meaning that in both treatment groups the worst (mRS>2) or best (mRS<=2) outcome was assumed in case of missing values.

Due to the small number of randomized patients and the partly high amount of missing data (e.g. for Glasgow Coma Scale at baseline) no further analyses like regression analyses were conducted.

9.3 Secondary endpoints

All secondary endpoints were analyzed based on the FAS and the PP set. In case of categorical variables, p-values correspond to the Chi²-test, in case of continuous variables, p-values are given for the non-parametric U-test.

Mortality
Mortality rates at 6 and 12 months are tabulated by treatment group. Corresponding p-values are provided for the Chi²-test. Additionally, Kaplan-Meier-curves by treatment group were used to graphically evaluate the survival rates over time together with results from the log rank tests.

Functional outcome and quality of life
Functional outcome (mRS, GOS) and quality of life (EuroQol 5D) were analyzed 6 months and 12 months after randomization, respectively.
The Glasgow outcome scale (five-point scale) and the mRS (seven-point scale) are tabulated by treatment group and time point. Corresponding p-values are given for the non-parametric U-test. For mRS, relative frequencies are depicted in stacked bar graphs.
Additionally, mRS after 12 months was dichotomized in the same manner as the primary outcome and tabulated by treatment group. The corresponding p-values are given for the Chi²-test.
EuroQol 5D overall is tabulated by treatment group and time point. Corresponding p-values are given for the non-parametric U-test.
Each of the five items of the EuroQol 5D is tabulated by treatment group and time point. Corresponding p-values are given for the non-parametric U-test.

Neuropsychological testing

All causes given in case of non-conducted neuropsychological testing are listed by test.

AVLT I and II (Verbal Learning and Memory Test): The sum of Dg 1-5, Dg 6 and Dg 7 are tabulated by treatment. Corresponding p-values are given for the non-parametric U-test.

CF (Rey Complex Figure Test): The Rey Complex Figure Tests were rated centrally by Dr. phil. Klaus Hess, Department of Neurology at the University Hospital Heidelberg. The score observed at each time point (copy, immediate recall, delayed recall) and the sum of first and second time point (copy, immediate recall) is tabulated by treatment group. If the second time point is missing, the score observed at the first time point corresponds to the sum of first and second time point. The corresponding p-value is given for the non-parametric U-test.

Digit Test: DS-f Gesamt, DS-b Gesamt, DS-f Spanne and DS-b Spanne are tabulated by treatment group. Corresponding p-values are given for the non-parametric U-test.

TMT (Trail Making Test): The reaction time is tabulated by treatment group. The corresponding p-value is given for the non-parametric U-test.

Word fluency: The sum of produced items (animal &food together) is tabulated by treatment group. The corresponding p-value is given for the non-parametric U-test.

Mini Mental Test: The total score and the sub-categories A-E are tabulated by treatment group. Corresponding p-values are given for the non-parametric U-test.

HADS (Hospital Anxiety and Depression Scale): This questionnaire defines two subscales, the anxiety score and the depression score. Each score was calculated adding up the values given for seven items. In case of one missing value within a subscale, this value was imputed using the mean value of the six other items. If more than one item was missing within a subscale, no imputation was done. The subscales were categorized into negative (0-7), neutral (8-10) and positive (≥ 11) and tabulated by treatment. Corresponding p-values are given for the Chi²-test.

Barthel Index:
The Barthel Index at discharge (at latest day 30), at 6 months visit and at 12 months visit is tabulated by treatment group and time. Corresponding p-values are given for the non-parametric U-test.

NIH-scale:
The NIH-scale at day 7, discharge (at latest day 30), at 6 months visit and at 12 months visit is tabulated by treatment group and time point. Corresponding p-values are given for the non-parametric U-test.

9.4 Safety

The main safety parameter is death which is analyzed as secondary endpoint. Additionally to that, death is analyzed in the same manner based on the safety analysis set.
Adverse events are tabulated by treatment group and overall by presenting number of events and number of subject with at least one event. Information on number of adverse events, SAEs, severity, causality and category (in summarized and detailed form) are given.

9.5 Differences to trial protocol specified in the SAP

In addition to the study protocol, Barthel Index and NIH-scale (neurological examination) were evaluated as secondary endpoints. The material-consuming Aachener Aphasietests were discarded from the list of neuropsychological tests and not undertaken because the neuropsychological impairment after >4 months is well examined with the other tests. The MRI at month 6 after randomization as well as the rate of occurrence of seizures were not analyzed.
Due to the limited number of patients, no logistic regression analyses was done.

9.6 Analyses not preprespecified, more detailed specifications and differences to the SAP

As intended by the SAP (GACHE Statistical Analysis Plan, 2014), the measured mRS was used independently of whether the actual visit was within ±14 days around the scheduled month 6 or month 12 visit, respectively. As sensitivity analysis, the mRS is set to missing and imputed using LOCF if the actual visit is not within ± 14 days around the scheduled visit.

Baseline characteristics and demographic data (see sections 9.1 and 1.1) are additionally shown for the PP set.

Due to the low number of cell counts and therefore questionable validity of the Chi^2-test, p-values for a conditional test are shown for the primary endpoint in addition to the pre-defined Chi^2 test.

The imputation of missing values (see section 7 in this document) was not restricted to patients that are 'lost to follow up' (as it has been stated in section 5 of the statistical analysis plan (GACHE Statistical Analysis Plan, 2014)).

The rate of seizures since last visit is shown in section 10.9 (Other analyses).

9.7 Interpretation of results

Due to slow recruitment, the study was terminated prematurely. With the lower sample size the planned power is not reached and all results can be interpreted only descriptively.

9.8 Software

All analyses have been done using validated SAS® Version 9.1 run on a Linux system. Stacked bar graphs for mRS as well as Kaplan-Meier plots have been created using SAS® Version 9.3.

10 Results

10.1 Flow chart of enrolled patients

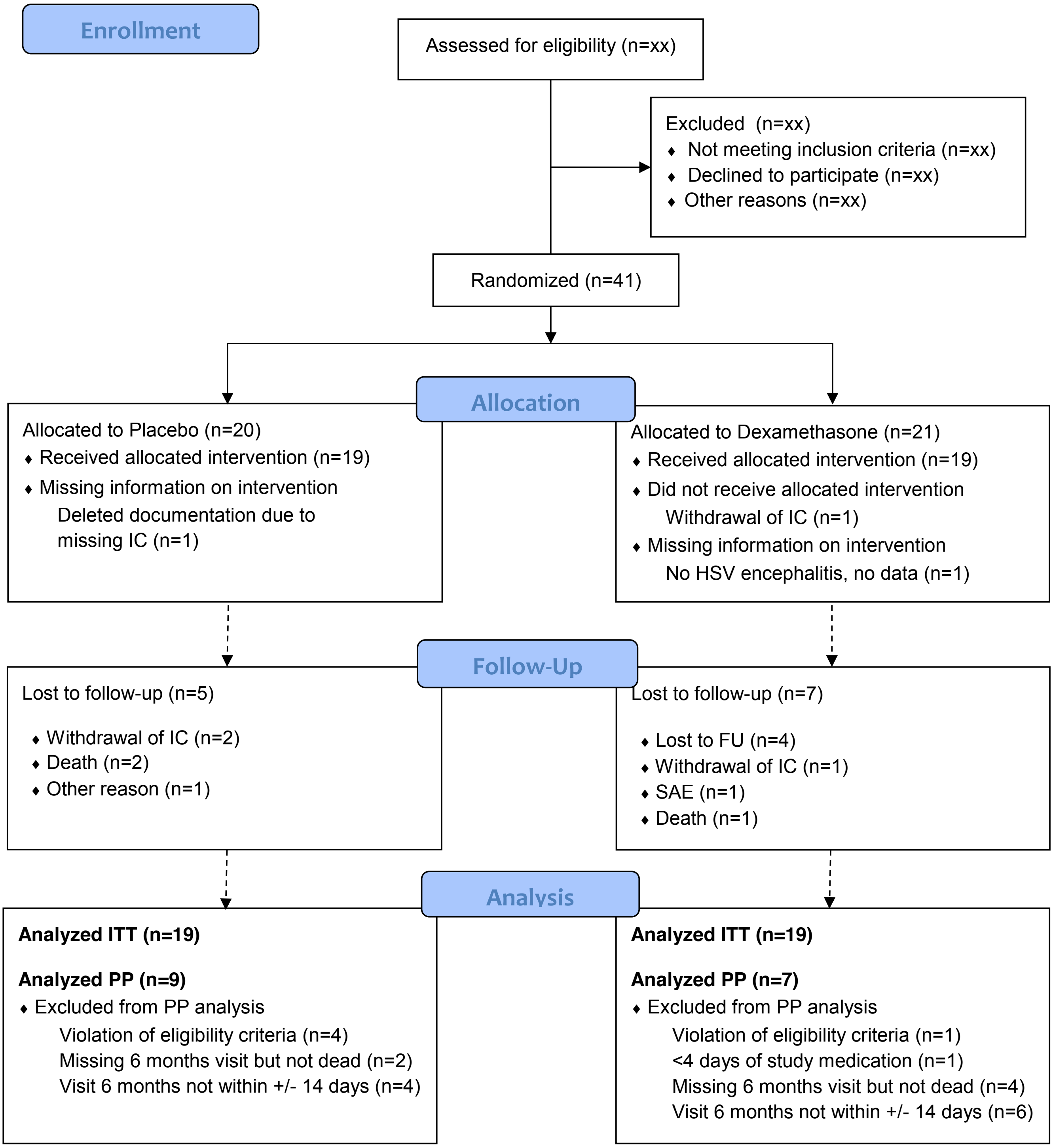

10.2 Inclusion/exclusion criteria

Two patients have been excluded from PP set due to documented violation of inclusion/exclusion criteria.

Table 10.2.1: Listing of patients violating inclusion/exclusion criteria

Patient	Age >=18 to <=85 years	Laboratory-proven HSVE	Focal neurological signs	Informed consent	Date of informed consent	Negative pregnacy testing	Systemic cortico-steroids	Hyper-sensitivity	Two fixed dilated pupils	mRS/ Barthel Index	Pregnancy	No highly effective birth control method	Breast feeding women
03-007	yes	yes	yes	yes	16/01/2012	na	yes	no	no	no	no	no	no
16-019	yes	yes	yes	yes	09/05/2011	yes	no	no	no	no	no	no	no

Patient	Tuberculosis/ systemic fungal infection	Head trauma/ neuro-surgery	Peptic ulcer disease	Acute viral infection other than HSVE	Life expectancy < 3 years	Other serious illness	Participation in another clinical trial	Previous Participation in another clinical trial	Previous Participation in this clinical trial
03-007	no	no	no	no	no	no	no	no	no
16-019	no	no	no	no	no	no	yes	no	no

In addition, 6 patients have contradictory documentations regarding inclusion/exclusion criteria as discussed in the document ‘Unresolved Data Issues’ (see section ‘Unresloved data issues’). 3 of these 6 patients have been excluded from PP set due to the contradictory documentation, and the other 3 patients have already been excluded for other reasons.

10.3 Demographics and baseline characteristics

10.3.1 Full analysis set

Table 10.3.1: Demographics (FAS)

	Placebo N=19	Dexamethasone N=19	Total N=38
Gender			
- male	10 (52.6%)	11 (57.9%)	21 (55.3%)
- female	9 (47.4%)	8 (42.1%)	17 (44.7%)
Age			
- N	19	19	38
- Mean +/- SD	58.6 +/-15.0	61.6 +/-12.1	60.1 +/-13.6
- Median	61.0	65.0	62.0
- p25, p75	43.0, 70.0	52.0, 72.0	49.0, 72.0
- Min, Max	37.0, 85.0	38.0, 77.0	37.0, 85.0
- Missing	0	0	0

Table 10.3.2: Educational status (FAS)

	Placebo N=19	Dexamethasone N=19	Total N=38
Höchster allgemeinbildender Schulabschluss			
- Hauptschulabschluss	6 (46.2%)	8 (61.5%)	14 (53.8%)
- Realschulabschluss	3 (23.1%)	2 (15.4%)	5 (19.2%)
- Polytechnische Oberschule 10. Klasse	2 (15.4%)	0 (0.0%)	2 (7.7%)
- Abitur	1 (7.7%)	3 (23.1%)	4 (15.4%)
- Anderer Schulabschluss	1 (7.7%)	0 (0.0%)	1 (3.8%)
- Missing	6	6	12
Beruflicher Ausbildungsabschluss			
- Keinen berufl. Abschluss, nicht in Ausbildung	2 (15.4%)	0 (0.0%)	2 (7.7%)
- Lehre abgeschlossen	5 (38.5%)	6 (46.2%)	11 (42.3%)
- Berufsfachschule, Handelsschule abgeschlossen	3 (23.1%)	2 (15.4%)	5 (19.2%)
- Fachschule, Meister-, Technikerschule, Berufs- oder Fachakademie abgeschlossen	3 (23.1%)	1 (7.7%)	4 (15.4%)
- Hochschulabschluss	0 (0.0%)	4 (30.8%)	4 (15.4%)
- Missing	6	6	12

Table 10.3.3: Vital signs (FAS)

	Placebo N=19	Dexamethasone N=19	Total N=38
Weight (kg)			
- N	16	17	33
- Mean +/- SD	78.3 +/-12.0	83.4 +/-18.5	80.9 +/-15.6
- Median	78.5	80.0	80.0
- p25, p75	71.8, 84.0	70.0, 84.0	70.0, 84.0
- Min, Max	56.5, 100.0	65.0, 126.0	56.5, 126.0
- Missing	3	2	5
BMI			
- N	14	16	30
- Mean +/- SD	25.9 +/-3.9	26.9 +/-3.5	26.5 +/-3.7
- Median	26.2	27.0	26.6
- p25, p75	21.9, 29.4	24.6, 29.0	24.2, 29.4
- Min, Max	19.1, 30.9	20.6, 33.2	19.1, 33.2
- Missing	5	3	8
Temperature (°C)			
- N	19	18	37
- Mean +/- SD	38.2 +/-0.8	37.7 +/-0.9	38.0 +/-0.9
- Median	38.3	37.4	38.0
- p25, p75	37.6, 38.6	36.9, 38.7	37.4, 38.6
- Min, Max	36.6, 40.0	36.5, 39.4	36.5, 40.0
- Missing	0	1	1
Heart rate (bpm)			
- N	18	18	36
- Mean +/- SD	85.9 +/-18.8	84.2 +/-18.9	85.1 +/-18.6
- Median	87.0	81.0	83.0
- p25, p75	74.0, 92.0	72.0, 90.0	72.0, 91.0
- Min, Max	59.0, 130.0	48.0, 126.0	48.0, 130.0
- Missing	1	1	2
Systolic blood pressure (mmHg)			
- N	19	18	37
- Mean +/- SD	133.4 +/-16.3	131.6 +/-18.6	132.5 +/-17.3
- Median	131.0	125.0	130.0
- p25, p75	120.0, 140.0	120.0, 150.0	120.0, 145.0
- Min, Max	105.0, 180.0	100.0, 164.0	100.0, 180.0
- Missing	0	1	1
Diastolic blood pressure (mmHg)			
- N	19	18	37
- Mean +/- SD	74.4 +/-11.9	73.4 +/-10.1	73.9 +/-10.9
- Median	75.0	74.0	75.0
- p25, p75	65.0, 80.0	60.0, 80.0	65.0, 80.0
- Min, Max	53.0, 100.0	60.0, 89.0	53.0, 100.0
- Missing	0	1	1

Table 10.3.4: Physical examination (FAS)

	Placebo<br>N=19	Dexamethasone<br>N=19	Total<br>N=38
General appearance			
- normal	18 (94.7%)	12 (63.2%)	30 (78.9%)
- abnormal	1 (5.3%)	7 (36.8%)	8 (21.1%)
Head & Neck			
- normal	18 (94.7%)	16 (84.2%)	34 (89.5%)
- abnormal	1 (5.3%)	2 (10.5%)	3 (7.9%)
- not done	0 (0.0%)	1 (5.3%)	1 (2.6%)
Eyes & Ears			
- normal	19 (100.0%)	14 (73.7%)	33 (86.8%)
- abnormal	0 (0.0%)	4 (21.1%)	4 (10.5%)
- not done	0 (0.0%)	1 (5.3%)	1 (2.6%)
Nose & Throat			
- normal	19 (100.0%)	17 (89.5%)	36 (94.7%)
- not done	0 (0.0%)	2 (10.5%)	2 (5.3%)
Chest			
- normal	16 (84.2%)	17 (89.5%)	33 (86.8%)
- abnormal	2 (10.5%)	1 (5.3%)	3 (7.9%)
- not done	1 (5.3%)	1 (5.3%)	2 (5.3%)
Lungs			
- normal	17 (89.5%)	18 (94.7%)	35 (92.1%)
- abnormal	2 (10.5%)	0 (0.0%)	2 (5.3%)
- not done	0 (0.0%)	1 (5.3%)	1 (2.6%)
Heart			
- normal	16 (84.2%)	17 (89.5%)	33 (86.8%)
- abnormal	3 (15.8%)	1 (5.3%)	4 (10.5%)
- not done	0 (0.0%)	1 (5.3%)	1 (2.6%)
Abdomen			
- normal	19 (100.0%)	17 (89.5%)	36 (94.7%)
- abnormal	0 (0.0%)	1 (5.3%)	1 (2.6%)
- not done	0 (0.0%)	1 (5.3%)	1 (2.6%)
Extremities & Joints			
- normal	18 (94.7%)	17 (89.5%)	35 (92.1%)
- abnormal	1 (5.3%)	1 (5.3%)	2 (5.3%)
- not done	0 (0.0%)	1 (5.3%)	1 (2.6%)
Lymph Nodes			
- normal	18 (94.7%)	18 (94.7%)	36 (94.7%)
- not done	1 (5.3%)	1 (5.3%)	2 (5.3%)
Skin			
- normal	16 (84.2%)	14 (73.7%)	30 (78.9%)
- abnormal	3 (15.8%)	4 (21.1%)	7 (18.4%)
- not done	0 (0.0%)	1 (5.3%)	1 (2.6%)

Table 10.3.5: Diagnosis (FAS)

	Placebo N=19	Dexamethasone N=19	Total N=38
Focal neurological signs			
- no	7 (36.8%)	4 (21.1%)	11 (28.9%)
- yes	12 (63.2%)	15 (78.9%)	27 (71.1%)
Result of polymerase chain reaction assay			
- positive	19 (100.0%)	19 (100.0%)	38 (100.0%)
Seizures within the last five days			
- no	8 (42.1%)	6 (31.6%)	14 (36.8%)
- yes	11 (57.9%)	11 (57.9%)	22 (57.9%)
- unknown	0 (0.0%)	2 (10.5%)	2 (5.3%)

Table 10.3.6: Seizures (FAS)

	Placebo N=19	Dexamethasone N=19	Total N=38
Focal continuously			
- no	10 (90.9%)	11 (100.0%)	21 (95.5%)
- yes	1 (9.1%)	0 (0.0%)	1 (4.5%)
Focal non continuously			
- no	10 (90.9%)	8 (72.7%)	18 (81.8%)
- yes	1 (9.1%)	3 (27.3%)	4 (18.2%)
Generalized continuously			
- no	10 (90.9%)	9 (81.8%)	19 (86.4%)
- yes	1 (9.1%)	2 (18.2%)	3 (13.6%)
Generalized non continuously			
- no	2 (18.2%)	4 (36.4%)	6 (27.3%)
- yes	9 (81.8%)	7 (63.6%)	16 (72.7%)

Table 10.3.7: Listing of seizures (FAS)

group	focal continuously	amount	focal non continuously	amount	generalized continuously	amount	generalized non continuously	amount
Dexamethasone	no	.	no	.	no	.	yes	K
Dexamethasone	no	.	no	.	yes	1	no	.
Placebo	no	.	yes	K	no	.	yes	K
Placebo	no	.	no	.	no	.	yes	1
Dexamethasone	no	.	yes	2	no	.	no	.
Dexamethasone	no	.	no	.	no	.	yes	1
Dexamethasone	no	.	no	.	no	.	yes	1
Placebo	no	.	no	.	no	.	yes	K
Dexamethasone	no	.	no	.	yes	1	yes	1
Dexamethasone	no	.	yes	1	no	.	no	.
Placebo	no	.	no	.	no	.	yes	1
Placebo	no	.	no	.	no	.	yes	1
Dexamethasone	no	.	no	.	no	.	yes	1
Dexamethasone	no	.	no	.	no	.	yes	1
Placebo	no	.	no	.	no	.	yes	2
Placebo	no	.	no	.	no	.	yes	1
Dexamethasone	no	.	no	.	no	.	yes	1
Placebo	yes	A	no	.	no	.	no	.
Placebo	no	.	no	.	yes	1	no	.
Placebo	no	.	no	.	no	.	yes	3
Placebo	no	.	no	.	no	.	yes	2
Dexamethasone	no	.	yes	1	no	.	no	.

Table 10.3.8: Glasgow Coma Scale on admission (FAS)

	Placebo N=19	Dexamethasone N=19	Total N=38
Glasgow Coma Scale on admission			
- not done	3 (15.8%)	7 (36.8%)	10 (26.3%)
- done	16 (84.2%)	12 (63.2%)	28 (73.7%)
Eye opening			
- 1-None	2 (12.5%)	0 (0.0%)	2 (7.4%)
- 3-To speech	5 (31.3%)	3 (27.3%)	8 (29.6%)
- 4-Spontaneous	9 (56.3%)	8 (72.7%)	17 (63.0%)
- Missing	3	8	11
Motor response			
- 1-None	2 (12.5%)	0 (0.0%)	2 (7.4%)
- 4-Withdrawal	1 (6.3%)	0 (0.0%)	1 (3.7%)
- 5-Localizes pain	3 (18.8%)	1 (9.1%)	4 (14.8%)
- 6-Obeys commands	10 (62.5%)	10 (90.9%)	20 (74.1%)
- Missing	3	8	11
Verbal response			
- 1-None	2 (12.5%)	0 (0.0%)	2 (7.7%)
- 2-Incomprehensible	1 (6.3%)	0 (0.0%)	1 (3.8%)
- 3-Inappropriate	1 (6.3%)	3 (30.0%)	4 (15.4%)
- 4-Confused	6 (37.5%)	5 (50.0%)	11 (42.3%)
- 5-Oriented	6 (37.5%)	2 (20.0%)	8 (30.8%)
- Missing	3	9	12
GCS Total			
- N	16	11	27
- Mean +/- SD	12.2 +/-3.9	12.8 +/-2.8	12.4 +/-3.4
- Median	13.0	13.0	13.0
- p25, p75	12.0, 15.0	13.0, 14.0	12.0, 15.0
- Min, Max	3.0, 15.0	5.0, 15.0	3.0, 15.0
- Missing	3	8	11

Table 10.3.9: Glasgow Coma Scale - pre-randomization (FAS)

	Placebo<br>N=19	Dexamethasone<br>N=19	Total<br>N=38
Eye opening			
- 1-None	2 (10.5%)	1 (5.3%)	3 (7.9%)
- 2-To pain	0 (0.0%)	1 (5.3%)	1 (2.6%)
- 3-To speech	4 (21.1%)	5 (26.3%)	9 (23.7%)
- 4-Spontaneous	13 (68.4%)	12 (63.2%)	25 (65.8%)
Motor response			
- 1-None	2 (10.5%)	1 (5.3%)	3 (7.9%)
- 2-Extension	0 (0.0%)	1 (5.3%)	1 (2.6%)
- 3-Flexor response	0 (0.0%)	1 (5.3%)	1 (2.6%)
- 4-Withdrawal	1 (5.3%)	1 (5.3%)	2 (5.3%)
- 5-Localizes pain	5 (26.3%)	1 (5.3%)	6 (15.8%)
- 6-Obeys commands	11 (57.9%)	14 (73.7%)	25 (65.8%)
Verbal response			
- 1-None	2 (11.1%)	3 (15.8%)	5 (13.5%)
- 2-Incomprehensible	3 (16.7%)	0 (0.0%)	3 (8.1%)
- 3-Inappropriate	0 (0.0%)	4 (21.1%)	4 (10.8%)
- 4-Confused	7 (38.9%)	7 (36.8%)	14 (37.8%)
- 5-Oriented	6 (33.3%)	5 (26.3%)	11 (29.7%)
- Missing	1	0	1
GCS Total			
- N	18	19	37
- Mean +/- SD	12.2 +/-3.7	12.3 +/-3.5	12.2 +/-3.6
- Median	13.5	13.0	13.0
- p25, p75	11.0, 15.0	12.0, 15.0	12.0, 15.0
- Min, Max	3.0, 15.0	3.0, 15.0	3.0, 15.0
- Missing	1	0	1

Table 10.3.10: Pre-encephalitis Barthel index (FAS)

	Placebo N=19	Dexamethasone N=19	Total N=38
Feeding			
- 0-unable	1 (5.3%)	0 (0.0%)	1 (2.6%)
- 5-needs help	1 (5.3%)	1 (5.3%)	2 (5.3%)
- 10-independent	17 (89.5%)	18 (94.7%)	35 (92.1%)
Bathing			
- 0-dependent	1 (5.3%)	1 (5.3%)	2 (5.3%)
- 5-independent	18 (94.7%)	18 (94.7%)	36 (94.7%)
Grooming			
- 0-needs to help	2 (10.5%)	1 (5.3%)	3 (7.9%)
- 5-independent	17 (89.5%)	18 (94.7%)	35 (92.1%)
Dressing			
- 0-dependent	1 (5.3%)	1 (5.3%)	2 (5.3%)
- 5-needs help	2 (10.5%)	0 (0.0%)	2 (5.3%)
- 10-independent	16 (84.2%)	18 (94.7%)	34 (89.5%)
Bowels			
- 0-incontinent	2 (10.5%)	1 (5.3%)	3 (7.9%)
- 10-continent	17 (89.5%)	18 (94.7%)	35 (92.1%)
Bladder			
- 0-incontinent/catheterized	2 (10.5%)	1 (5.3%)	3 (7.9%)
- 10-continent	17 (89.5%)	18 (94.7%)	35 (92.1%)
Toilet Use			
- 0-dependent	1 (5.3%)	1 (5.3%)	2 (5.3%)
- 5-needs some help	1 (5.3%)	0 (0.0%)	1 (2.6%)
- 10-independent	17 (89.5%)	18 (94.7%)	35 (92.1%)
Transfer (bed to chair and back)			
- 5-major help	1 (5.3%)	1 (5.3%)	2 (5.3%)
- 10-minor help	2 (10.5%)	0 (0.0%)	2 (5.3%)
- 15-independent	16 (84.2%)	18 (94.7%)	34 (89.5%)
Mobility (on level surfaces)			
- 0-immobile	1 (5.3%)	1 (5.3%)	2 (5.3%)
- 5-wheelchair independent	1 (5.3%)	0 (0.0%)	1 (2.6%)
- 10-walks with help	1 (5.3%)	0 (0.0%)	1 (2.6%)
- 15-independent	16 (84.2%)	18 (94.7%)	34 (89.5%)
Stairs			
- 0-unable	2 (10.5%)	1 (5.3%)	3 (7.9%)
- 10-independent	17 (89.5%)	18 (94.7%)	35 (92.1%)
Barthel Index Total			
- N	19	19	38
- Mean +/- SD	90.8 +/-24.3	95.3 +/-20.6	93.0 +/-22.3
- Median	100.0	100.0	100.0
- p25, p75	100.0, 100.0	100.0, 100.0	100.0, 100.0
- Min, Max	20.0, 100.0	10.0, 100.0	10.0, 100.0
- Missing	0	0	0

Table 10.3.11: Pre-encephalitis mRS (FAS)

	Placebo N=19	Dexamethasone N=19	Total N=38
Pre-encephalitis mRS			
- 0-No symptoms at all	17 (89.5%)	14 (73.7%)	31 (81.6%)
- 1-No significant disability	0 (0.0%)	4 (21.1%)	4 (10.5%)
- 2-Slight disability	2 (10.5%)	1 (5.3%)	3 (7.9%)

Table 10.3.12: NIH scale day 0 (FAS)

	Placebo N=19	Dexamethasone N=19	Total N=38
Level of consciousness			
- 0-Alert	6 (31.6%)	9 (47.4%)	15 (39.5%)
- 1-Drowsy	9 (47.4%)	7 (36.8%)	16 (42.1%)
- 2-Stuporous	2 (10.5%)	0 (0.0%)	2 (5.3%)
- 3-Coma	2 (10.5%)	3 (15.8%)	5 (13.2%)
Level of consciousness - questions			
- 0-Answers both correctly	5 (26.3%)	4 (21.1%)	9 (23.7%)
- 1-Answers one correctly	3 (15.8%)	7 (36.8%)	10 (26.3%)
- 2-Incorrect	11 (57.9%)	8 (42.1%)	19 (50.0%)
Level of consciousness - commands			
- 0-Obeys both correctly	8 (42.1%)	12 (63.2%)	20 (52.6%)
- 1-Obeys one correctly	6 (31.6%)	3 (15.8%)	9 (23.7%)
- 2-Incorrect	5 (26.3%)	4 (21.1%)	9 (23.7%)
Pupillary response			
- 0-Both reactive	18 (94.7%)	18 (94.7%)	36 (94.7%)
- 2-Neither reactive	1 (5.3%)	1 (5.3%)	2 (5.3%)
Best gaze			
- 0-Normal	18 (94.7%)	17 (89.5%)	35 (92.1%)
- 1-Partial gaze palsy	1 (5.3%)	1 (5.3%)	2 (5.3%)
- 2-Forced deviation	0 (0.0%)	1 (5.3%)	1 (2.6%)
Best visual			
- 0-No visual loss	19 (100.0%)	18 (94.7%)	37 (97.4%)
- 2-Complete hemianopia	0 (0.0%)	1 (5.3%)	1 (2.6%)
Facial palsy			
- 0-Normal	14 (73.7%)	17 (89.5%)	31 (81.6%)
- 1-Minor	3 (15.8%)	1 (5.3%)	4 (10.5%)
- 2-Partial	2 (10.5%)	0 (0.0%)	2 (5.3%)
- 3-Complete	0 (0.0%)	1 (5.3%)	1 (2.6%)
Best motor - arm			
- 0-No drift	13 (68.4%)	14 (73.7%)	27 (71.1%)
- 1-Drift	3 (15.8%)	2 (10.5%)	5 (13.2%)
- 2-Cannot resist gravity	1 (5.3%)	1 (5.3%)	2 (5.3%)
- 3-No effort against gravity	2 (10.5%)	2 (10.5%)	4 (10.5%)

	Placebo N=19	Dexamethasone N=19	Total N=38
Best motor - leg			
- 0-No drift	12 (63.2%)	15 (78.9%)	27 (71.1%)
- 1-Drift	3 (15.8%)	2 (10.5%)	5 (13.2%)
- 2-Cannot resist gravity	2 (10.5%)	0 (0.0%)	2 (5.3%)
- 3-No effort against gravity	2 (10.5%)	2 (10.5%)	4 (10.5%)
Plantar reflex			
- 0-Normal	14 (73.7%)	17 (89.5%)	31 (81.6%)
- 1-Equivocal	2 (10.5%)	0 (0.0%)	2 (5.3%)
- 2-One extensor	3 (15.8%)	1 (5.3%)	4 (10.5%)
- 3-Bilateral extensor	0 (0.0%)	1 (5.3%)	1 (2.6%)
Limb ataxia			
- 0-Absent	16 (84.2%)	18 (94.7%)	34 (89.5%)
- 1-Present in arm or leg	2 (10.5%)	1 (5.3%)	3 (7.9%)
- 2-Present in arm and leg	1 (5.3%)	0 (0.0%)	1 (2.6%)
Sensory			
- 0-Normal	15 (78.9%)	16 (84.2%)	31 (81.6%)
- 1-Partial loss	3 (15.8%)	2 (10.5%)	5 (13.2%)
- 2-Dense loss	1 (5.3%)	1 (5.3%)	2 (5.3%)
Neglect			
- 0-No neglect	17 (89.5%)	16 (84.2%)	33 (86.8%)
- 1-Partial neglect	0 (0.0%)	2 (10.5%)	2 (5.3%)
- 2-Complete neglect	2 (10.5%)	1 (5.3%)	3 (7.9%)
Dysarthria			
- 0-Normal articulation	13 (68.4%)	11 (57.9%)	24 (63.2%)
- 1-Mild to moderate dysarthria	3 (15.8%)	5 (26.3%)	8 (21.1%)
- 2-Near unitelliglible or worse	3 (15.8%)	3 (15.8%)	6 (15.8%)
Best language			
- 0-No aphasia	6 (31.6%)	4 (21.1%)	10 (26.3%)
- 1-Mild to moderate aphasia	5 (26.3%)	6 (31.6%)	11 (28.9%)
- 2-Severe aphasia	6 (31.6%)	6 (31.6%)	12 (31.6%)
- 3-Mute	2 (10.5%)	3 (15.8%)	5 (13.2%)
NIH Total			
- N	19	19	38
- Mean +/- SD	7.7 +/-6.6	6.9 +/-7.6	7.3 +/-7.0
- Median	6.0	5.0	6.0
- p25, p75	3.0, 10.0	2.0, 9.0	2.0, 9.0
- Min, Max	0.0, 23.0	0.0, 31.0	0.0, 31.0
- Missing	0	0	0

Table 10.3.13: Mini-Mental test day 0 (FAS)

	Placebo N=19	Dexamethasone N=19	Total N=38
Orientation			
- N	9	9	18
- Mean +/- SD	5.8 +/-4.5	5.1 +/-3.5	5.4 +/-3.9
- Median	8.0	5.0	6.0
- p25, p75	0.0, 9.0	3.0, 8.0	1.0, 9.0
- Min, Max	0.0, 10.0	0.0, 10.0	0.0, 10.0
- Missing	10	10	20
Immediate recall			
- N	9	9	18
- Mean +/- SD	1.9 +/-1.5	2.3 +/-1.1	2.1 +/-1.3
- Median	3.0	3.0	3.0
- p25, p75	0.0, 3.0	2.0, 3.0	1.0, 3.0
- Min, Max	0.0, 3.0	0.0, 3.0	0.0, 3.0
- Missing	10	10	20
Serial 7s total			
- N	9	9	18
- Mean +/- SD	2.4 +/-2.2	1.4 +/-1.7	1.9 +/-2.0
- Median	1.0	1.0	1.0
- p25, p75	1.0, 5.0	0.0, 2.0	0.0, 4.0
- Min, Max	0.0, 5.0	0.0, 4.0	0.0, 5.0
- Missing	10	10	20
Delayed verbal recall			
- N	9	9	18
- Mean +/- SD	0.6 +/-1.1	0.6 +/-1.0	0.6 +/-1.0
- Median	0.0	0.0	0.0
- p25, p75	0.0, 0.0	0.0, 1.0	0.0, 1.0
- Min, Max	0.0, 3.0	0.0, 3.0	0.0, 3.0
- Missing	10	10	20
Naming			
- N	9	9	18
- Mean +/- SD	6.0 +/-3.4	6.2 +/-3.3	6.1 +/-3.3
- Median	7.0	8.0	7.5
- p25, p75	5.0, 9.0	5.0, 9.0	5.0, 9.0
- Min, Max	0.0, 9.0	0.0, 9.0	0.0, 9.0
- Missing	10	10	20
Total score			
- N	9	9	18
- Mean +/- SD	16.7 +/-11.4	15.7 +/-9.3	16.2 +/-10.1
- Median	20.0	15.0	17.5
- p25, p75	7.0, 26.0	10.0, 24.0	7.0, 26.0
- Min, Max	0.0, 29.0	0.0, 27.0	0.0, 29.0
- Missing	10	10	20

10.3.2 Per protocol set

Table 10.3.14: Demographics (PP set)

	Placebo N=9	Dexamethasone N=7	Total N=16
Gender			
- male	7 (77.8%)	5 (71.4%)	12 (75.0%)
- female	2 (22.2%)	2 (28.6%)	4 (25.0%)
Age			
- N	9	7	16
- Mean +/- SD	59.6 +/-13.9	61.3 +/-11.1	60.3 +/-12.4
- Median	62.0	62.0	62.0
- p25, p75	51.0, 68.0	49.0, 71.0	50.0, 69.5
- Min, Max	39.0, 78.0	46.0, 77.0	39.0, 78.0
- Missing	0	0	0

Table 10.3.15: Educational status (PP set)

	Placebo N=9	Dexamethasone N=7	Total N=16
Höchster allgemeinbildender Schulabschluss			
- Hauptschulabschluss	3 (50.0%)	4 (66.7%)	7 (58.3%)
- Realschulabschluss	1 (16.7%)	1 (16.7%)	2 (16.7%)
- Polytechnische Oberschule 10. Klasse	1 (16.7%)	0 (0.0%)	1 (8.3%)
- Abitur	1 (16.7%)	1 (16.7%)	2 (16.7%)
- Missing	3	1	4
Beruflicher Ausbildungsabschluss			
- Lehre abgeschlossen	4 (66.7%)	3 (50.0%)	7 (58.3%)
- Berufsfachschule, Handelsschule abgeschlossen	1 (16.7%)	1 (16.7%)	2 (16.7%)
- Fachschule, Meister-, Technikerschule, Berufs- oder Fachakademie abgeschlossen	1 (16.7%)	1 (16.7%)	2 (16.7%)
- Hochschulabschluss	0 (0.0%)	1 (16.7%)	1 (8.3%)
- Missing	3	1	4

Table 10.3.16: Vital signs (PP set)

	Placebo N=9	Dexamethasone N=7	Total N=16
Weight (kg)			
- N	9	6	15
- Mean +/- SD	79.2 +/-9.8	86.0 +/-16.5	81.9 +/-12.8
- Median	77.0	84.0	80.0
- p25, p75	72.0, 81.0	70.0, 100.0	71.5, 88.0
- Min, Max	68.0, 100.0	68.0, 110.0	68.0, 110.0
- Missing	0	1	1
BMI			
- N	8	5	13
- Mean +/- SD	26.3 +/-3.8	27.3 +/-3.0	26.7 +/-3.4
- Median	26.0	27.7	26.6
- p25, p75	22.9, 30.2	25.1, 27.7	24.2, 29.4
- Min, Max	21.6, 30.9	24.2, 31.8	21.6, 31.8
- Missing	1	2	3
Temperature (°C)			
- N	9	6	15
- Mean +/- SD	38.0 +/-0.7	37.8 +/-1.0	37.9 +/-0.8
- Median	38.3	37.7	38.1
- p25, p75	37.6, 38.3	36.8, 38.8	37.4, 38.4
- Min, Max	36.6, 38.8	36.8, 39.1	36.6, 39.1
- Missing	0	1	1
Heart rate (bpm)			
- N	9	6	15
- Mean +/- SD	85.6 +/-22.9	85.0 +/-17.7	85.3 +/-20.3
- Median	76.0	81.0	76.0
- p25, p75	72.0, 93.0	70.0, 94.0	70.0, 94.0
- Min, Max	59.0, 130.0	70.0, 114.0	59.0, 130.0
- Missing	0	1	1
Systolic blood pressure (mmHg)			
- N	9	6	15
- Mean +/- SD	135.6 +/-10.6	145.5 +/-19.1	139.5 +/-14.8
- Median	140.0	152.0	140.0
- p25, p75	129.0, 140.0	130.0, 160.0	129.0, 150.0
- Min, Max	116.0, 150.0	115.0, 164.0	115.0, 164.0
- Missing	0	1	1
Diastolic blood pressure (mmHg)			
- N	9	6	15
- Mean +/- SD	74.7 +/-11.0	75.8 +/-9.8	75.1 +/-10.2
- Median	80.0	77.5	80.0
- p25, p75	75.0, 80.0	71.0, 80.0	71.0, 80.0
- Min, Max	53.0, 85.0	60.0, 89.0	53.0, 89.0
- Missing	0	1	1

Table 10.3.17: Physical examination (PP set)

	Placebo<br>N=9	Dexamethasone<br>N=7	Total<br>N=16
General appearance			
- normal	9 (100.0%)	5 (71.4%)	14 (87.5%)
- abnormal	0 (0.0%)	2 (28.6%)	2 (12.5%)
Head & Neck			
- normal	8 (88.9%)	5 (71.4%)	13 (81.3%)
- abnormal	1 (11.1%)	1 (14.3%)	2 (12.5%)
- not done	0 (0.0%)	1 (14.3%)	1 (6.3%)
Eyes & Ears			
- normal	9 (100.0%)	5 (71.4%)	14 (87.5%)
- abnormal	0 (0.0%)	1 (14.3%)	1 (6.3%)
- not done	0 (0.0%)	1 (14.3%)	1 (6.3%)
Nose & Throat			
- normal	9 (100.0%)	6 (85.7%)	15 (93.8%)
- not done	0 (0.0%)	1 (14.3%)	1 (6.3%)
Chest			
- normal	7 (77.8%)	6 (85.7%)	13 (81.3%)
- abnormal	1 (11.1%)	0 (0.0%)	1 (6.3%)
- not done	1 (11.1%)	1 (14.3%)	2 (12.5%)
Lungs			
- normal	8 (88.9%)	6 (85.7%)	14 (87.5%)
- abnormal	1 (11.1%)	0 (0.0%)	1 (6.3%)
- not done	0 (0.0%)	1 (14.3%)	1 (6.3%)
Heart			
- normal	6 (66.7%)	5 (71.4%)	11 (68.8%)
- abnormal	3 (33.3%)	1 (14.3%)	4 (25.0%)
- not done	0 (0.0%)	1 (14.3%)	1 (6.3%)
Abdomen			
- normal	9 (100.0%)	6 (85.7%)	15 (93.8%)
- not done	0 (0.0%)	1 (14.3%)	1 (6.3%)
Extremities & Joints			
- normal	9 (100.0%)	6 (85.7%)	15 (93.8%)
- not done	0 (0.0%)	1 (14.3%)	1 (6.3%)
Lymph Nodes			
- normal	9 (100.0%)	6 (85.7%)	15 (93.8%)
- not done	0 (0.0%)	1 (14.3%)	1 (6.3%)
Skin			
- normal	9 (100.0%)	3 (42.9%)	12 (75.0%)
- abnormal	0 (0.0%)	3 (42.9%)	3 (18.8%)
- not done	0 (0.0%)	1 (14.3%)	1 (6.3%)

Table 10.3.18: Diagnosis (PP set)

	Placebo N=9	Dexamethasone N=7	Total N=16
Focal neurological signs			
- no	3 (33.3%)	1 (14.3%)	4 (25.0%)
- yes	6 (66.7%)	6 (85.7%)	12 (75.0%)
Result of polymerase chain reaction assay			
- positive	9 (100.0%)	7 (100.0%)	16 (100.0%)
Seizures within the last five days			
- no	5 (55.6%)	3 (42.9%)	8 (50.0%)
- yes	4 (44.4%)	4 (57.1%)	8 (50.0%)

Table 10.3.19: Seizures (PP set)

	Placebo N=9	Dexamethasone N=7	Total N=16
Focal continuously			
- no	3 (75.0%)	4 (100.0%)	7 (87.5%)
- yes	1 (25.0%)	0 (0.0%)	1 (12.5%)
Focal non continuously			
- no	4 (100.0%)	2 (50.0%)	6 (75.0%)
- yes	0 (0.0%)	2 (50.0%)	2 (25.0%)
Generalized continuously			
- no	3 (75.0%)	4 (100.0%)	7 (87.5%)
- yes	1 (25.0%)	0 (0.0%)	1 (12.5%)
Generalized non continuously			
- no	2 (50.0%)	2 (50.0%)	4 (50.0%)
- yes	2 (50.0%)	2 (50.0%)	4 (50.0%)

Table 10.3.20: Listing of seizures (PP set)

group	focal continuously	amount	focal non continuously	amount	generalized continuously	amount	generalized non continuously	amount
Placebo	no	.	no	.	no	.	yes	1
Dexamethasone	no	.	yes	1	no	.	no	.
Placebo	no	.	no	.	no	.	yes	1
Dexamethasone	no	.	no	.	no	.	yes	1
Dexamethasone	no	.	no	.	no	.	yes	1
Placebo	yes	A	no	.	no	.	no	.
Placebo	no	.	no	.	yes	1	no	.
Dexamethasone	no	.	yes	1	no	.	no	.

Table 10.3.21: Glasgow Coma Scale on admission (PP set)

	Placebo N=9	Dexamethasone N=7	Total N=16
Glasgow Coma Scale on admission			
- not done	1 (11.1%)	3 (42.9%)	4 (25.0%)
- done	8 (88.9%)	4 (57.1%)	12 (75.0%)
Eye opening			
- 1-None	1 (12.5%)	0 (0.0%)	1 (8.3%)
- 3-To speech	2 (25.0%)	1 (25.0%)	3 (25.0%)
- 4-Spontaneous	5 (62.5%)	3 (75.0%)	8 (66.7%)
- Missing	1	3	4
Motor response			
- 1-None	1 (12.5%)	0 (0.0%)	1 (8.3%)
- 5-Localizes pain	2 (25.0%)	1 (25.0%)	3 (25.0%)
- 6-Obeys commands	5 (62.5%)	3 (75.0%)	8 (66.7%)
- Missing	1	3	4
Verbal response			
- 1-None	1 (12.5%)	0 (0.0%)	1 (8.3%)
- 3-Inappropriate	1 (12.5%)	1 (25.0%)	2 (16.7%)
- 4-Confused	3 (37.5%)	2 (50.0%)	5 (41.7%)
- 5-Oriented	3 (37.5%)	1 (25.0%)	4 (33.3%)
- Missing	1	3	4
GCS Total			
- N	8	4	12
- Mean +/- SD	12.4 +/-4.0	13.5 +/-1.3	12.8 +/-3.3
- Median	13.5	13.5	13.5
- p25, p75	12.0, 15.0	12.5, 14.5	12.0, 15.0
- Min, Max	3.0, 15.0	12.0, 15.0	3.0, 15.0
- Missing	1	3	4

Table 10.3.22: Glasgow Coma Scale - pre-randomization (PP set)

	Placebo<br>N=9	Dexamethasone<br>N=7	Total<br>N=16
Eye opening			
- 1-None	1 (11.1%)	0 (0.0%)	1 (6.3%)
- 3-To speech	1 (11.1%)	3 (42.9%)	4 (25.0%)
- 4-Spontaneous	7 (77.8%)	4 (57.1%)	11 (68.8%)
Motor response			
- 1-None	1 (11.1%)	0 (0.0%)	1 (6.3%)
- 3-Flexor response	0 (0.0%)	1 (14.3%)	1 (6.3%)
- 5-Localizes pain	3 (33.3%)	0 (0.0%)	3 (18.8%)
- 6-Obeys commands	5 (55.6%)	6 (85.7%)	11 (68.8%)
Verbal response			
- 1-None	1 (12.5%)	1 (14.3%)	2 (13.3%)
- 2-Incomprehensible	1 (12.5%)	0 (0.0%)	1 (6.7%)
- 3-Inappropriate	0 (0.0%)	1 (14.3%)	1 (6.7%)
- 4-Confused	3 (37.5%)	3 (42.9%)	6 (40.0%)
- 5-Oriented	3 (37.5%)	2 (28.6%)	5 (33.3%)
- Missing	1	0	1
GCS Total			
- N	8	7	15
- Mean +/- SD	12.3 +/-4.0	12.9 +/-2.7	12.5 +/-3.4
- Median	13.5	13.0	13.0
- p25, p75	11.5, 15.0	13.0, 15.0	12.0, 15.0
- Min, Max	3.0, 15.0	7.0, 15.0	3.0, 15.0
- Missing	1	0	1

Table 10.3.23: Pre-encephalitis Barthel index (PP set)

	Placebo N=9	Dexamethasone N=7	Total N=16
Feeding			
- 10-independent	9 (100.0%)	7 (100.0%)	16 (100.0%)
Bathing			
- 5-independent	9 (100.0%)	7 (100.0%)	16 (100.0%)
Grooming			
- 5-independent	9 (100.0%)	7 (100.0%)	16 (100.0%)
Dressing			
- 10-independent	9 (100.0%)	7 (100.0%)	16 (100.0%)
Bowels			
- 10-continent	9 (100.0%)	7 (100.0%)	16 (100.0%)
Bladder			
- 10-continent	9 (100.0%)	7 (100.0%)	16 (100.0%)
Toilet Use			
- 10-independent	9 (100.0%)	7 (100.0%)	16 (100.0%)
Transfer (bed to chair and back)			
- 15-independent	9 (100.0%)	7 (100.0%)	16 (100.0%)
Mobility (on level surfaces)			
- 15-independent	9 (100.0%)	7 (100.0%)	16 (100.0%)
Stairs			
- 10-independent	9 (100.0%)	7 (100.0%)	16 (100.0%)
Barthel Index Total			
- N	9	7	16
- Mean +/- SD	100.0 +/-0.0	100.0 +/-0.0	100.0 +/-0.0
- Median	100.0	100.0	100.0
- p25, p75	100.0, 100.0	100.0, 100.0	100.0, 100.0
- Min, Max	100.0, 100.0	100.0, 100.0	100.0, 100.0
- Missing	0	0	0

Table 10.3.24: Pre-encephalitis mRS (PP set)

	Placebo N=9	Dexamethasone N=7	Total N=16
Pre-encephalitis mRS			
- 0-No symptoms at all	8 (88.9%)	6 (85.7%)	14 (87.5%)
- 1-No significant disability	0 (0.0%)	1 (14.3%)	1 (6.3%)
- 2-Slight disability	1 (11.1%)	0 (0.0%)	1 (6.3%)

Table 10.3.25: NIH scale day 0 (PP set)

	Placebo N=9	Dexamethasone N=7	Total N=16
Level of consciousness			
- 0-Alert	6 (66.7%)	2 (28.6%)	8 (50.0%)
- 1-Drowsy	2 (22.2%)	4 (57.1%)	6 (37.5%)
- 3-Coma	1 (11.1%)	1 (14.3%)	2 (12.5%)
Level of consciousness - questions			
- 0-Answers both correctly	3 (33.3%)	2 (28.6%)	5 (31.3%)
- 1-Answers one correctly	1 (11.1%)	2 (28.6%)	3 (18.8%)
- 2-Incorrect	5 (55.6%)	3 (42.9%)	8 (50.0%)
Level of consciousness - commands			
- 0-Obeys both correctly	4 (44.4%)	4 (57.1%)	8 (50.0%)
- 1-Obeys one correctly	2 (22.2%)	2 (28.6%)	4 (25.0%)
- 2-Incorrect	3 (33.3%)	1 (14.3%)	4 (25.0%)
Pupillary response			
- 0-Both reactive	9 (100.0%)	7 (100.0%)	16 (100.0%)
Best gaze			
- 0-Normal	9 (100.0%)	6 (85.7%)	15 (93.8%)
- 1-Partial gaze palsy	0 (0.0%)	1 (14.3%)	1 (6.3%)
Best visual			
- 0-No visual loss	9 (100.0%)	7 (100.0%)	16 (100.0%)
Facial palsy			
- 0-Normal	6 (66.7%)	7 (100.0%)	13 (81.3%)
- 1-Minor	2 (22.2%)	0 (0.0%)	2 (12.5%)
- 2-Partial	1 (11.1%)	0 (0.0%)	1 (6.3%)
Best motor - arm			
- 0-No drift	7 (77.8%)	6 (85.7%)	13 (81.3%)
- 1-Drift	1 (11.1%)	0 (0.0%)	1 (6.3%)
- 3-No effort against gravity	1 (11.1%)	1 (14.3%)	2 (12.5%)
Best motor - leg			
- 0-No drift	8 (88.9%)	6 (85.7%)	14 (87.5%)
- 3-No effort against gravity	1 (11.1%)	1 (14.3%)	2 (12.5%)
Plantar reflex			
- 0-Normal	9 (100.0%)	7 (100.0%)	16 (100.0%)
Limb ataxia			
- 0-Absent	7 (77.8%)	6 (85.7%)	13 (81.3%)
- 1-Present in arm or leg	2 (22.2%)	1 (14.3%)	3 (18.8%)
Sensory			
- 0-Normal	9 (100.0%)	6 (85.7%)	15 (93.8%)
- 1-Partial loss	0 (0.0%)	1 (14.3%)	1 (6.3%)
Neglect			
- 0-No neglect	9 (100.0%)	6 (85.7%)	15 (93.8%)
- 1-Partial neglect	0 (0.0%)	1 (14.3%)	1 (6.3%)

	Placebo N=9	Dexamethasone N=7	Total N=16
Dysarthria			
- 0-Normal articulation	5 (55.6%)	4 (57.1%)	9 (56.3%)
- 1-Mild to moderate dysarthria	3 (33.3%)	2 (28.6%)	5 (31.3%)
- 2-Near unitelliglible or worse	1 (11.1%)	1 (14.3%)	2 (12.5%)
Best language			
- 0-No aphasia	2 (22.2%)	1 (14.3%)	3 (18.8%)
- 1-Mild to moderate aphasia	3 (33.3%)	3 (42.9%)	6 (37.5%)
- 2-Severe aphasia	3 (33.3%)	2 (28.6%)	5 (31.3%)
- 3-Mute	1 (11.1%)	1 (14.3%)	2 (12.5%)
NIH Total			
- N	9	7	16
- Mean +/- SD	6.0 +/-5.8	6.1 +/-5.4	6.1 +/-5.4
- Median	6.0	5.0	5.5
- p25, p75	2.0, 8.0	1.0, 8.0	1.5, 8.0
- Min, Max	0.0, 19.0	1.0, 17.0	0.0, 19.0
- Missing	0	0	0

Table 10.3.26: Mini-Mental test day 0 (PP set)

	Placebo N=9	Dexamethasone N=7	Total N=16
Orientation			
- N	6	3	9
- Mean +/- SD	6.3 +/-4.9	6.3 +/-1.5	6.3 +/-4.0
- Median	9.0	6.0	8.0
- p25, p75	0.0, 10.0	5.0, 8.0	5.0, 9.0
- Min, Max	0.0, 10.0	5.0, 8.0	0.0, 10.0
- Missing	3	4	7
Immediate recall			
- N	6	3	9
- Mean +/- SD	2.0 +/-1.5	2.7 +/-0.6	2.2 +/-1.3
- Median	3.0	3.0	3.0
- p25, p75	0.0, 3.0	2.0, 3.0	2.0, 3.0
- Min, Max	0.0, 3.0	2.0, 3.0	0.0, 3.0
- Missing	3	4	7
Serial 7s total			
- N	6	3	9
- Mean +/- SD	2.7 +/-2.3	2.3 +/-1.5	2.6 +/-1.9
- Median	2.5	2.0	2.0
- p25, p75	1.0, 5.0	1.0, 4.0	1.0, 4.0
- Min, Max	0.0, 5.0	1.0, 4.0	0.0, 5.0
- Missing	3	4	7
Delayed verbal recall			
- N	6	3	9
- Mean +/- SD	0.8 +/-1.3	0.0 +/-0.0	0.6 +/-1.1
- Median	0.0	0.0	0.0
- p25, p75	0.0, 2.0	0.0, 0.0	0.0, 0.0
- Min, Max	0.0, 3.0	0.0, 0.0	0.0, 3.0
- Missing	3	4	7
Naming			
- N	6	3	9
- Mean +/- SD	6.7 +/-3.0	7.7 +/-1.5	7.0 +/-2.5
- Median	7.5	8.0	8.0
- p25, p75	6.0, 9.0	6.0, 9.0	6.0, 9.0
- Min, Max	1.0, 9.0	6.0, 9.0	1.0, 9.0
- Missing	3	4	7
Total score			
- N	6	3	9
- Mean +/- SD	18.5 +/-11.8	19.0 +/-5.0	18.7 +/-9.7
- Median	23.0	19.0	20.0
- p25, p75	7.0, 28.0	14.0, 24.0	14.0, 26.0
- Min, Max	1.0, 29.0	14.0, 24.0	1.0, 29.0
- Missing	3	4	7

10.4 Primary endpoint

10.4.1 Full analysis set

Table 10.4.1: Binary mRS at scheduled month 6 visit – LOCF (FAS)

	Placebo		Dexamethasone		All		p-value
Binary mRS							
- <=2	12	(63.2%)	12	(63.2%)	24	(63.2%)	1.0000
- >2	7	(36.8%)	7	(36.8%)	14	(36.8%)	

*p-value of unconditional test is 1.0

There seem to be no differences in the binary mRS outcome scale between Placebo and Dexamethasone group. However, due to the preliminary termination of the trial and the resulting low sample size these results have only descriptive character.

10.4.2 Sensitivity analyses

Table 10.4.2: Binary mRS at scheduled month 6 visit – best case imputation (FAS)

	Placebo		Dexamethasone		All		p-value
Binary mRS	.						
- <=2	14	(73.7%)	15	(78.9%)	29	(76.3%)	0.7028
- >2	5	(26.3%)	4	(21.1%)	9	(23.7%)	

*p-value of unconditional test is 0.8107

Table 10.4.3: Binary mRS at scheduled month 6 visit – worst case imputation (FAS)

	Placebo		Dexamethasone		All		p-value
Binary mRS							
- <=2	12	(63.2%)	10	(52.6%)	22	(57.9%)	0.5111
- >2	7	(36.8%)	9	(47.4%)	16	(42.1%)	

*p-value of unconditional test is 0.5923

Table 10.4.4: Binary mRS at month 6 (PP set)

	Placebo		Dexamethasone		All		p-value
Binary mRS							
- <=2	7	(77.8%)	4	(57.1%)	11	(68.8%)	0.3770*
- >2	2	(22.2%)	3	(42.9%)	5	(31.3%)	

*p-value of unconditional test is 0.5622

Table 10.4.5: Binary mRS at month 6 +- 14 days – LOCF imputation (FAS)

	Placebo		Dexamethasone		All		p-value
Binary mRS							
- <=2	10	(52.6%)	10	(52.6%)	20	(52.6%)	1.0000*
- >2	9	(47.4%)	9	(47.4%)	18	(47.4%)	

*p-value of unconditional test is 1.0

All sensitivity analyses confirm the results of the primary analysis.

10.5 Secondary endpoints

10.5.1 Mortality

10.5.1.1 FAS

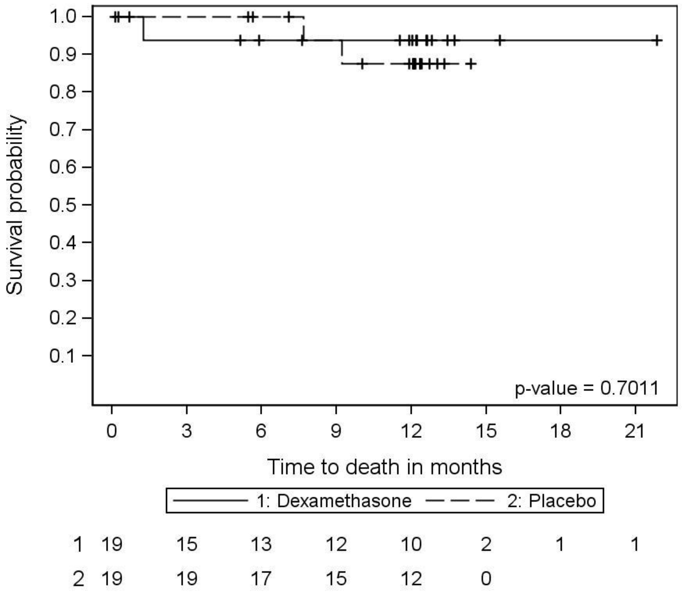

Figure 10.5.1: Kaplan-Meier estimate for survival probability and p-value of log-rank test (FAS). **Numbers below the plot correspond to patients at risk.**

Table 10.5.1: Mortality at month 6 (Chi²-test) (FAS)

	Placebo	Dexamethasone	All	p-value
- Alive	18 (100.0%)	14 (93.3%)	32 (97.0%)	0.2660
- Dead	0 (0.0%)	1 (6.7%)	1 (3.0%)	
- Missing	1	4	5	

Table 10.5.2: Mortality at month 12 (Chi²-test) (FAS)

	Placebo	Dexamethasone	All	p-value
- Alive	13 (86.7%)	11 (91.7%)	24 (88.9%)	0.6812
- Dead	2 (13.3%)	1 (8.3%)	3 (11.1%)	
- Missing	4	7	11	

10.5.1.2 PP set

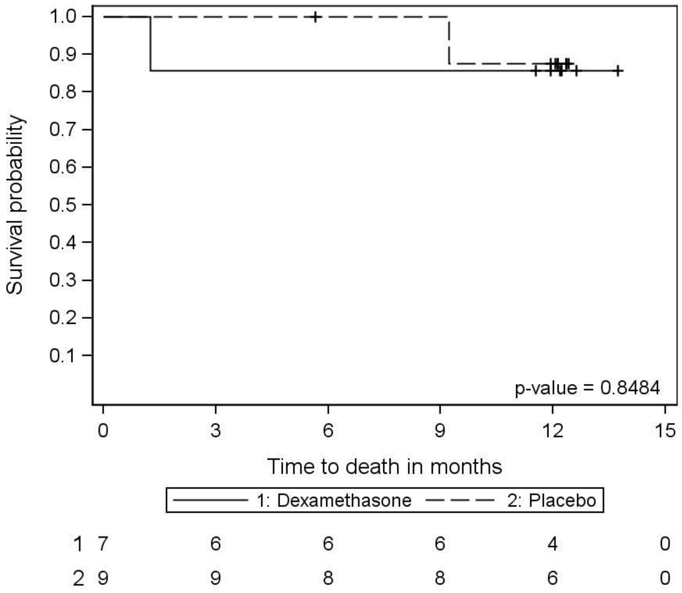

Figure 10.5.2: Kaplan-Meier estimate for survival probability and p-value of log-rank test (PP set).
Numbers below the plot correspond to patients at risk.

Table 10.5.3: Mortality at month 6 (Chi²-test) (PP set)

	Placebo		Dexamethasone		All		p-value
- Alive	9	(100.0%)	6	(85.7%)	15	(93.8%)	0.2416
- Dead	0	(0.0%)	1	(14.3%)	1	(6.3%)	
- Missing	0		0		0		

Table 10.5.4: Mortality at month 12 (Chi²-test) (PP set)

	Placebo		Dexamethasone		All		p-value
- Alive	7	(87.5%)	5	(83.3%)	12	(85.7%)	0.8255
- Dead	1	(12.5%)	1	(16.7%)	2	(14.3%)	
- Missing	1		1		2		

10.5.2 Glasgow outcome scale: GOS

10.5.2.1 FAS

Table 10.5.5: Glasgow outcome scale (FAS)

	Placebo N=19	Dexamethasone N=19	Total N=38	p-value
Discharge/Day 30 GOS*				
- 2-Persistant vegetative state	0 (0.0%)	1 (5.9%)	1 (2.9%)	0.426 *[1]
- 3-Severe disabiliy	9 (50.0%)	5 (29.4%)	14 (40.0%)	
- 4-Moderate disabiliy	7 (38.9%)	7 (41.2%)	14 (40.0%)	
- 5-Good recovery	2 (11.1%)	4 (23.5%)	6 (17.1%)	
- Missing	1	2	3	
6 Months GOS				
- 3-Severe disabiliy	3 (17.6%)	1 (7.7%)	4 (13.3%)	0.460 *[1]
- 4-Moderate disabiliy	6 (35.3%)	3 (23.1%)	9 (30.0%)	
- 5-Good recovery	8 (47.1%)	9 (69.2%)	17 (56.7%)	
- Missing	2	6	8	
12 Months GOS				
- 3-Severe disabiliy	2 (14.3%)	2 (16.7%)	4 (15.4%)	0.773 *[1]
- 4-Moderate disabiliy	4 (28.6%)	2 (16.7%)	6 (23.1%)	
- 5-Good recovery	8 (57.1%)	8 (66.7%)	16 (61.5%)	
- Missing	5	7	12	

* = no secondary endpoint, *[1] = Chi^2-test, two-sided

10.5.2.2 PP set

Table 10.5.6: Glasgow outcome scale (PP set)

	Placebo N=9	Dexamethasone N=7	Total N=16	p-value
Discharge/Day 30 GOS				
- 3-Severe disabiliy	2 (25.0%)	2 (40.0%)	4 (30.8%)	0.465 *[1]
- 4-Moderate disabiliy	4 (50.0%)	3 (60.0%)	7 (53.8%)	
- 5-Good recovery	2 (25.0%)	0 (0.0%)	2 (15.4%)	
- Missing	1	2	3	
6 Months GOS				
- 3-Severe disabiliy	1 (11.1%)	0 (0.0%)	1 (6.7%)	0.446 *[1]
- 4-Moderate disabiliy	1 (11.1%)	2 (33.3%)	3 (20.0%)	
- 5-Good recovery	7 (77.8%)	4 (66.7%)	11 (73.3%)	
- Missing	0	1	1	
12 Months GOS				
- 4-Moderate disabiliy	2 (28.6%)	2 (33.3%)	4 (30.8%)	0.853 *[1]
- 5-Good recovery	5 (71.4%)	4 (66.7%)	9 (69.2%)	
- Missing	2	1	3	

*[1] = Chi^2-test, two-sided

10.5.3 Quality of life (EuroQol 5D)

10.5.3.1 FAS

Table 10.5.7: EQ-5D month 6 (FAS)

	Placebo N=19	Dexamethasone N=19	Total N=38	p-value
Mobility				
- no problems in walking about	11 (100.0%)	6 (75.0%)	17 (89.5%)	0.080 *1
- some problems in walking about	0 (0.0%)	2 (25.0%)	2 (10.5%)	
- Missing	8	11	19	
Self-care				
- no problems with self-care	11 (100.0%)	6 (75.0%)	17 (89.5%)	0.080 *1
- some problems washing and dressing	0 (0.0%)	2 (25.0%)	2 (10.5%)	
- Missing	8	11	19	
Usual activities				
- no problems with performing usual activities	4 (36.4%)	4 (50.0%)	8 (42.1%)	0.336 *1
- some problems with permorming usual activities	7 (63.6%)	3 (37.5%)	10 (52.6%)	
- unable to perform usual activities	0 (0.0%)	1 (12.5%)	1 (5.3%)	
- Missing	8	11	19	
Pain/discomfort				
- no pain or discomfort	10 (90.9%)	5 (62.5%)	15 (78.9%)	0.134 *1
- moderate pain or discomfort	1 (9.1%)	3 (37.5%)	4 (21.1%)	
- Missing	8	11	19	
Anxiety/depression				
- not anxious or depressed	8 (72.7%)	4 (50.0%)	12 (63.2%)	0.385 *1
- moderately anxious or depressed	3 (27.3%)	3 (37.5%)	6 (31.6%)	
- extremely anxious or depressed	0 (0.0%)	1 (12.5%)	1 (5.3%)	
- Missing	8	11	19	
EQ-5D Index				
- N	11	8	19	0.243 *2
- Mean +/- SD	1.0 +/-0.0	0.8 +/-0.2	0.9 +/-0.2	
- Median	1.0	0.9	1.0	
- p25, p75	1.0, 1.0	0.7, 1.0	0.9, 1.0	
- Min, Max	0.9, 1.0	0.4, 1.0	0.4, 1.0	
- Missing	8	11	19	
EQ-5D VAS				
- N	11	8	19	0.184 *2
- Mean +/- SD	71.3 +/-12.7	62.0 +/-14.1	67.4 +/-13.7	
- Median	75.0	58.0	70.0	
- p25, p75	60.0, 80.0	55.0, 72.5	55.0, 79.0	
- Min, Max	50.0, 85.0	40.0, 85.0	40.0, 85.0	
- Missing	8	11	19	

*1 = Chi^2-test, two-sided, *2 = U-test, two-sided

Table 10.5.8: EQ-5D month 12 (FAS)

	Placebo N=19	Dexamethasone N=19	Total N=38	p-value
Mobility				
- no problems in walking about	7 (87.5%)	5 (71.4%)	12 (80.0%)	0.529 *[1]
- some problems in walking about	1 (12.5%)	1 (14.3%)	2 (13.3%)	
- confined to bed	0 (0.0%)	1 (14.3%)	1 (6.7%)	
- Missing	11	12	23	
Self-care				
- no problems with self-care	8 (100.0%)	6 (85.7%)	14 (93.3%)	0.268 *[1]
- unable to wash and dress	0 (0.0%)	1 (14.3%)	1 (6.7%)	
- Missing	11	12	23	
Usual activities				
- no problems with performing usual activities	4 (50.0%)	4 (57.1%)	8 (53.3%)	0.782 *[1]
- some problems with permorming usual activities	4 (50.0%)	3 (42.9%)	7 (46.7%)	
- Missing	11	12	23	
Pain/discomfort				
- no pain or discomfort	6 (75.0%)	4 (57.1%)	10 (66.7%)	0.464 *[1]
- moderate pain or discomfort	2 (25.0%)	3 (42.9%)	5 (33.3%)	
- Missing	11	12	23	
Anxiety/depression				
- not anxious or depressed	5 (62.5%)	5 (71.4%)	10 (66.7%)	0.875 *[1]
- moderately anxious or depressed	2 (25.0%)	1 (14.3%)	3 (20.0%)	
- extremely anxious or depressed	1 (12.5%)	1 (14.3%)	2 (13.3%)	
- Missing	11	12	23	
EQ-5D Index				
- N	8	7	15	0.812 *[2]
- Mean +/- SD	0.9 +/-0.2	0.8 +/-0.4	0.8 +/-0.3	
- Median	1.0	0.9	1.0	
- p25, p75	0.9, 1.0	0.5, 1.0	0.9, 1.0	
- Min, Max	0.5, 1.0	0.1, 1.0	0.1, 1.0	
- Missing	11	12	23	
EQ-5D VAS				
- N	8	7	15	0.296 *[2]
- Mean +/- SD	64.1 +/-20.0	74.9 +/-18.4	69.1 +/-19.4	
- Median	64.0	80.0	70.0	
- p25, p75	45.0, 82.5	55.0, 90.0	50.0, 86.0	
- Min, Max	40.0, 90.0	50.0, 98.0	40.0, 98.0	
- Missing	11	12	23	

*[1] = Chi^2-test, two-sided, *[2] = U-test, two-sided

10.5.3.2 PP set

Table 10.5.9: EQ-5D month 6 (PP set)

	Placebo N=9	Dexamethasone N=7	Total N=16	p-value
Mobility				
- no problems in walking about	6 (100.0%)	2 (66.7%)	8 (88.9%)	0.134 *1
- some problems in walking about	0 (0.0%)	1 (33.3%)	1 (11.1%)	
- Missing	3	4	7	
Self-care				
- no problems with self-care	6 (100.0%)	2 (66.7%)	8 (88.9%)	0.134 *1
- some problems washing and dressing	0 (0.0%)	1 (33.3%)	1 (11.1%)	
- Missing	3	4	7	
Usual activities				
- no problems with performing usual activities	3 (50.0%)	1 (33.3%)	4 (44.4%)	0.325 *1
- some problems with permorming usual activities	3 (50.0%)	1 (33.3%)	4 (44.4%)	
- unable to perform usual activities	0 (0.0%)	1 (33.3%)	1 (11.1%)	
- Missing	3	4	7	
Pain/discomfort				
- no pain or discomfort	5 (83.3%)	1 (33.3%)	6 (66.7%)	0.134 *1
- moderate pain or discomfort	1 (16.7%)	2 (66.7%)	3 (33.3%)	
- Missing	3	4	7	
Anxiety/depression				
- not anxious or depressed	4 (66.7%)	1 (33.3%)	5 (55.6%)	0.343 *1
- moderately anxious or depressed	2 (33.3%)	2 (66.7%)	4 (44.4%)	
- Missing	3	4	7	
EQ-5D Index				
- N	6	3	9	0.044 *2
- Mean +/- SD	1.0 +/-0.0	0.8 +/-0.2	0.9 +/-0.2	
- Median	1.0	0.9	1.0	
- p25, p75	1.0, 1.0	0.5, 0.9	0.9, 1.0	
- Min, Max	0.9, 1.0	0.5, 0.9	0.5, 1.0	
- Missing	3	4	7	
EQ-5D VAS				
- N	6	3	9	0.193 *2
- Mean +/- SD	72.8 +/-14.5	57.0 +/-2.6	67.6 +/-14.0	
- Median	78.5	56.0	60.0	
- p25, p75	60.0, 85.0	55.0, 60.0	56.0, 80.0	
- Min, Max	50.0, 85.0	55.0, 60.0	50.0, 85.0	
- Missing	3	4	7	

*1 = Chi^2-test, two-sided, *2 = U-test, two-sided

Table 10.5.10: EQ-5D month 12 (PP set)

	Placebo N=9	Dexamethasone N=7	Total N=16	p-value
Mobility				
- no problems in walking about	4 (100.0%)	2 (100.0%)	6 (100.0%)	
- Missing	5	5	10	
Self-care				
- no problems with self-care	4 (100.0%)	2 (100.0%)	6 (100.0%)	
- Missing	5	5	10	
Usual activities				
- no problems with performing usual activities	2 (50.0%)	2 (100.0%)	4 (66.7%)	0.221 *[1]
- some problems with permorming usual activities	2 (50.0%)	0 (0.0%)	2 (33.3%)	
- Missing	5	5	10	
Pain/discomfort				
- no pain or discomfort	3 (75.0%)	1 (50.0%)	4 (66.7%)	0.540 *[1]
- moderate pain or discomfort	1 (25.0%)	1 (50.0%)	2 (33.3%)	
- Missing	5	5	10	
Anxiety/depression				
- not anxious or depressed	2 (50.0%)	2 (100.0%)	4 (66.7%)	0.472 *[1]
- moderately anxious or depressed	1 (25.0%)	0 (0.0%)	1 (16.7%)	
- extremely anxious or depressed	1 (25.0%)	0 (0.0%)	1 (16.7%)	
- Missing	5	5	10	
EQ-5D Index				
- N	4	2	6	1.000 *[2]
- Mean +/- SD	0.9 +/-0.3	0.9 +/-0.1	0.9 +/-0.2	
- Median	1.0	0.9	1.0	
- p25, p75	0.7, 1.0	0.9, 1.0	0.9, 1.0	
- Min, Max	0.5, 1.0	0.9, 1.0	0.5, 1.0	
- Missing	5	5	10	
EQ-5D VAS				
- N	4	2	6	0.159 *[2]
- Mean +/- SD	70.0 +/-21.6	94.0 +/-5.7	78.0 +/-21.0	
- Median	75.0	94.0	85.0	
- p25, p75	55.0, 85.0	90.0, 98.0	70.0, 90.0	
- Min, Max	40.0, 90.0	90.0, 98.0	40.0, 98.0	
- Missing	5	5	10	

*[1] = Chi^2-test, two-sided, *[2] = U-test, two-sided

10.5.4 Functional outcome (mRS)

10.5.4.1 FAS

Table 10.5.11: mRS at scheduled visit times (FAS)

	Placebo		Dexamethasone		All		p-value
Pre-encephalitis mRS*							
- 0	17	(89.5%)	14	(73.7%)	31	(81.6%)	0.0991
- 1	0	(0.0%)	4	(21.1%)	4	(10.5%)	
- 2	2	(10.5%)	1	(5.3%)	3	(7.9%)	
- 3	0	(0.0%)	0	(0.0%)	0	(0.0%)	
- 4	0	(0.0%)	0	(0.0%)	0	(0.0%)	
- 5	0	(0.0%)	0	(0.0%)	0	(0.0%)	
- 6	0	(0.0%)	0	(0.0%)	0	(0.0%)	
- Missing	0		0		0		
mRS – discharge*							
- 0	2	(11.1%)	2	(11.8%)	4	(11.4%)	0.4716
- 1	0	(0.0%)	2	(11.8%)	2	(5.7%)	
- 2	3	(16.7%)	4	(23.5%)	7	(20.0%)	
- 3	8	(44.4%)	4	(23.5%)	12	(34.3%)	
- 4	1	(5.6%)	0	(0.0%)	1	(2.9%)	
- 5	4	(22.2%)	5	(29.4%)	9	(25.7%)	
- 6	0	(0.0%)	0	(0.0%)	0	(0.0%)	
- Missing	1		2		3		
mRS - month 6							
- 0	0	(0.0%)	3	(21.4%)	3	(9.7%)	0.2106
- 1	4	(23.5%)	4	(28.6%)	8	(25.8%)	
- 2	8	(47.1%)	3	(21.4%)	11	(35.5%)	
- 3	2	(11.8%)	1	(7.1%)	3	(9.7%)	
- 4	0	(0.0%)	1	(7.1%)	1	(3.2%)	
- 5	3	(17.6%)	1	(7.1%)	4	(12.9%)	
- 6	0	(0.0%)	1	(7.1%)	1	(3.2%)	
- Missing	2		5		7		
mRS - month 12							
- 0	2	(12.5%)	5	(38.5%)	7	(24.1%)	0.2712
- 1	2	(12.5%)	2	(15.4%)	4	(13.8%)	
- 2	5	(31.3%)	0	(0.0%)	5	(17.2%)	
- 3	3	(18.8%)	3	(23.1%)	6	(20.7%)	
- 4	0	(0.0%)	0	(0.0%)	0	(0.0%)	
- 5	2	(12.5%)	2	(15.4%)	4	(13.8%)	
- 6	2	(12.5%)	1	(7.7%)	3	(10.3%)	
- Missing	3		6		9		

*No secondary endpoint

Table 10.5.12: Binary mRS at scheduled visit month 12 (FAS)

	Placebo		Dexamethasone		All		p-value
binary mRS month 12							
- <=2	9	(56.3%)	7	(53.8%)	16	(55.2%)	0.8970
- >2	7	(43.8%)	6	(46.2%)	13	(44.8%)	
- Missing	3		6		9		

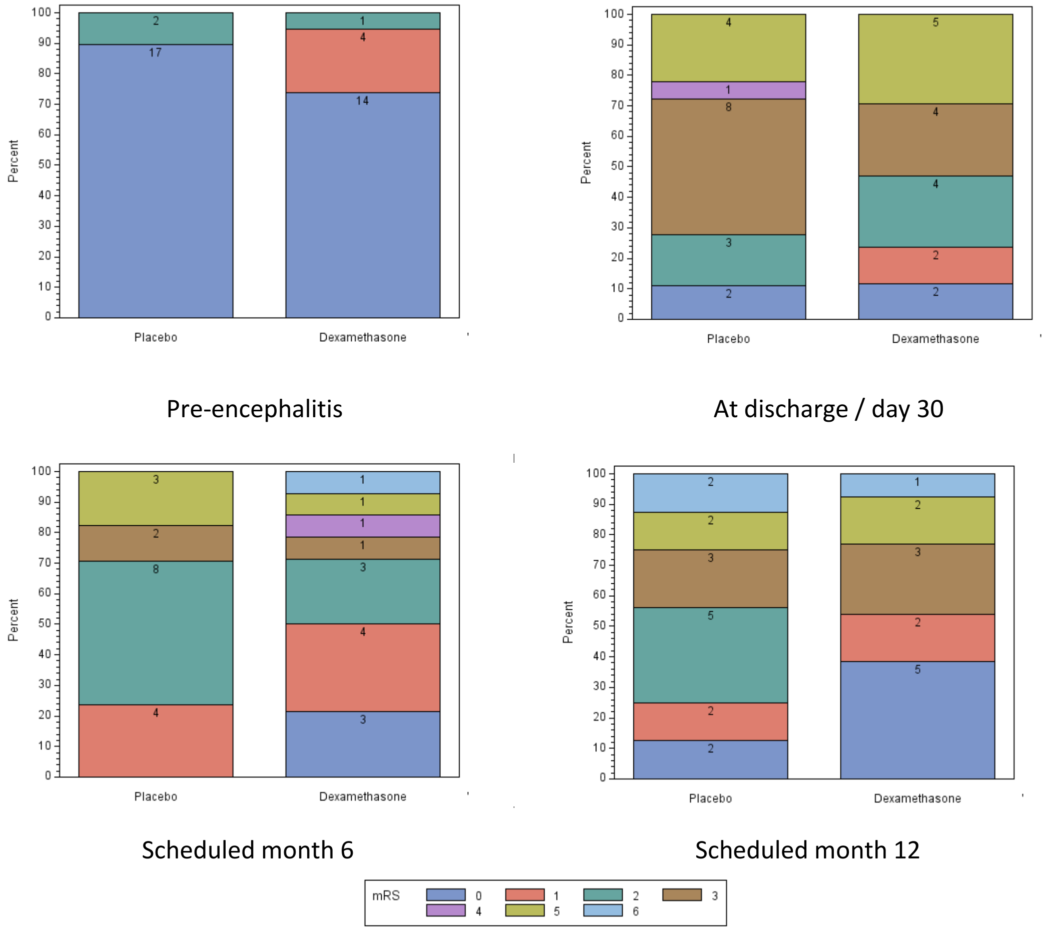

Figure 10.5.3: Stacked bar plots mRS (FAS).

10.5.4.2 PP set

Table 10.5.13: mRS at scheduled visit times (PP set)

	Placebo		Dexamethasone		All		p-value
Pre-encephalitis mRS*							
- 0	8	(88.9%)	6	(85.7%)	14	(87.5%)	0.3556
- 1	0	(0.0%)	1	(14.3%)	1	(6.3%)	
- 2	1	(11.1%)	0	(0.0%)	1	(6.3%)	
- 3	0	(0.0%)	0	(0.0%)	0	(0.0%)	
- 4	0	(0.0%)	0	(0.0%)	0	(0.0%)	
- 5	0	(0.0%)	0	(0.0%)	0	(0.0%)	
- 6	0	(0.0%)	0	(0.0%)	0	(0.0%)	
- Missing	0		0		0		
mRS - discharge							
- 0	2	(25.0%)	0	(0.0%)	2	(15.4%)	0.5577
- 1	0	(0.0%)	0	(0.0%)	0	(0.0%)	
- 2	1	(12.5%)	1	(20.0%)	2	(15.4%)	
- 3	3	(37.5%)	2	(40.0%)	5	(38.5%)	
- 4	1	(12.5%)	0	(0.0%)	1	(7.7%)	
- 5	1	(12.5%)	2	(40.0%)	3	(23.1%)	
- 6	0	(0.0%)	0	(0.0%)	0	(0.0%)	
- Missing	1		2		3		
	.						
mRS - month 6	.						
- 0	0	(0.0%)	1	(14.3%)	1	(6.3%)	0.3677
- 1	2	(22.2%)	2	(28.6%)	4	(25.0%)	
- 2	5	(55.6%)	1	(14.3%)	6	(37.5%)	
- 3	1	(11.1%)	1	(14.3%)	2	(12.5%)	
- 4	0	(0.0%)	1	(14.3%)	1	(6.3%)	
- 5	1	(11.1%)	0	(0.0%)	1	(6.3%)	
- 6	0	(0.0%)	1	(14.3%)	1	(6.3%)	
- Missing	0		0		0		
mRS - month 12							
- 0	1	(12.5%)	2	(28.6%)	3	(20.0%)	0.5897
- 1	2	(25.0%)	1	(14.3%)	3	(20.0%)	
- 2	2	(25.0%)	0	(0.0%)	2	(13.3%)	
- 3	2	(25.0%)	3	(42.9%)	5	(33.3%)	
- 4	0	(0.0%)	0	(0.0%)	0	(0.0%)	
- 5	0	(0.0%)	0	(0.0%)	0	(0.0%)	
- 6	1	(12.5%)	1	(14.3%)	2	(13.3%)	
- Missing	1		0		1		

*No secondary endpoint

Table 10.5.14: Binary mRS at scheduled visit times (PP set)

	Placebo		Dexamethasone		All		p-value
binary mRS month 12							
- <=2	5	(62.5%)	3	(42.9%)	8	(53.3%)	0.4468
- >2	3	(37.5%)	4	(57.1%)	7	(46.7%)	
- Missing	1		0		1		

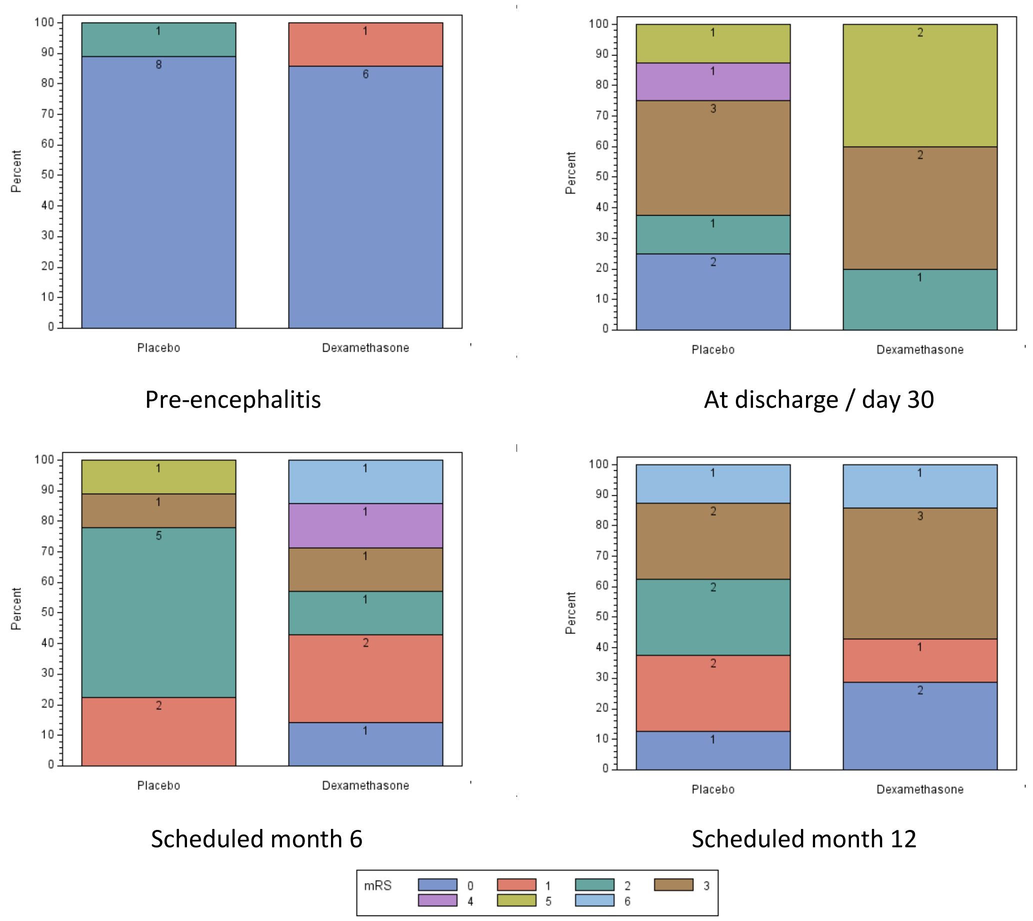

Figure 10.5.4: Stacked bar plots mRS (PP set).

10.5.5 Neuropsychological testing at month 6

Table 10.5.15: Available reasons for missing neuropsychological testing

Patient	Neuropsychologic examination
02-005	No month 6 visit done
07-102	No month 6 visit done
08-004	No month 6 visit done
08-009	No month 6 visit done
11-001	No month 6 visit done
12-033	no information available
12-093	No month 6 visit done
12-094	No month 6 visit done
13-002	unable to perform tasks
16-009	Patient died
16-019	Complex Figure, TMT: withrawal because of nausea
18-001	Complex Figure: figure of mini-mental test was shown instead of complex figure
19-007	unable to perform due to neurological deficit.
21-008	no information available
26-051	Complex Figure: refused due to dizziness

10.5.5.1 FAS

Table 10.5.16: Mini-Mental test (FAS)

	Placebo N=19	Dexamethasone N=19	Total N=38	p-value
Orientation				
- N	13	13	26	0.907 *2
- Mean +/- SD	9.3 +/-1.0	8.9 +/-1.9	9.1 +/-1.5	
- Median	10.0	10.0	10.0	
- p25, p75	9.0, 10.0	9.0, 10.0	9.0, 10.0	
- Min, Max	7.0, 10.0	4.0, 10.0	4.0, 10.0	
- Missing	6	6	12	
Immediate recall				
- N	13	13	26	0.356 *2
- Mean +/- SD	2.9 +/-0.3	3.0 +/-0.0	3.0 +/-0.2	
- Median	3.0	3.0	3.0	
- p25, p75	3.0, 3.0	3.0, 3.0	3.0, 3.0	
- Min, Max	2.0, 3.0	3.0, 3.0	2.0, 3.0	
- Missing	6	6	12	
Serial 7s total				
- N	13	13	26	0.287 *2
- Mean +/- SD	4.3 +/-0.9	4.6 +/-0.8	4.5 +/-0.8	
- Median	5.0	5.0	5.0	
- p25, p75	4.0, 5.0	5.0, 5.0	4.0, 5.0	
- Min, Max	3.0, 5.0	3.0, 5.0	3.0, 5.0	
- Missing	6	6	12	
Delayed verbal recall				
- N	13	13	26	0.398 *2
- Mean +/- SD	1.8 +/-1.4	1.3 +/-1.4	1.5 +/-1.4	
- Median	2.0	1.0	2.0	
- p25, p75	0.0, 3.0	0.0, 3.0	0.0, 3.0	
- Min, Max	0.0, 3.0	0.0, 3.0	0.0, 3.0	
- Missing	6	6	12	
Naming				
- N	13	13	26	0.263 *2
- Mean +/- SD	8.4 +/-1.0	8.7 +/-0.8	8.5 +/-0.9	
- Median	9.0	9.0	9.0	
- p25, p75	8.0, 9.0	9.0, 9.0	8.0, 9.0	
- Min, Max	6.0, 9.0	7.0, 9.0	6.0, 9.0	
- Missing	6	6	12	
Total score				
- N	13	13	26	0.775 *2
- Mean +/- SD	26.7 +/-2.6	26.5 +/-3.7	26.6 +/-3.1	
- Median	27.0	27.0	27.0	
- p25, p75	25.0, 29.0	26.0, 29.0	25.0, 29.0	
- Min, Max	22.0, 30.0	19.0, 30.0	19.0, 30.0	
- Missing	6	6	12	

*2 = U-test, two-sided

Table 10.5.17: Complex Figure (FAS)

	Placebo N=19	Dexamethasone N=19	Total N=38	p-value
Copy				
- N	13	11	24	0.221 *2
- Mean +/- SD	32.0 +/-5.7	30.5 +/-5.2	31.3 +/-5.4	
- Median	34.0	32.0	33.0	
- p25, p75	31.0, 35.0	27.0, 33.0	30.8, 35.0	
- Min, Max	15.0, 36.0	17.5, 36.0	15.0, 36.0	
- Missing	6	8	14	
Immediate recall				
- N	11	11	22	0.307 *2
- Mean +/- SD	13.9 +/-6.5	10.6 +/-6.0	12.3 +/-6.3	
- Median	11.5	10.0	11.5	
- p25, p75	8.5, 19.0	6.5, 15.0	8.0, 15.5	
- Min, Max	8.0, 29.5	0.0, 21.0	0.0, 29.5	
- Missing	8	8	16	
Delayed recall				
- N	11	12	23	0.975 *2
- Mean +/- SD	12.4 +/-7.1	11.3 +/-7.2	11.8 +/-7.0	
- Median	10.5	10.8	10.5	
- p25, p75	8.0, 18.0	6.8, 17.8	8.0, 18.0	
- Min, Max	4.0, 28.5	0.0, 20.5	0.0, 28.5	
- Missing	8	7	15	
Sum of copy/immediate recall				
- N	13	11	24	0.602 *2
- Mean +/- SD	43.8 +/-11.3	41.1 +/-9.9	42.6 +/-10.5	
- Median	43.0	42.0	42.5	
- p25, p75	40.5, 50.5	32.5, 48.0	35.0, 49.0	
- Min, Max	23.0, 64.5	25.5, 57.0	23.0, 64.5	
- Missing	6	8	14	

*2 = U-test, two-sided

Table 10.5.18: Auditory Verbal Learning Test (FAS)

	Placebo N=19	Dexamethasone N=19	Total N=38	p-value
Learning trial (Dg 1-5)				
- N	12	13	25	0.103 *2
- Mean +/- SD	42.1 +/-16.9	30.6 +/-12.3	36.1 +/-15.5	
- Median	43.0	29.0	31.0	
- p25, p75	27.5, 55.5	27.0, 35.0	27.0, 45.0	
- Min, Max	17.0, 69.0	10.0, 57.0	10.0, 69.0	
- Missing	7	6	13	
Interference trial (Dg 6)				
- N	12	13	25	0.163 *2
- Mean +/- SD	7.4 +/-5.6	4.2 +/-4.2	5.8 +/-5.1	
- Median	7.0	3.0	4.0	
- p25, p75	3.0, 12.0	1.0, 6.0	2.0, 10.0	
- Min, Max	0.0, 16.0	0.0, 13.0	0.0, 16.0	
- Missing	7	6	13	
Delayed recall (Dg 7)				
- N	13	13	26	0.244 *2
- Mean +/- SD	6.1 +/-4.7	3.8 +/-4.3	5.0 +/-4.5	
- Median	6.0	2.0	4.0	
- p25, p75	3.0, 10.0	1.0, 5.0	1.0, 8.0	
- Min, Max	0.0, 15.0	0.0, 13.0	0.0, 15.0	
- Missing	6	6	12	

*2 = U-test, two-sided

Table 10.5.19: Digit Span (FAS)

	Placebo N=19	Dexamethasone N=19	Total N=38	p-value
Digit Span forward Total				
- N	13	12	25	0.051 *2
- Mean +/- SD	5.8 +/-2.4	8.0 +/-2.3	6.9 +/-2.5	
- Median	6.0	8.0	7.0	
- p25, p75	4.0, 7.0	6.0, 9.5	6.0, 8.0	
- Min, Max	2.0, 10.0	5.0, 13.0	2.0, 13.0	
- Missing	6	7	13	
Digit Span forward Range				
- N	13	12	25	0.074 *2
- Mean +/- SD	5.0 +/-1.7	5.9 +/-0.8	5.4 +/-1.4	
- Median	5.0	6.0	5.5	
- p25, p75	4.5, 6.0	5.3, 6.5	5.0, 6.0	
- Min, Max	1.0, 8.0	4.5, 7.0	1.0, 8.0	
- Missing	6	7	13	
Digit Span backward Total				
- N	13	13	26	0.585 *2
- Mean +/- SD	5.2 +/-2.0	5.6 +/-1.9	5.4 +/-1.9	
- Median	5.0	6.0	5.5	
- p25, p75	4.0, 7.0	4.0, 7.0	4.0, 7.0	
- Min, Max	2.0, 9.0	3.0, 9.0	2.0, 9.0	
- Missing	6	6	12	
Digit Span backward Range				
- N	13	13	26	0.299 *2
- Mean +/- SD	3.7 +/-1.2	4.2 +/-1.2	3.9 +/-1.2	
- Median	4.0	4.0	4.0	
- p25, p75	3.0, 4.5	4.0, 5.0	3.0, 4.5	
- Min, Max	2.0, 5.5	1.5, 6.0	1.5, 6.0	
- Missing	6	6	12	

*2 = U-test, two-sided

Table 10.5.20: Trail Making Test (FAS)

	Placebo N=19	Dexamethasone N=19	Total N=38	p-value
Time Test A (sec.)				
- N	13	13	26	0.101 *2
- Mean +/- SD	45.5 +/-45.3	60.0 +/-40.4	52.7 +/-42.7	
- Median	32.0	52.0	40.5	
- p25, p75	28.0, 45.0	36.0, 59.0	32.0, 54.0	
- Min, Max	8.0, 189.0	24.0, 175.0	8.0, 189.0	
- Missing	6	6	12	
Time Test B (sec.)				
- N	12	13	25	0.314 *2
- Mean +/- SD	155.3 +/-198.4	160.0 +/-95.0	157.8 +/-150.2	
- Median	80.0	135.0	119.0	
- p25, p75	57.5, 147.0	91.0, 233.0	61.0, 200.0	
- Min, Max	25.0, 740.0	51.0, 300.0	25.0, 740.0	
- Missing	7	6	13	

*2 = U-test, two-sided

Table 10.5.21: Word Fluency (FAS)

	Placebo N=19	Dexamethasone N=19	Total N=38	p-value
Sum of animal items				
- N	13	13	26	0.487 *2
- Mean +/- SD	16.3 +/-5.9	13.9 +/-4.3	15.1 +/-5.2	
- Median	14.0	15.0	14.5	
- p25, p75	13.0, 20.0	12.0, 16.0	12.0, 17.0	
- Min, Max	7.0, 27.0	6.0, 21.0	6.0, 27.0	
- Missing	6	6	12	
Sum of food items				
- N	13	13	26	0.521 *2
- Mean +/- SD	16.2 +/-6.3	14.8 +/-6.6	15.5 +/-6.4	
- Median	18.0	15.0	15.0	
- p25, p75	12.0, 22.0	12.0, 17.0	12.0, 20.0	
- Min, Max	6.0, 24.0	2.0, 30.0	2.0, 30.0	
- Missing	6	6	12	
WF Total				
- N	13	13	26	0.572 *2
- Mean +/- SD	32.5 +/-10.6	28.8 +/-10.4	30.7 +/-10.5	
- Median	28.0	30.0	29.5	
- p25, p75	25.0, 43.0	24.0, 33.0	24.0, 38.0	
- Min, Max	19.0, 48.0	9.0, 51.0	9.0, 51.0	
- Missing	6	6	12	

*2 = U-test, two-sided

Table 10.5.22: Hospital Anxiety and Depression Scale HADS-D (FAS)

	Placebo N=19	Dexamethasone N=19	Total N=38	p-value
HADS-D Anxiety Score				
- N	13	13	26	0.918 *2
- Mean +/- SD	7.6 +/-4.5	7.4 +/-4.0	7.5 +/-4.2	
- Median	8.0	6.0	7.0	
- p25, p75	4.0, 11.0	5.0, 10.0	4.0, 10.0	
- Min, Max	0.0, 14.0	2.0, 15.0	0.0, 15.0	
- Missing	6	6	12	
HADS-D Depression Score				
- N	13	13	26	0.699 *2
- Mean +/- SD	7.5 +/-5.2	6.8 +/-4.5	7.2 +/-4.7	
- Median	9.0	7.0	7.0	
- p25, p75	3.0, 12.0	3.0, 8.0	3.0, 11.0	
- Min, Max	0.0, 16.0	1.0, 17.0	0.0, 17.0	
- Missing	6	6	12	

*2 = U-test, two-sided

Table 10.5.23: Categorized Hospital Anxiety and Depression Scale HADS-D (FAS)

	Placebo N=19	Dexamethasone N=19	Total N=38	p-value
HADS-D Anxiety Score				
- negative (0-7)	6 (46.2%)	7 (53.8%)	13 (50.0%)	0.642 *1
- netural (8-10)	3 (23.1%)	4 (30.8%)	7 (26.9%)	
- positive (>= 11)	4 (30.8%)	2 (15.4%)	6 (23.1%)	
- Missing	6	6	12	
HADS-D Depression Score				
- negative (0-7)	6 (46.2%)	8 (61.5%)	14 (53.8%)	0.412 *1
- netural (8-10)	2 (15.4%)	3 (23.1%)	5 (19.2%)	
- positive (>= 11)	5 (38.5%)	2 (15.4%)	7 (26.9%)	
- Missing	6	6	12	

*1 = Chi^2-test, two-sided

10.5.5.2 PP set

Table 10.5.24: Mini-Mental test (PP set)

	Placebo N=9	Dexamethasone N=7	Total N=16	p-value
Orientation				
- N	6	6	12	0.214 *2
- Mean +/- SD	9.8 +/-0.4	8.8 +/-1.6	9.3 +/-1.2	
- Median	10.0	9.5	10.0	
- p25, p75	10.0, 10.0	8.0, 10.0	9.0, 10.0	
- Min, Max	9.0, 10.0	6.0, 10.0	6.0, 10.0	
- Missing	3	1	4	
Immediate recall				
- N	6	6	12	1.000 *2
- Mean +/- SD	3.0 +/-0.0	3.0 +/-0.0	3.0 +/-0.0	
- Median	3.0	3.0	3.0	
- p25, p75	3.0, 3.0	3.0, 3.0	3.0, 3.0	
- Min, Max	3.0, 3.0	3.0, 3.0	3.0, 3.0	
- Missing	3	1	4	
Serial 7s total				
- N	6	6	12	0.336 *2
- Mean +/- SD	4.2 +/-1.0	4.7 +/-0.8	4.4 +/-0.9	
- Median	4.5	5.0	5.0	
- p25, p75	3.0, 5.0	5.0, 5.0	3.5, 5.0	
- Min, Max	3.0, 5.0	3.0, 5.0	3.0, 5.0	
- Missing	3	1	4	
Delayed verbal recall				
- N	6	6	12	0.256 *2
- Mean +/- SD	1.8 +/-1.5	0.8 +/-1.3	1.3 +/-1.4	
- Median	2.5	0.0	1.0	
- p25, p75	0.0, 3.0	0.0, 2.0	0.0, 3.0	
- Min, Max	0.0, 3.0	0.0, 3.0	0.0, 3.0	
- Missing	3	1	4	
Naming				
- N	6	6	12	1.000 *2
- Mean +/- SD	8.8 +/-0.4	8.7 +/-0.8	8.8 +/-0.6	
- Median	9.0	9.0	9.0	
- p25, p75	9.0, 9.0	9.0, 9.0	9.0, 9.0	
- Min, Max	8.0, 9.0	7.0, 9.0	7.0, 9.0	
- Missing	3	1	4	
Total score				
- N	6	6	12	0.515 *2
- Mean +/- SD	27.7 +/-2.0	26.0 +/-3.6	26.8 +/-2.9	
- Median	28.0	27.0	27.0	
- p25, p75	26.0, 29.0	26.0, 28.0	26.0, 29.0	
- Min, Max	25.0, 30.0	19.0, 29.0	19.0, 30.0	
- Missing	3	1	4	

*2 = U-test, two-sided

Table 10.5.25: Complex Figure (PP set)

	Placebo N=9	Dexamethasone N=7	Total N=16	p-value
Copy				
- N	6	5	11	0.032 *2
- Mean +/- SD	34.8 +/-1.5	32.3 +/-0.7	33.7 +/-1.7	
- Median	35.0	32.0	33.0	
- p25, p75	35.0, 36.0	32.0, 33.0	32.0, 35.0	
- Min, Max	32.0, 36.0	31.5, 33.0	31.5, 36.0	
- Missing	3	2	5	
Immediate recall				
- N	6	5	11	0.411 *2
- Mean +/- SD	15.5 +/-8.0	10.6 +/-4.3	13.3 +/-6.8	
- Median	14.0	10.0	12.5	
- p25, p75	8.5, 19.0	9.0, 14.5	8.5, 15.5	
- Min, Max	8.0, 29.5	4.5, 15.0	4.5, 29.5	
- Missing	3	2	5	
Delayed recall				
- N	6	6	12	0.936 *2
- Mean +/- SD	13.9 +/-8.8	11.5 +/-7.8	12.7 +/-8.0	
- Median	11.8	13.3	12.0	
- p25, p75	8.0, 19.5	5.0, 17.0	6.5, 18.3	
- Min, Max	4.0, 28.5	0.0, 20.5	0.0, 28.5	
- Missing	3	1	4	
Sum of copy/immediate recall				
- N	6	5	11	0.121 *2
- Mean +/- SD	50.3 +/-8.7	42.9 +/-5.0	47.0 +/-7.9	
- Median	49.5	42.0	47.5	
- p25, p75	43.0, 55.0	41.0, 47.5	41.0, 50.5	
- Min, Max	40.5, 64.5	36.0, 48.0	36.0, 64.5	
- Missing	3	2	5	

*2 = U-test, two-sided

Table 10.5.26: Auditory Verbal Learning Test (PP set)

	Placebo N=9	Dexamethasone N=7	Total N=16	p-value
Learning trial (Dg 1-5)				
- N	6	6	12	0.013 *2
- Mean +/- SD	46.0 +/-11.9	25.8 +/-8.7	35.9 +/-14.5	
- Median	46.5	28.0	33.0	
- p25, p75	36.0, 55.0	23.0, 31.0	28.0, 46.5	
- Min, Max	30.0, 62.0	10.0, 35.0	10.0, 62.0	
- Missing	3	1	4	
Interference trial (Dg 6)				
- N	6	6	12	0.053 *2
- Mean +/- SD	7.7 +/-4.1	2.5 +/-2.5	5.1 +/-4.2	
- Median	7.5	2.0	4.5	
- p25, p75	4.0, 12.0	0.0, 5.0	2.0, 8.0	
- Min, Max	3.0, 12.0	0.0, 6.0	0.0, 12.0	
- Missing	3	1	4	
Delayed recall (Dg 7)				
- N	6	6	12	0.012 *2
- Mean +/- SD	7.2 +/-3.2	2.2 +/-1.6	4.7 +/-3.6	
- Median	7.5	2.0	3.5	
- p25, p75	4.0, 10.0	2.0, 2.0	2.0, 7.5	
- Min, Max	3.0, 11.0	0.0, 5.0	0.0, 11.0	
- Missing	3	1	4	

*2 = U-test, two-sided

Table 10.5.27: Digit Span (PP set)

	Placebo N=9	Dexamethasone N=7	Total N=16	p-value
Digit Span forward Total				
- N	6	6	12	0.346 *2
- Mean +/- SD	5.5 +/-1.4	7.0 +/-2.0	6.3 +/-1.8	
- Median	6.0	6.0	6.0	
- p25, p75	5.0, 6.0	6.0, 9.0	5.5, 6.5	
- Min, Max	3.0, 7.0	5.0, 10.0	3.0, 10.0	
- Missing	3	1	4	
Digit Span forward Range				
- N	6	6	12	0.285 *2
- Mean +/- SD	5.0 +/-0.4	5.7 +/-1.0	5.3 +/-0.8	
- Median	5.0	5.5	5.0	
- p25, p75	4.5, 5.5	5.0, 6.5	4.8, 5.8	
- Min, Max	4.5, 5.5	4.5, 7.0	4.5, 7.0	
- Missing	3	1	4	
Digit Span backward Total				
- N	6	6	12	0.685 *2
- Mean +/- SD	5.5 +/-2.5	6.0 +/-1.8	5.8 +/-2.1	
- Median	5.5	5.5	5.5	
- p25, p75	3.0, 7.0	5.0, 7.0	4.0, 7.0	
- Min, Max	3.0, 9.0	4.0, 9.0	3.0, 9.0	
- Missing	3	1	4	
Digit Span backward Range				
- N	6	6	12	0.685 *2
- Mean +/- SD	4.1 +/-1.3	4.3 +/-0.9	4.2 +/-1.1	
- Median	4.0	4.0	4.0	
- p25, p75	3.0, 5.5	4.0, 4.5	3.5, 5.0	
- Min, Max	2.5, 5.5	3.5, 6.0	2.5, 6.0	
- Missing	3	1	4	

*2 = U-test, two-sided

Table 10.5.28: Trail Making test (PP set)

	Placebo N=9	Dexamethasone N=7	Total N=16	p-value
Time Test A (sec.)				
- N	6	6	12	0.468 *2
- Mean +/- SD	38.0 +/-11.8	46.8 +/-20.2	42.4 +/-16.4	
- Median	32.0	45.5	35.5	
- p25, p75	30.0, 51.0	32.0, 53.0	31.0, 52.5	
- Min, Max	28.0, 55.0	24.0, 81.0	24.0, 81.0	
- Missing	3	1	4	
Time Test B (sec.)				
- N	6	6	12	0.471 *2
- Mean +/- SD	105.5 +/-39.3	157.2 +/-91.2	131.3 +/-72.2	
- Median	104.5	129.0	121.5	
- p25, p75	75.0, 144.0	91.0, 233.0	80.0, 147.0	
- Min, Max	55.0, 150.0	61.0, 300.0	55.0, 300.0	
- Missing	3	1	4	

*2 = U-test, two-sided

Table 10.5.29: Word Fluency (PP set)

	Placebo N=9	Dexamethasone N=7	Total N=16	p-value
Sum of animal items				
- N	6	6	12	0.520 *2
- Mean +/- SD	16.5 +/-5.1	13.2 +/-3.9	14.8 +/-4.6	
- Median	14.5	14.0	14.5	
- p25, p75	14.0, 20.0	11.0, 16.0	11.5, 16.5	
- Min, Max	11.0, 25.0	7.0, 17.0	7.0, 25.0	
- Missing	3	1	4	
Sum of food items				
- N	6	6	12	0.198 *2
- Mean +/- SD	17.0 +/-5.7	12.2 +/-5.5	14.6 +/-5.9	
- Median	16.0	12.5	13.5	
- p25, p75	13.0, 23.0	12.0, 17.0	12.0, 17.5	
- Min, Max	10.0, 24.0	2.0, 17.0	2.0, 24.0	
- Missing	3	1	4	
WF Total				
- N	6	6	12	0.295 *2
- Mean +/- SD	33.5 +/-9.1	25.3 +/-9.1	29.4 +/-9.7	
- Median	33.0	27.0	29.0	
- p25, p75	28.0, 43.0	23.0, 33.0	23.5, 35.5	
- Min, Max	21.0, 43.0	9.0, 33.0	9.0, 43.0	
- Missing	3	1	4	

*2 = U-test, two-sided

Table 10.5.30: Hospital Anxiety and Depression Scale HADS-D (PP set)

	Placebo N=9	Dexamethasone N=7	Total N=16	p-value
HADS-D Anxiety Score				
- N	6	6	12	0.747 *[2]
- Mean +/- SD	7.2 +/-4.2	6.2 +/-3.8	6.7 +/-3.8	
- Median	7.0	5.5	6.0	
- p25, p75	4.0, 9.0	3.0, 9.0	3.5, 9.0	
- Min, Max	2.0, 14.0	2.0, 12.0	2.0, 14.0	
- Missing	3	1	4	
HADS-D Depression Score				
- N	6	6	12	0.686 *[2]
- Mean +/- SD	7.3 +/-6.0	5.5 +/-3.1	6.4 +/-4.6	
- Median	6.0	6.0	6.0	
- p25, p75	3.0, 12.0	3.0, 8.0	3.0, 9.0	
- Min, Max	1.0, 16.0	1.0, 9.0	1.0, 16.0	
- Missing	3	1	4	

*[2] = U-test, two-sided

Table 10.5.31: Categorized Hospital Anxiety and Depression Scale HADS-D (PP set)

	Placebo N=9	Dexamethasone N=7	Total N=16	p-value
HADS-D Anxiety Score				
- negative (0-7)	3 (50.0%)	4 (66.7%)	7 (58.3%)	0.788 *[1]
- netural (8-10)	2 (33.3%)	1 (16.7%)	3 (25.0%)	
- positive (>= 11)	1 (16.7%)	1 (16.7%)	2 (16.7%)	
- Missing	3	1	4	
HADS-D Depression Score				
- negative (0-7)	3 (50.0%)	4 (66.7%)	7 (58.3%)	0.290 *[1]
- netural (8-10)	1 (16.7%)	2 (33.3%)	3 (25.0%)	
- positive (>= 11)	2 (33.3%)	0 (0.0%)	2 (16.7%)	
- Missing	3	1	4	

*[1] = Chi^2-test, two-sided

10.5.6 Barthel index

10.5.6.1 FAS

Table 10.5.32: Barthel index total (FAS)

	Placebo N=19	Dexamethasone N=19	Total N=38	p-value
Discharge/Day 30 Barthel Index Total				
- N	18	16	34	0.874 *2
- Mean +/- SD	65.8 +/-37.4	68.8 +/-40.3	67.2 +/-38.2	
- Median	77.5	90.0	85.0	
- p25, p75	40.0, 100.0	37.5, 100.0	40.0, 100.0	
- Min, Max	5.0, 100.0	0.0, 100.0	0.0, 100.0	
- Missing	1	3	4	
6 Months Barthel Index Total				
- N	17	12	29	0.646 *2
- Mean +/- SD	86.2 +/-31.9	93.8 +/-13.0	89.3 +/-25.8	
- Median	100.0	100.0	100.0	
- p25, p75	95.0, 100.0	95.0, 100.0	95.0, 100.0	
- Min, Max	0.0, 100.0	60.0, 100.0	0.0, 100.0	
- Missing	2	7	9	
12 Months Barthel Index Total				
- N	14	12	26	0.737 *2
- Mean +/- SD	91.4 +/-26.6	92.5 +/-24.4	91.9 +/-25.1	
- Median	100.0	100.0	100.0	
- p25, p75	100.0, 100.0	100.0, 100.0	100.0, 100.0	
- Min, Max	0.0, 100.0	15.0, 100.0	0.0, 100.0	
- Missing	5	7	12	

*2 = U-test, two-sided

Table 10.5.33: Barthel index items at discharge/day 30 (FAS)

	Placebo N=19	Dexamethasone N=19	Total N=38	p-value
Feeding				
- 0-unable	3 (16.7%)	3 (18.8%)	6 (17.6%)	0.683 *[1]
- 5-needs help	7 (38.9%)	4 (25.0%)	11 (32.4%)	
- 10-independent	8 (44.4%)	9 (56.3%)	17 (50.0%)	
- Missing	1	3	4	
Bathing				
- 0-dependent	8 (44.4%)	7 (43.8%)	15 (44.1%)	0.968 *[1]
- 5-independent	10 (55.6%)	9 (56.3%)	19 (55.9%)	
- Missing	1	3	4	
Grooming				
- 0-needs to help	9 (50.0%)	7 (43.8%)	16 (47.1%)	0.716 *[1]
- 5-independent	9 (50.0%)	9 (56.3%)	18 (52.9%)	
- Missing	1	3	4	
Dressing				
- 0-dependent	5 (27.8%)	4 (25.0%)	9 (26.5%)	0.536 *[1]
- 5-needs help	6 (33.3%)	3 (18.8%)	9 (26.5%)	
- 10-independent	7 (38.9%)	9 (56.3%)	16 (47.1%)	
- Missing	1	3	4	
Bowels				
- 0-incontinent	3 (16.7%)	4 (25.0%)	7 (20.6%)	0.219 *[1]
- 5-occasional accident	3 (16.7%)	0 (0.0%)	3 (8.8%)	
- 10-continent	12 (66.7%)	12 (75.0%)	24 (70.6%)	
- Missing	1	3	4	
Bladder				
- 0-incontinent/catheterized	5 (27.8%)	4 (25.0%)	9 (26.5%)	0.360 *[1]
- 5-occasional accident	2 (11.1%)	0 (0.0%)	2 (5.9%)	
- 10-continent	11 (61.1%)	12 (75.0%)	23 (67.6%)	
- Missing	1	3	4	
Toilet Use				
- 0-dependent	5 (27.8%)	4 (25.0%)	9 (26.5%)	0.699 *[1]
- 5-needs some help	4 (22.2%)	2 (12.5%)	6 (17.6%)	
- 10-independent	9 (50.0%)	10 (62.5%)	19 (55.9%)	
- Missing	1	3	4	
Bladder				
- 0-incontinent/catheterized	5 (27.8%)	4 (25.0%)	9 (26.5%)	0.360 *[1]
- 5-occasional accident	2 (11.1%)	0 (0.0%)	2 (5.9%)	
- 10-continent	11 (61.1%)	12 (75.0%)	23 (67.6%)	
- Missing	1	3	4	
Toilet Use				
- 0-dependent	5 (27.8%)	4 (25.0%)	9 (26.5%)	0.699 *[1]
- 5-needs some help	4 (22.2%)	2 (12.5%)	6 (17.6%)	
- 10-independent	9 (50.0%)	10 (62.5%)	19 (55.9%)	
- Missing	1	3	4	

	Placebo N=19	Dexamethasone N=19	Total N=38	p-value
Transfer (bed to chair and back)				
- 0-unable	0 (0.0%)	2 (12.5%)	2 (5.9%)	0.335 *1
- 5-major help	5 (27.8%)	3 (18.8%)	8 (23.5%)	
- 10-minor help	3 (16.7%)	1 (6.3%)	4 (11.8%)	
- 15-independent	10 (55.6%)	10 (62.5%)	20 (58.8%)	
- Missing	1	3	4	
Mobility (on level surfaces)				
- 0-immobile	4 (22.2%)	4 (25.0%)	8 (23.5%)	0.759 *1
- 10-walks with help	4 (22.2%)	2 (12.5%)	6 (17.6%)	
- 15-independent	10 (55.6%)	10 (62.5%)	20 (58.8%)	
- Missing	1	3	4	
Stairs				
- 0-unable	4 (22.2%)	5 (31.3%)	9 (26.5%)	0.526 *1
- 5-needs help	5 (27.8%)	2 (12.5%)	7 (20.6%)	
- 10-independent	9 (50.0%)	9 (56.3%)	18 (52.9%)	
- Missing	1	3	4	

*1 = Chi^2-test, two-sided

Table 10.5.34: Barthel index items at month 6 (FAS)

	Placebo N=19	Dexamethasone N=19	Total N=38	p-value
Feeding				
- 0-unable	2 (11.8%)	0 (0.0%)	2 (6.7%)	0.438 *1
- 5-needs help	1 (5.9%)	1 (7.7%)	2 (6.7%)	
- 10-independent	14 (82.4%)	12 (92.3%)	26 (86.7%)	
- Missing	2	6	8	
Bathing				
- 0-dependent	3 (17.6%)	2 (15.4%)	5 (16.7%)	0.869 *1
- 5-independent	14 (82.4%)	11 (84.6%)	25 (83.3%)	
- Missing	2	6	8	
Grooming				
- 0-needs to help	2 (11.8%)	3 (23.1%)	5 (16.7%)	0.410 *1
- 5-independent	15 (88.2%)	10 (76.9%)	25 (83.3%)	
- Missing	2	6	8	
Dressing				
- 0-dependent	2 (11.8%)	0 (0.0%)	2 (6.7%)	0.392 *1
- 5-needs help	2 (11.8%)	1 (7.7%)	3 (10.0%)	
- 10-independent	13 (76.5%)	12 (92.3%)	25 (83.3%)	
- Missing	2	6	8	
Bowels				
- 0-incontinent	2 (11.8%)	0 (0.0%)	2 (6.7%)	0.240 *1
- 5-occasional accident	0 (0.0%)	1 (7.7%)	1 (3.3%)	
- 10-continent	15 (88.2%)	12 (92.3%)	27 (90.0%)	
- Missing	2	6	8	

	Placebo<br>N=19	Dexamethasone<br>N=19	Total<br>N=38	p-value
Bladder				
- 0-incontinent/catheterized	3 (17.6%)	0 (0.0%)	3 (10.0%)	0.158 *1
- 5-occasional accident	0 (0.0%)	1 (7.7%)	1 (3.3%)	
- 10-continent	14 (82.4%)	12 (92.3%)	26 (86.7%)	
- Missing	2	6	8	
Toilet Use				
- 0-dependent	2 (11.8%)	0 (0.0%)	2 (6.7%)	0.125 *1
- 5-needs some help	0 (0.0%)	2 (15.4%)	2 (6.7%)	
- 10-independent	15 (88.2%)	11 (84.6%)	26 (86.7%)	
- Missing	2	6	8	
Bladder				
- 0-incontinent/catheterized	3 (17.6%)	0 (0.0%)	3 (10.0%)	0.158 *1
- 5-occasional accident	0 (0.0%)	1 (7.7%)	1 (3.3%)	
- 10-continent	14 (82.4%)	12 (92.3%)	26 (86.7%)	
- Missing	2	6	8	
Toilet Use				
- 0-dependent	2 (11.8%)	0 (0.0%)	2 (6.7%)	0.125 *1
- 5-needs some help	0 (0.0%)	2 (15.4%)	2 (6.7%)	
- 10-independent	15 (88.2%)	11 (84.6%)	26 (86.7%)	
- Missing	2	6	8	
Transfer (bed to chair and back)				
- 0-unable	1 (5.9%)	0 (0.0%)	1 (3.3%)	0.245 *1
- 5-major help	1 (5.9%)	0 (0.0%)	1 (3.3%)	
- 10-minor help	0 (0.0%)	2 (15.4%)	2 (6.7%)	
- 15-independent	15 (88.2%)	11 (84.6%)	26 (86.7%)	
- Missing	2	6	8	
Mobility (on level surfaces)				
- 0-immobile	2 (11.8%)	0 (0.0%)	2 (6.7%)	0.438 *1
- 10-walks with help	1 (5.9%)	1 (7.7%)	2 (6.7%)	
- 15-independent	14 (82.4%)	12 (92.3%)	26 (86.7%)	
- Missing	2	6	8	
Stairs				
- 0-unable	2 (11.8%)	1 (8.3%)	3 (10.3%)	0.652 *1
- 5-needs help	1 (5.9%)	0 (0.0%)	1 (3.4%)	
- 10-independent	14 (82.4%)	11 (91.7%)	25 (86.2%)	
- Missing	2	7	9	

*1 = Chi^2-test, two-sided

Table 10.5.35: Barthel index items at month 12 (FAS)

	Placebo N=19	Dexamethasone N=19	Total N=38	p-value
Feeding				
- 0-unable	1 (7.1%)	0 (0.0%)	1 (3.8%)	0.363 *[1]
- 5-needs help	0 (0.0%)	1 (8.3%)	1 (3.8%)	
- 10-independent	13 (92.9%)	11 (91.7%)	24 (92.3%)	
- Missing	5	7	12	
Bathing				
- 0-dependent	1 (7.1%)	1 (8.3%)	2 (7.7%)	0.910 *[1]
- 5-independent	13 (92.9%)	11 (91.7%)	24 (92.3%)	
- Missing	5	7	12	
Grooming				
- 0-needs to help	1 (7.1%)	2 (16.7%)	3 (11.5%)	0.449 *[1]
- 5-independent	13 (92.9%)	10 (83.3%)	23 (88.5%)	
- Missing	5	7	12	
Dressing				
- 0-dependent	1 (7.1%)	1 (8.3%)	2 (7.7%)	0.910 *[1]
- 10-independent	13 (92.9%)	11 (91.7%)	24 (92.3%)	
- Missing	5	7	12	
Bowels				
- 0-incontinent	1 (7.1%)	0 (0.0%)	1 (3.8%)	0.363 *[1]
- 5-occasional accident	0 (0.0%)	1 (8.3%)	1 (3.8%)	
- 10-continent	13 (92.9%)	11 (91.7%)	24 (92.3%)	
- Missing	5	7	12	
Bladder				
- 0-incontinent/catheterized	2 (14.3%)	1 (8.3%)	3 (11.5%)	0.636 *[1]
- 10-continent	12 (85.7%)	11 (91.7%)	23 (88.5%)	
- Missing	5	7	12	
Toilet Use				
- 0-dependent	1 (7.1%)	1 (8.3%)	2 (7.7%)	0.910 *[1]
- 10-independent	13 (92.9%)	11 (91.7%)	24 (92.3%)	
- Missing	5	7	12	
Bladder				
- 0-incontinent/catheterized	2 (14.3%)	1 (8.3%)	3 (11.5%)	0.636 *[1]
- 10-continent	12 (85.7%)	11 (91.7%)	23 (88.5%)	
- Missing	5	7	12	
Toilet Use				
- 0-dependent	1 (7.1%)	1 (8.3%)	2 (7.7%)	0.910 *[1]
- 10-independent	13 (92.9%)	11 (91.7%)	24 (92.3%)	
- Missing	5	7	12	

	Placebo N=19	Dexamethasone N=19	Total N=38	p-value
Transfer (bed to chair and back)				
- 0-unable	1 (7.1%)	0 (0.0%)	1 (3.8%)	0.406 *1
- 5-major help	0 (0.0%)	1 (8.3%)	1 (3.8%)	
- 10-minor help	1 (7.1%)	0 (0.0%)	1 (3.8%)	
- 15-independent	12 (85.7%)	11 (91.7%)	23 (88.5%)	
- Missing	5	7	12	
Mobility (on level surfaces)				
- 0-immobile	1 (7.1%)	1 (8.3%)	2 (7.7%)	0.639 *1
- 10-walks with help	1 (7.1%)	0 (0.0%)	1 (3.8%)	
- 15-independent	12 (85.7%)	11 (91.7%)	23 (88.5%)	
- Missing	5	7	12	
Stairs				
- 0-unable	1 (7.1%)	1 (8.3%)	2 (7.7%)	0.910 *1
- 10-independent	13 (92.9%)	11 (91.7%)	24 (92.3%)	
- Missing	5	7	12	

*1 = Chi^2-test, two-sided

10.5.6.2 PP set

Table 10.5.36: Barthel index total (PP set)

	Placebo N=9	Dexamethasone N=7	Total N=16	p-value
Discharge/Day 30 Barthel Index Total				
- N	8	4	12	0.604 *2
- Mean +/- SD	73.1 +/-33.7	58.8 +/-42.1	68.3 +/-35.4	
- Median	87.5	67.5	75.0	
- p25, p75	50.0, 100.0	32.5, 85.0	50.0, 100.0	
- Min, Max	10.0, 100.0	0.0, 100.0	0.0, 100.0	
- Missing	1	3	4	
6 Months Barthel Index Total				
- N	9	5	14	1.000 *2
- Mean +/- SD	88.3 +/-33.2	95.0 +/-11.2	90.7 +/-27.0	
- Median	100.0	100.0	100.0	
- p25, p75	100.0, 100.0	100.0, 100.0	100.0, 100.0	
- Min, Max	0.0, 100.0	75.0, 100.0	0.0, 100.0	
- Missing	0	2	2	
12 Months Barthel Index Total				
- N	7	6	13	1.000 *2
- Mean +/- SD	98.6 +/-3.8	99.2 +/-2.0	98.8 +/-3.0	
- Median	100.0	100.0	100.0	
- p25, p75	100.0, 100.0	100.0, 100.0	100.0, 100.0	
- Min, Max	90.0, 100.0	95.0, 100.0	90.0, 100.0	
- Missing	2	1	3	

*2 = U-test, two-sided

Table 10.5.37: Barthel index items at discharge/day 30 (PP set)

	Placebo N=9	Dexamethasone N=7	Total N=16	p-value
Feeding				
- 0-unable	1 (12.5%)	1 (25.0%)	2 (16.7%)	0.687 *[1]
- 5-needs help	3 (37.5%)	2 (50.0%)	5 (41.7%)	
- 10-independent	4 (50.0%)	1 (25.0%)	5 (41.7%)	
- Missing	1	3	4	
Bathing				
- 0-dependent	3 (37.5%)	3 (75.0%)	6 (50.0%)	0.221 *[1]
- 5-independent	5 (62.5%)	1 (25.0%)	6 (50.0%)	
- Missing	1	3	4	
Grooming				
- 0-needs to help	3 (37.5%)	2 (50.0%)	5 (41.7%)	0.679 *[1]
- 5-independent	5 (62.5%)	2 (50.0%)	7 (58.3%)	
- Missing	1	3	4	
Dressing				
- 0-dependent	1 (12.5%)	1 (25.0%)	2 (16.7%)	0.687 *[1]
- 5-needs help	3 (37.5%)	2 (50.0%)	5 (41.7%)	
- 10-independent	4 (50.0%)	1 (25.0%)	5 (41.7%)	
- Missing	1	3	4	
Bowels				
- 0-incontinent	0 (0.0%)	1 (25.0%)	1 (8.3%)	0.223 *[1]
- 5-occasional accident	2 (25.0%)	0 (0.0%)	2 (16.7%)	
- 10-continent	6 (75.0%)	3 (75.0%)	9 (75.0%)	
- Missing	1	3	4	
Bladder				
- 0-incontinent/catheterized	2 (25.0%)	1 (25.0%)	3 (25.0%)	0.755 *[1]
- 5-occasional accident	1 (12.5%)	0 (0.0%)	1 (8.3%)	
- 10-continent	5 (62.5%)	3 (75.0%)	8 (66.7%)	
- Missing	1	3	4	
Toilet Use				
- 0-dependent	2 (25.0%)	1 (25.0%)	3 (25.0%)	1.000 *[1]
- 5-needs some help	2 (25.0%)	1 (25.0%)	3 (25.0%)	
- 10-independent	4 (50.0%)	2 (50.0%)	6 (50.0%)	
- Missing	1	3	4	
Bladder				
- 0-incontinent/catheterized	2 (25.0%)	1 (25.0%)	3 (25.0%)	0.755 *[1]
- 5-occasional accident	1 (12.5%)	0 (0.0%)	1 (8.3%)	
- 10-continent	5 (62.5%)	3 (75.0%)	8 (66.7%)	
- Missing	1	3	4	

	Placebo N=9	Dexamethasone N=7	Total N=16	p-value
Toilet Use				
- 0-dependent	2 (25.0%)	1 (25.0%)	3 (25.0%)	1.000 *1
- 5-needs some help	2 (25.0%)	1 (25.0%)	3 (25.0%)	
- 10-independent	4 (50.0%)	2 (50.0%)	6 (50.0%)	
- Missing	1	3	4	
Transfer (bed to chair and back)				
- 0-unable	0 (0.0%)	1 (25.0%)	1 (8.3%)	0.345 *1
- 5-major help	1 (12.5%)	1 (25.0%)	2 (16.7%)	
- 10-minor help	2 (25.0%)	0 (0.0%)	2 (16.7%)	
- 15-independent	5 (62.5%)	2 (50.0%)	7 (58.3%)	
- Missing	1	3	4	
Mobility (on level surfaces)				
- 0-immobile	1 (12.5%)	1 (25.0%)	2 (16.7%)	0.519 *1
- 10-walks with help	2 (25.0%)	0 (0.0%)	2 (16.7%)	
- 15-independent	5 (62.5%)	3 (75.0%)	8 (66.7%)	
- Missing	1	3	4	
Stairs				
- 0-unable	1 (12.5%)	2 (50.0%)	3 (25.0%)	0.363 *1
- 5-needs help	3 (37.5%)	1 (25.0%)	4 (33.3%)	
- 10-independent	4 (50.0%)	1 (25.0%)	5 (41.7%)	
- Missing	1	3	4	

*1 = Chi^2-test, two-sided

Table 10.5.38: Barthel index items at month 6 (PP set)

	Placebo N=9	Dexamethasone N=7	Total N=16	p-value
Feeding				
- 0-unable	1 (11.1%)	0 (0.0%)	1 (6.7%)	0.398 *1
- 10-independent	8 (88.9%)	6 (100.0%)	14 (93.3%)	
- Missing	0	1	1	
Bathing				
- 0-dependent	1 (11.1%)	1 (16.7%)	2 (13.3%)	0.756 *1
- 5-independent	8 (88.9%)	5 (83.3%)	13 (86.7%)	
- Missing	0	1	1	
Grooming				
- 0-needs to help	1 (11.1%)	2 (33.3%)	3 (20.0%)	0.292 *1
- 5-independent	8 (88.9%)	4 (66.7%)	12 (80.0%)	
- Missing	0	1	1	
Dressing				
- 0-dependent	1 (11.1%)	0 (0.0%)	1 (6.7%)	0.463 *1
- 5-needs help	1 (11.1%)	0 (0.0%)	1 (6.7%)	
- 10-independent	7 (77.8%)	6 (100.0%)	13 (86.7%)	
- Missing	0	1	1	
Bowels				
- 0-incontinent	1 (11.1%)	0 (0.0%)	1 (6.7%)	0.336 *1
- 5-occasional accident	0 (0.0%)	1 (16.7%)	1 (6.7%)	
- 10-continent	8 (88.9%)	5 (83.3%)	13 (86.7%)	
- Missing	0	1	1	

	Placebo N=9	Dexamethasone N=7	Total N=16	p-value
Bladder				
- 0-incontinent/catheterized	1 (11.1%)	0 (0.0%)	1 (6.7%)	0.336 *[1]
- 5-occasional accident	0 (0.0%)	1 (16.7%)	1 (6.7%)	
- 10-continent	8 (88.9%)	5 (83.3%)	13 (86.7%)	
- Missing	0	1	1	
Toilet Use				
- 0-dependent	1 (11.1%)	0 (0.0%)	1 (6.7%)	0.336 *[1]
- 5-needs some help	0 (0.0%)	1 (16.7%)	1 (6.7%)	
- 10-independent	8 (88.9%)	5 (83.3%)	13 (86.7%)	
- Missing	0	1	1	
Bladder				
- 0-incontinent/catheterized	1 (11.1%)	0 (0.0%)	1 (6.7%)	0.336 *[1]
- 5-occasional accident	0 (0.0%)	1 (16.7%)	1 (6.7%)	
- 10-continent	8 (88.9%)	5 (83.3%)	13 (86.7%)	
- Missing	0	1	1	
Toilet Use				
- 0-dependent	1 (11.1%)	0 (0.0%)	1 (6.7%)	0.336 *[1]
- 5-needs some help	0 (0.0%)	1 (16.7%)	1 (6.7%)	
- 10-independent	8 (88.9%)	5 (83.3%)	13 (86.7%)	
- Missing	0	1	1	
Transfer (bed to chair and back)				
- 0-unable	1 (11.1%)	0 (0.0%)	1 (6.7%)	0.398 *[1]
- 15-independent	8 (88.9%)	6 (100.0%)	14 (93.3%)	
- Missing	0	1	1	
Mobility (on level surfaces)				
- 0-immobile	1 (11.1%)	0 (0.0%)	1 (6.7%)	0.398 *[1]
- 15-independent	8 (88.9%)	6 (100.0%)	14 (93.3%)	
- Missing	0	1	1	
Stairs				
- 0-unable	1 (11.1%)	0 (0.0%)	1 (7.1%)	0.439 *[1]
- 10-independent	8 (88.9%)	5 (100.0%)	13 (92.9%)	
- Missing	0	2	2	

*[1] = Chi^2-test, two-sided

Table 10.5.39: Barthel index items at month 12 (PP set)

	Placebo N=9	Dexamethasone N=7	Total N=16	p-value
Feeding				
- 10-independent	7 (100.0%)	6 (100.0%)	13 (100.0%)	
- Missing	2	1	3	
Bathing				
- 5-independent	7 (100.0%)	6 (100.0%)	13 (100.0%)	
- Missing	2	1	3	
Grooming				
- 0-needs to help	0 (0.0%)	1 (16.7%)	1 (7.7%)	0.261 *[1]
- 5-independent	7 (100.0%)	5 (83.3%)	12 (92.3%)	
- Missing	2	1	3	
Dressing				
- 10-independent	7 (100.0%)	6 (100.0%)	13 (100.0%)	
- Missing	2	1	3	
Bowels				
- 10-continent	7 (100.0%)	6 (100.0%)	13 (100.0%)	
- Missing	2	1	3	
Bladder				
- 10-continent	7 (100.0%)	6 (100.0%)	13 (100.0%)	
- Missing	2	1	3	
Toilet Use				
- 10-independent	7 (100.0%)	6 (100.0%)	13 (100.0%)	
- Missing	2	1	3	
Bladder				
- 10-continent	7 (100.0%)	6 (100.0%)	13 (100.0%)	
- Missing	2	1	3	
Toilet Use				
- 10-independent	7 (100.0%)	6 (100.0%)	13 (100.0%)	
- Missing	2	1	3	
Transfer (bed to chair and back)				
- 10-minor help	1 (14.3%)	0 (0.0%)	1 (7.7%)	0.335 *[1]
- 15-independent	6 (85.7%)	6 (100.0%)	12 (92.3%)	
- Missing	2	1	3	
Mobility (on level surfaces)				
- 10-walks with help	1 (14.3%)	0 (0.0%)	1 (7.7%)	0.335 *[1]
- 15-independent	6 (85.7%)	6 (100.0%)	12 (92.3%)	
- Missing	2	1	3	
Stairs				
- 10-independent	7 (100.0%)	6 (100.0%)	13 (100.0%)	
- Missing	2	1	3	

*[1] = Chi^2-test, two-sided

10.5.7 NIH-scale

10.5.7.1 FAS

Table 10.5.40: NIH total scores (FAS)

	Placebo N=19	Dexamethasone N=19	Total N=38	p-value
Day 0 NIH Total*				
- N	19	19	38	0.482 *2
- Mean +/- SD	7.7 +/-6.6	6.9 +/-7.6	7.3 +/-7.0	
- Median	6.0	5.0	6.0	
- p25, p75	3.0, 10.0	2.0, 9.0	2.0, 9.0	
- Min, Max	0.0, 23.0	0.0, 31.0	0.0, 31.0	
- Missing	0	0	0	
Day 7 NIH Total				
- N	16	19	35	0.461 *2
- Mean +/- SD	6.3 +/-7.4	4.9 +/-6.4	5.6 +/-6.8	
- Median	4.0	1.0	4.0	
- p25, p75	1.0, 9.0	0.0, 6.0	1.0, 8.0	
- Min, Max	0.0, 27.0	0.0, 19.0	0.0, 27.0	
- Missing	3	0	3	
Discharge/Day 30 NIH Total				
- N	18	16	34	0.725 *2
- Mean +/- SD	3.7 +/-4.6	4.1 +/-5.8	3.9 +/-5.2	
- Median	2.0	1.0	1.5	
- p25, p75	0.0, 6.0	0.0, 6.5	0.0, 6.0	
- Min, Max	0.0, 17.0	0.0, 19.0	0.0, 19.0	
- Missing	1	3	4	
6 Months NIH Total				
- N	15	13	28	0.431 *2
- Mean +/- SD	0.7 +/-1.6	0.8 +/-2.0	0.8 +/-1.8	
- Median	0.0	0.0	0.0	
- p25, p75	0.0, 1.0	0.0, 0.0	0.0, 0.5	
- Min, Max	0.0, 6.0	0.0, 7.0	0.0, 7.0	
- Missing	4	6	10	
12 Months NIH Total				
- N	14	12	26	0.429 *2
- Mean +/- SD	1.6 +/-4.2	0.7 +/-1.5	1.2 +/-3.2	
- Median	0.0	0.0	0.0	
- p25, p75	0.0, 1.0	0.0, 0.5	0.0, 1.0	
- Min, Max	0.0, 16.0	0.0, 5.0	0.0, 16.0	
- Missing	5	7	12	

* = no primary endpoint, *2 = U-test, two-sided

Table 10.5.41: NIH scale single items day 7 (FAS)

	Placebo N=19	Dexamethasone N=19	Total N=38	p-value
Level of consciousness				
- 0-Alert	8 (47.1%)	13 (68.4%)	21 (58.3%)	0.406 *1
- 1-Drowsy	5 (29.4%)	2 (10.5%)	7 (19.4%)	
- 2-Stuporous	2 (11.8%)	1 (5.3%)	3 (8.3%)	
- 3-Coma	2 (11.8%)	3 (15.8%)	5 (13.9%)	
- Missing	2	0	2	
Level of consciousness - questions				
- 0-Answers both correctly	8 (50.0%)	10 (52.6%)	18 (51.4%)	0.592 *1
- 1-Answers one correctly	1 (6.3%)	3 (15.8%)	4 (11.4%)	
- 2-Incorrect	7 (43.8%)	6 (31.6%)	13 (37.1%)	
- Missing	3	0	3	
Level of consciousness - commands				
- 0-Obeys both correctly	12 (75.0%)	13 (68.4%)	25 (71.4%)	0.867 *1
- 1-Obeys one correctly	1 (6.3%)	1 (5.3%)	2 (5.7%)	
- 2-Incorrect	3 (18.8%)	5 (26.3%)	8 (22.9%)	
- Missing	3	0	3	
Pupillary response				
- 0-Both reactive	17 (100.0%)	19 (100.0%)	36 (100.0%)	
- Missing	2	0	2	
Best gaze				
- 0-Normal	16 (100.0%)	18 (94.7%)	34 (97.1%)	0.352 *1
- 1-Partial gaze palsy	0 (0.0%)	1 (5.3%)	1 (2.9%)	
- Missing	3	0	3	
Best visual				
- 0-No visual loss	14 (87.5%)	19 (100.0%)	33 (94.3%)	0.284 *1
- 1-Partial hemianopia	1 (6.3%)	0 (0.0%)	1 (2.9%)	
- 2-Complete hemianopia	1 (6.3%)	0 (0.0%)	1 (2.9%)	
- Missing	3	0	3	
Facial palsy				
- 0-Normal	11 (68.8%)	19 (100.0%)	30 (85.7%)	0.031 *1
- 1-Minor	4 (25.0%)	0 (0.0%)	4 (11.4%)	
- 3-Complete	1 (6.3%)	0 (0.0%)	1 (2.9%)	
- Missing	3	0	3	
Best motor - arm				
- 0-No drift	10 (62.5%)	14 (73.7%)	24 (68.6%)	0.554 *1
- 1-Drift	4 (25.0%)	2 (10.5%)	6 (17.1%)	
- 2-Cannot resist gravity	0 (0.0%)	1 (5.3%)	1 (2.9%)	
- 3-No effort against gravity	2 (12.5%)	2 (10.5%)	4 (11.4%)	
- Missing	3	0	3	
Best motor - leg				
- 0-No drift	11 (68.8%)	13 (68.4%)	24 (68.6%)	0.970 *1
- 1-Drift	3 (18.8%)	3 (15.8%)	6 (17.1%)	
- 2-Cannot resist gravity	1 (6.3%)	1 (5.3%)	2 (5.7%)	
- 3-No effort against gravity	1 (6.3%)	2 (10.5%)	3 (8.6%)	
- Missing	3	0	3	

	Placebo N=19	Dexamethasone N=19	Total N=38	p-value
Plantar reflex				
- 0-Normal	13 (81.3%)	17 (89.5%)	30 (85.7%)	0.150 *[1]
- 1-Equivocal	1 (6.3%)	0 (0.0%)	1 (2.9%)	
- 2-One extensor	2 (12.5%)	0 (0.0%)	2 (5.7%)	
- 3-Bilateral extensor	0 (0.0%)	2 (10.5%)	2 (5.7%)	
- Missing	3	0	3	
Limb ataxia				
- 0-Absent	15 (93.8%)	19 (100.0%)	34 (97.1%)	0.269 *[1]
- 1-Present in arm or leg	1 (6.3%)	0 (0.0%)	1 (2.9%)	
- Missing	3	0	3	
Sensory				
- 0-Normal	12 (75.0%)	18 (94.7%)	30 (85.7%)	0.137 *[1]
- 1-Partial loss	3 (18.8%)	0 (0.0%)	3 (8.6%)	
- 2-Dense loss	1 (6.3%)	1 (5.3%)	2 (5.7%)	
- Missing	3	0	3	
Neglect				
- 0-No neglect	12 (75.0%)	18 (94.7%)	30 (85.7%)	0.227 *[1]
- 1-Partial neglect	3 (18.8%)	1 (5.3%)	4 (11.4%)	
- 2-Complete neglect	1 (6.3%)	0 (0.0%)	1 (2.9%)	
- Missing	3	0	3	
Dysarthria				
- 0-Normal articulation	12 (75.0%)	17 (89.5%)	29 (82.9%)	0.446 *[1]
- 1-Mild to moderate dysarthria	3 (18.8%)	1 (5.3%)	4 (11.4%)	
- 2-Near unitelliglible or worse	1 (6.3%)	1 (5.3%)	2 (5.7%)	
- Missing	3	0	3	
Best language				
- 0-No aphasia	6 (37.5%)	7 (36.8%)	13 (37.1%)	0.889 *[1]
- 1-Mild to moderate aphasia	5 (31.3%)	7 (36.8%)	12 (34.3%)	
- 2-Severe aphasia	2 (12.5%)	1 (5.3%)	3 (8.6%)	
- 3-Mute	3 (18.8%)	4 (21.1%)	7 (20.0%)	
- Missing	3	0	3	

*[1] = Chi^2-test, two-sided

Table 10.5.42: NIH scale single items at discharge/day 30 (FAS)

	Placebo N=19	Dexamethasone N=19	Total N=38	p-value
Level of consciousness				
- 0-Alert	15 (83.3%)	14 (82.4%)	29 (82.9%)	0.342 *[1]
- 1-Drowsy	1 (5.6%)	0 (0.0%)	1 (2.9%)	
- 2-Stuporous	2 (11.1%)	1 (5.9%)	3 (8.6%)	
- 3-Coma	0 (0.0%)	2 (11.8%)	2 (5.7%)	
- Missing	1	2	3	
Level of consciousness - questions				
- 0-Answers both correctly	9 (50.0%)	11 (68.8%)	20 (58.8%)	0.252 *[1]
- 1-Answers one correctly	5 (27.8%)	1 (6.3%)	6 (17.6%)	
- 2-Incorrect	4 (22.2%)	4 (25.0%)	8 (23.5%)	
- Missing	1	3	4	
Level of consciousness - commands				
- 0-Obeys both correctly	14 (77.8%)	12 (75.0%)	26 (76.5%)	0.088 *[1]
- 1-Obeys one correctly	3 (16.7%)	0 (0.0%)	3 (8.8%)	
- 2-Incorrect	1 (5.6%)	4 (25.0%)	5 (14.7%)	
- Missing	1	3	4	
Pupillary response				
- 0-Both reactive	18 (100.0%)	17 (100.0%)	35 (100.0%)	
- Missing	1	2	3	
Best gaze				
- 0-Normal	18 (100.0%)	15 (93.8%)	33 (97.1%)	0.282 *[1]
- 1-Partial gaze palsy	0 (0.0%)	1 (6.3%)	1 (2.9%)	
- Missing	1	3	4	
Best visual				
- 0-No visual loss	18 (100.0%)	16 (100.0%)	34 (100.0%)	
- Missing	1	3	4	
Facial palsy				
- 0-Normal	15 (83.3%)	16 (94.1%)	31 (88.6%)	0.316 *[1]
- 1-Minor	3 (16.7%)	1 (5.9%)	4 (11.4%)	
- Missing	1	2	3	
Best motor - arm				
- 0-No drift	15 (83.3%)	10 (62.5%)	25 (73.5%)	0.442 *[1]
- 1-Drift	1 (5.6%)	4 (25.0%)	5 (14.7%)	
- 2-Cannot resist gravity	1 (5.6%)	1 (6.3%)	2 (5.9%)	
- 3-No effort against gravity	1 (5.6%)	1 (6.3%)	2 (5.9%)	
- Missing	1	3	4	
Best motor - leg				
- 0-No drift	14 (77.8%)	12 (75.0%)	26 (76.5%)	0.712 *[1]
- 1-Drift	2 (11.1%)	2 (12.5%)	4 (11.8%)	
- 2-Cannot resist gravity	1 (5.6%)	0 (0.0%)	1 (2.9%)	
- 3-No effort against gravity	1 (5.6%)	2 (12.5%)	3 (8.8%)	
- Missing	1	3	4	
Plantar reflex				
- 0-Normal	15 (83.3%)	17 (100.0%)	32 (91.4%)	0.212 *[1]
- 1-Equivocal	2 (11.1%)	0 (0.0%)	2 (5.7%)	

	Placebo N=19	Dexamethasone N=19	Total N=38	p-value
- 2-One extensor	1 (5.6%)	0 (0.0%)	1 (2.9%)	
- Missing	1	2	3	
Limb ataxia				
- 0-Absent	16 (88.9%)	14 (87.5%)	30 (88.2%)	0.508 *1
- 1-Present in arm or leg	1 (5.6%)	2 (12.5%)	3 (8.8%)	
- 2-Present in arm and leg	1 (5.6%)	0 (0.0%)	1 (2.9%)	
- Missing	1	3	4	
Sensory				
- 0-Normal	17 (94.4%)	15 (93.8%)	32 (94.1%)	0.932 *1
- 2-Dense loss	1 (5.6%)	1 (6.3%)	2 (5.9%)	
- Missing	1	3	4	
Neglect				
- 0-No neglect	17 (94.4%)	16 (100.0%)	33 (97.1%)	0.339 *1
- 1-Partial neglect	1 (5.6%)	0 (0.0%)	1 (2.9%)	
- Missing	1	3	4	
Dysarthria				
- 0-Normal articulation	16 (88.9%)	13 (81.3%)	29 (85.3%)	0.550 *1
- 1-Mild to moderate dysarthria	2 (11.1%)	2 (12.5%)	4 (11.8%)	
- 2-Near unitelliglible or worse	0 (0.0%)	1 (6.3%)	1 (2.9%)	
- Missing	1	3	4	
Best language				
- 0-No aphasia	8 (44.4%)	6 (37.5%)	14 (41.2%)	0.484 *1
- 1-Mild to moderate aphasia	6 (33.3%)	7 (43.8%)	13 (38.2%)	
- 2-Severe aphasia	2 (11.1%)	0 (0.0%)	2 (5.9%)	
- 3-Mute	2 (11.1%)	3 (18.8%)	5 (14.7%)	
- Missing	1	3	4	

*1 = Chi^2-test, two-sided

Table 10.5.43: NIH scale single items at month 6 (FAS)

	Placebo N=19	Dexamethasone N=19	Total N=38	p-value
Level of consciousness				
- 0-Alert	15 (93.8%)	13 (100.0%)	28 (96.6%)	0.359 *1
- 3-Coma	1 (6.3%)	0 (0.0%)	1 (3.4%)	
- Missing	3	6	9	
Level of consciousness - questions				
- 0-Answers both correctly	14 (87.5%)	11 (84.6%)	25 (86.2%)	0.975 *1
- 1-Answers one correctly	1 (6.3%)	1 (7.7%)	2 (6.9%)	
- 2-Incorrect	1 (6.3%)	1 (7.7%)	2 (6.9%)	
- Missing	3	6	9	
Level of consciousness - commands				
- 0-Obeys both correctly	15 (93.8%)	13 (100.0%)	28 (96.6%)	0.359 *1
- 2-Incorrect	1 (6.3%)	0 (0.0%)	1 (3.4%)	
- Missing	3	6	9	
Pupillary response				
- 0-Both reactive	16 (100.0%)	13 (100.0%)	29 (100.0%)	
- Missing	3	6	9	
Best gaze				
- 0-Normal	16 (100.0%)	13 (100.0%)	29 (100.0%)	
- Missing	3	6	9	
Best visual				
- 0-No visual loss	16 (100.0%)	13 (100.0%)	29 (100.0%)	
- Missing	3	6	9	
Facial palsy				
- 0-Normal	13 (81.3%)	13 (100.0%)	26 (89.7%)	0.099 *1
- 1-Minor	3 (18.8%)	0 (0.0%)	3 (10.3%)	
- Missing	3	6	9	
Best motor - arm				
- 0-No drift	14 (87.5%)	12 (92.3%)	26 (89.7%)	0.653 *1
- 1-Drift	1 (6.3%)	1 (7.7%)	2 (6.9%)	
- 2-Cannot resist gravity	1 (6.3%)	0 (0.0%)	1 (3.4%)	
- Missing	3	6	9	
Best motor - leg				
- 0-No drift	14 (87.5%)	12 (92.3%)	26 (89.7%)	0.653 *1
- 1-Drift	1 (6.3%)	1 (7.7%)	2 (6.9%)	
- 2-Cannot resist gravity	1 (6.3%)	0 (0.0%)	1 (3.4%)	
- Missing	3	6	9	
Plantar reflex				
- 0-Normal	15 (93.8%)	13 (100.0%)	28 (96.6%)	0.359 *1
- 1-Equivocal	1 (6.3%)	0 (0.0%)	1 (3.4%)	
- Missing	3	6	9	

	Placebo<br>N=19	Dexamethasone<br>N=19	Total<br>N=38	p-value
Limb ataxia				
- 0-Absent	14 (93.3%)	12 (92.3%)	26 (92.9%)	0.364 *[1]
- 1-Present in arm or leg	1 (6.7%)	0 (0.0%)	1 (3.6%)	
- 2-Present in arm and leg	0 (0.0%)	1 (7.7%)	1 (3.6%)	
- Missing	4	6	10	
Sensory				
- 0-Normal	15 (100.0%)	12 (92.3%)	27 (96.4%)	0.274 *[1]
- 1-Partial loss	0 (0.0%)	1 (7.7%)	1 (3.6%)	
- Missing	4	6	10	
Neglect				
- 0-No neglect	14 (87.5%)	13 (100.0%)	27 (93.1%)	0.418 *[1]
- 1-Partial neglect	1 (6.3%)	0 (0.0%)	1 (3.4%)	
- 2-Complete neglect	1 (6.3%)	0 (0.0%)	1 (3.4%)	
- Missing	3	6	9	
Dysarthria				
- 0-Normal articulation	15 (93.8%)	13 (100.0%)	28 (96.6%)	0.359 *[1]
- 2-Near unitelliglible or worse	1 (6.3%)	0 (0.0%)	1 (3.4%)	
- Missing	3	6	9	
Best language				
- 0-No aphasia	12 (75.0%)	11 (84.6%)	23 (79.3%)	0.624 *[1]
- 1-Mild to moderate aphasia	3 (18.8%)	2 (15.4%)	5 (17.2%)	
- 3-Mute	1 (6.3%)	0 (0.0%)	1 (3.4%)	
- Missing	3	6	9	

*[1] = Chi^2-test, two-sided

Table 10.5.44: NIH scale single items at month 12 (FAS)

	Placebo N=19	Dexamethasone N=19	Total N=38	p-value
Level of consciousness				
- 0-Alert	14 (100.0%)	12 (100.0%)	26 (100.0%)	
- Missing	5	7	12	
Level of consciousness - questions				
- 0-Answers both correctly	11 (78.6%)	11 (91.7%)	22 (84.6%)	0.395 *1
- 1-Answers one correctly	2 (14.3%)	0 (0.0%)	2 (7.7%)	
- 2-Incorrect	1 (7.1%)	1 (8.3%)	2 (7.7%)	
- Missing	5	7	12	
Level of consciousness - commands				
- 0-Obeys both correctly	13 (92.9%)	12 (100.0%)	25 (96.2%)	0.345 *1
- 1-Obeys one correctly	1 (7.1%)	0 (0.0%)	1 (3.8%)	
- Missing	5	7	12	
Pupillary response				
- 0-Both reactive	14 (100.0%)	12 (100.0%)	26 (100.0%)	
- Missing	5	7	12	
Best gaze				
- 0-Normal	13 (92.9%)	12 (100.0%)	25 (96.2%)	0.345 *1
- 2-Forced deviation	1 (7.1%)	0 (0.0%)	1 (3.8%)	
- Missing	5	7	12	
Best visual				
- 0-No visual loss	14 (100.0%)	12 (100.0%)	26 (100.0%)	
- Missing	5	7	12	
Facial palsy				
- 0-Normal	14 (100.0%)	12 (100.0%)	26 (100.0%)	
- Missing	5	7	12	
Best motor - arm				
- 0-No drift	13 (92.9%)	11 (91.7%)	24 (92.3%)	0.363 *1
- 1-Drift	0 (0.0%)	1 (8.3%)	1 (3.8%)	
- 2-Cannot resist gravity	1 (7.1%)	0 (0.0%)	1 (3.8%)	
- Missing	5	7	12	
Best motor - leg				
- 0-No drift	13 (92.9%)	11 (91.7%)	24 (92.3%)	0.363 *1
- 1-Drift	0 (0.0%)	1 (8.3%)	1 (3.8%)	
- 2-Cannot resist gravity	1 (7.1%)	0 (0.0%)	1 (3.8%)	
- Missing	5	7	12	
Plantar reflex				
- 0-Normal	13 (92.9%)	12 (100.0%)	25 (96.2%)	0.345 *1
- 2-One extensor	1 (7.1%)	0 (0.0%)	1 (3.8%)	
- Missing	5	7	12	
Limb ataxia				
- 0-Absent	14 (100.0%)	12 (100.0%)	26 (100.0%)	
- Missing	5	7	12	

	Placebo N=19	Dexamethasone N=19	Total N=38	p-value
Sensory				
- 0-Normal	13 (92.9%)	10 (83.3%)	23 (88.5%)	0.449 *1
- 1-Partial loss	1 (7.1%)	2 (16.7%)	3 (11.5%)	
- Missing	5	7	12	
Neglect				
- 0-No neglect	14 (100.0%)	12 (100.0%)	26 (100.0%)	
- Missing	5	7	12	
Dysarthria				
- 0-Normal articulation	12 (85.7%)	12 (100.0%)	24 (92.3%)	0.395 *1
- 1-Mild to moderate dysarthria	1 (7.1%)	0 (0.0%)	1 (3.8%)	
- 2-Near unitelliglible or worse	1 (7.1%)	0 (0.0%)	1 (3.8%)	
- Missing	5	7	12	
Best language				
- 0-No aphasia	10 (71.4%)	10 (83.3%)	20 (76.9%)	0.591 *1
- 1-Mild to moderate aphasia	3 (21.4%)	2 (16.7%)	5 (19.2%)	
- 3-Mute	1 (7.1%)	0 (0.0%)	1 (3.8%)	
- Missing	5	7	12	

*1 = Chi^2-test, two-sided

10.5.7.2 PP set

Table 10.5.45: NIH total scores (PP set)

	Placebo N=9	Dexamethasone N=7	Total N=16	p-value
Day 0 NIH Total*				
- N	9	7	16	1.000 *2
- Mean +/- SD	6.0 +/-5.8	6.1 +/-5.4	6.1 +/-5.4	
- Median	6.0	5.0	5.5	
- p25, p75	2.0, 8.0	1.0, 8.0	1.5, 8.0	
- Min, Max	0.0, 19.0	1.0, 17.0	0.0, 19.0	
- Missing	0	0	0	
Day 7 NIH Total				
- N	7	7	14	1.000 *2
- Mean +/- SD	6.7 +/-9.8	5.9 +/-8.1	6.3 +/-8.7	
- Median	2.0	1.0	1.5	
- p25, p75	0.0, 10.0	0.0, 16.0	0.0, 10.0	
- Min, Max	0.0, 27.0	0.0, 19.0	0.0, 27.0	
- Missing	2	0	2	
Discharge/Day 30 NIH Total				
- N	8	4	12	0.791 *2
- Mean +/- SD	1.8 +/-2.3	5.3 +/-9.2	2.9 +/-5.4	
- Median	1.0	1.0	1.0	
- p25, p75	0.0, 3.0	0.5, 10.0	0.0, 3.0	
- Min, Max	0.0, 6.0	0.0, 19.0	0.0, 19.0	
- Missing	1	3	4	
6 Months NIH Total				
- N	8	6	14	0.627 *2
- Mean +/- SD	0.5 +/-0.8	0.5 +/-1.2	0.5 +/-0.9	
- Median	0.0	0.0	0.0	
- p25, p75	0.0, 1.0	0.0, 0.0	0.0, 1.0	
- Min, Max	0.0, 2.0	0.0, 3.0	0.0, 3.0	
- Missing	1	1	2	
12 Months NIH Total				
- N	7	6	13	0.333 *2
- Mean +/- SD	0.6 +/-0.8	0.2 +/-0.4	0.4 +/-0.7	
- Median	0.0	0.0	0.0	
- p25, p75	0.0, 1.0	0.0, 0.0	0.0, 1.0	
- Min, Max	0.0, 2.0	0.0, 1.0	0.0, 2.0	
- Missing	2	1	3	

* = no primary endpoint, *2 = U-test, two-sided

Table 10.5.46: NIH scale single items day 7 (PP set)

	Placebo N=9	Dexamethasone N=7	Total N=16	p-value
Level of consciousness				
- 0-Alert	4 (57.1%)	5 (71.4%)	9 (64.3%)	0.485 *1
- 1-Drowsy	1 (14.3%)	0 (0.0%)	1 (7.1%)	
- 2-Stuporous	1 (14.3%)	0 (0.0%)	1 (7.1%)	
- 3-Coma	1 (14.3%)	2 (28.6%)	3 (21.4%)	
- Missing	2	0	2	
Level of consciousness - questions				
- 0-Answers both correctly	4 (57.1%)	3 (42.9%)	7 (50.0%)	0.565 *1
- 1-Answers one correctly	0 (0.0%)	1 (14.3%)	1 (7.1%)	
- 2-Incorrect	3 (42.9%)	3 (42.9%)	6 (42.9%)	
- Missing	2	0	2	
Level of consciousness - commands				
- 0-Obeys both correctly	5 (71.4%)	5 (71.4%)	10 (71.4%)	1.000 *1
- 2-Incorrect	2 (28.6%)	2 (28.6%)	4 (28.6%)	
- Missing	2	0	2	
Pupillary response				
- 0-Both reactive	7 (100.0%)	7 (100.0%)	14 (100.0%)	
- Missing	2	0	2	
Best gaze				
- 0-Normal	7 (100.0%)	6 (85.7%)	13 (92.9%)	0.299 *1
- 1-Partial gaze palsy	0 (0.0%)	1 (14.3%)	1 (7.1%)	
- Missing	2	0	2	
Best visual				
- 0-No visual loss	6 (85.7%)	7 (100.0%)	13 (92.9%)	0.299 *1
- 2-Complete hemianopia	1 (14.3%)	0 (0.0%)	1 (7.1%)	
- Missing	2	0	2	
Facial palsy				
- 0-Normal	5 (71.4%)	7 (100.0%)	12 (85.7%)	0.311 *1
- 1-Minor	1 (14.3%)	0 (0.0%)	1 (7.1%)	
- 3-Complete	1 (14.3%)	0 (0.0%)	1 (7.1%)	
- Missing	2	0	2	
Best motor - arm				
- 0-No drift	4 (57.1%)	5 (71.4%)	9 (64.3%)	0.295 *1
- 1-Drift	2 (28.6%)	0 (0.0%)	2 (14.3%)	
- 3-No effort against gravity	1 (14.3%)	2 (28.6%)	3 (21.4%)	
- Missing	2	0	2	
Best motor - leg				
- 0-No drift	5 (71.4%)	5 (71.4%)	10 (71.4%)	0.513 *1
- 1-Drift	1 (14.3%)	0 (0.0%)	1 (7.1%)	
- 3-No effort against gravity	1 (14.3%)	2 (28.6%)	3 (21.4%)	
- Missing	2	0	2	

	Placebo N=9	Dexamethasone N=7	Total N=16	p-value
Plantar reflex				
- 0-Normal	7 (100.0%)	7 (100.0%)	14 (100.0%)	
- Missing	2	0	2	
Limb ataxia				
- 0-Absent	7 (100.0%)	7 (100.0%)	14 (100.0%)	
- Missing	2	0	2	
Sensory				
- 0-Normal	6 (85.7%)	6 (85.7%)	12 (85.7%)	1.000 *[1]
- 2-Dense loss	1 (14.3%)	1 (14.3%)	2 (14.3%)	
- Missing	2	0	2	
Neglect				
- 0-No neglect	5 (71.4%)	7 (100.0%)	12 (85.7%)	0.311 *[1]
- 1-Partial neglect	1 (14.3%)	0 (0.0%)	1 (7.1%)	
- 2-Complete neglect	1 (14.3%)	0 (0.0%)	1 (7.1%)	
- Missing	2	0	2	
Dysarthria				
- 0-Normal articulation	5 (71.4%)	7 (100.0%)	12 (85.7%)	0.311 *[1]
- 1-Mild to moderate dysarthria	1 (14.3%)	0 (0.0%)	1 (7.1%)	
- 2-Near unitelliglible or worse	1 (14.3%)	0 (0.0%)	1 (7.1%)	
- Missing	2	0	2	
Best language				
- 0-No aphasia	3 (42.9%)	3 (42.9%)	6 (42.9%)	0.721 *[1]
- 1-Mild to moderate aphasia	2 (28.6%)	1 (14.3%)	3 (21.4%)	
- 2-Severe aphasia	0 (0.0%)	1 (14.3%)	1 (7.1%)	
- 3-Mute	2 (28.6%)	2 (28.6%)	4 (28.6%)	
- Missing	2	0	2	

*[1] = Chi^2-test, two-sided

Table 10.5.47: NIH scale single items at discharge/day 30 (PP set)

	Placebo N=9	Dexamethasone N=7	Total N=16	p-value
Level of consciousness				
- 0-Alert	8 (100.0%)	3 (60.0%)	11 (84.6%)	0.052 *1
- 3-Coma	0 (0.0%)	2 (40.0%)	2 (15.4%)	
- Missing	1	2	3	
Level of consciousness - questions				
- 0-Answers both correctly	5 (62.5%)	3 (75.0%)	8 (66.7%)	0.519 *1
- 1-Answers one correctly	2 (25.0%)	0 (0.0%)	2 (16.7%)	
- 2-Incorrect	1 (12.5%)	1 (25.0%)	2 (16.7%)	
- Missing	1	3	4	
Level of consciousness - commands				
- 0-Obeys both correctly	7 (87.5%)	3 (75.0%)	10 (83.3%)	0.279 *1
- 1-Obeys one correctly	1 (12.5%)	0 (0.0%)	1 (8.3%)	
- 2-Incorrect	0 (0.0%)	1 (25.0%)	1 (8.3%)	
- Missing	1	3	4	
Pupillary response				
- 0-Both reactive	8 (100.0%)	5 (100.0%)	13 (100.0%)	
- Missing	1	2	3	
Best gaze				
- 0-Normal	8 (100.0%)	3 (75.0%)	11 (91.7%)	0.140 *1
- 1-Partial gaze palsy	0 (0.0%)	1 (25.0%)	1 (8.3%)	
- Missing	1	3	4	
Best visual				
- 0-No visual loss	8 (100.0%)	4 (100.0%)	12 (100.0%)	
- Missing	1	3	4	
Facial palsy				
- 0-Normal	7 (87.5%)	5 (100.0%)	12 (92.3%)	0.411 *1
- 1-Minor	1 (12.5%)	0 (0.0%)	1 (7.7%)	
- Missing	1	2	3	
Best motor - arm				
- 0-No drift	8 (100.0%)	3 (75.0%)	11 (91.7%)	0.140 *1
- 3-No effort against gravity	0 (0.0%)	1 (25.0%)	1 (8.3%)	
- Missing	1	3	4	
Best motor - leg				
- 0-No drift	7 (87.5%)	3 (75.0%)	10 (83.3%)	0.279 *1
- 1-Drift	1 (12.5%)	0 (0.0%)	1 (8.3%)	
- 3-No effort against gravity	0 (0.0%)	1 (25.0%)	1 (8.3%)	
- Missing	1	3	4	
Plantar reflex				
- 0-Normal	8 (100.0%)	5 (100.0%)	13 (100.0%)	
- Missing	1	2	3	

	Placebo N=9	Dexamethasone N=7	Total N=16	p-value
Limb ataxia				
- 0-Absent	7 (87.5%)	4 (100.0%)	11 (91.7%)	0.460 *[1]
- 1-Present in arm or leg	1 (12.5%)	0 (0.0%)	1 (8.3%)	
- Missing	1	3	4	
Sensory				
- 0-Normal	8 (100.0%)	3 (75.0%)	11 (91.7%)	0.140 *[1]
- 2-Dense loss	0 (0.0%)	1 (25.0%)	1 (8.3%)	
- Missing	1	3	4	
Neglect				
- 0-No neglect	8 (100.0%)	4 (100.0%)	12 (100.0%)	
- Missing	1	3	4	
Dysarthria				
- 0-Normal articulation	7 (87.5%)	4 (100.0%)	11 (91.7%)	0.460 *[1]
- 1-Mild to moderate dysarthria	1 (12.5%)	0 (0.0%)	1 (8.3%)	
- Missing	1	3	4	
Best language				
- 0-No aphasia	4 (50.0%)	1 (25.0%)	5 (41.7%)	0.392 *[1]
- 1-Mild to moderate aphasia	3 (37.5%)	2 (50.0%)	5 (41.7%)	
- 2-Severe aphasia	1 (12.5%)	0 (0.0%)	1 (8.3%)	
- 3-Mute	0 (0.0%)	1 (25.0%)	1 (8.3%)	
- Missing	1	3	4	

Table 10.5.48: NIH scale single items at month 6 (PP set)

	Placebo N=9	Dexamethasone N=7	Total N=16	p-value
Level of consciousness				
- 0-Alert	8 (100.0%)	6 (100.0%)	14 (100.0%)	
- Missing	1	1	2	
Level of consciousness - questions				
- 0-Answers both correctly	7 (87.5%)	5 (83.3%)	12 (85.7%)	0.825 *1
- 1-Answers one correctly	1 (12.5%)	1 (16.7%)	2 (14.3%)	
- Missing	1	1	2	
Level of consciousness - commands				
- 0-Obeys both correctly	8 (100.0%)	6 (100.0%)	14 (100.0%)	
- Missing	1	1	2	
Pupillary response				
- 0-Both reactive	8 (100.0%)	6 (100.0%)	14 (100.0%)	
- Missing	1	1	2	
Best gaze				
- 0-Normal	8 (100.0%)	6 (100.0%)	14 (100.0%)	
- Missing	1	1	2	
Best visual				
- 0-No visual loss	8 (100.0%)	6 (100.0%)	14 (100.0%)	
- Missing	1	1	2	
Facial palsy				
- 0-Normal	7 (87.5%)	6 (100.0%)	13 (92.9%)	0.369 *1
- 1-Minor	1 (12.5%)	0 (0.0%)	1 (7.1%)	
- Missing	1	1	2	
Best motor - arm				
- 0-No drift	8 (100.0%)	6 (100.0%)	14 (100.0%)	
- Missing	1	1	2	
Best motor - leg				
- 0-No drift	8 (100.0%)	6 (100.0%)	14 (100.0%)	
- Missing	1	1	2	
Plantar reflex				
- 0-Normal	8 (100.0%)	6 (100.0%)	14 (100.0%)	
- Missing	1	1	2	
Limb ataxia				
- 0-Absent	8 (100.0%)	6 (100.0%)	14 (100.0%)	
- Missing	1	1	2	
Sensory				
- 0-Normal	8 (100.0%)	5 (83.3%)	13 (92.9%)	0.231 *1
- 1-Partial loss	0 (0.0%)	1 (16.7%)	1 (7.1%)	
- Missing	1	1	2	

	Placebo N=9	Dexamethasone N=7	Total N=16	p-value
Neglect				
- 0-No neglect	8 (100.0%)	6 (100.0%)	14 (100.0%)	
- Missing	1	1	2	
Dysarthria				
- 0-Normal articulation	8 (100.0%)	6 (100.0%)	14 (100.0%)	
- Missing	1	1	2	
Best language				
- 0-No aphasia	6 (75.0%)	5 (83.3%)	11 (78.6%)	0.707 *[1]
- 1-Mild to moderate aphasia	2 (25.0%)	1 (16.7%)	3 (21.4%)	
- Missing	1	1	2	

Table 10.5.49: NIH scale single items at month 12 (PP set)

	Placebo N=9	Dexamethasone N=7	Total N=16	p-value
Level of consciousness				
- 0-Alert	7 (100.0%)	6 (100.0%)	13 (100.0%)	
- Missing	2	1	3	
Level of consciousness - questions				
- 0-Answers both correctly	6 (85.7%)	6 (100.0%)	12 (92.3%)	0.335 *[1]
- 1-Answers one correctly	1 (14.3%)	0 (0.0%)	1 (7.7%)	
- Missing	2	1	3	
Level of consciousness - commands				
- 0-Obeys both correctly	7 (100.0%)	6 (100.0%)	13 (100.0%)	
- Missing	2	1	3	
Pupillary response				
- 0-Both reactive	7 (100.0%)	6 (100.0%)	13 (100.0%)	
- Missing	2	1	3	
Best gaze				
- 0-Normal	7 (100.0%)	6 (100.0%)	13 (100.0%)	
- Missing	2	1	3	
Best visual				
- 0-No visual loss	7 (100.0%)	6 (100.0%)	13 (100.0%)	
- Missing	2	1	3	
Facial palsy				
- 0-Normal	7 (100.0%)	6 (100.0%)	13 (100.0%)	
- Missing	2	1	3	
Best motor - arm				
- 0-No drift	7 (100.0%)	6 (100.0%)	13 (100.0%)	
- Missing	2	1	3	
Best motor - leg				
- 0-No drift	7 (100.0%)	6 (100.0%)	13 (100.0%)	
- Missing	2	1	3	
Plantar reflex				
- 0-Normal	7 (100.0%)	6 (100.0%)	13 (100.0%)	
- Missing	2	1	3	
Limb ataxia				
- 0-Absent	7 (100.0%)	6 (100.0%)	13 (100.0%)	
- Missing	2	1	3	
Sensory				
- 0-Normal	6 (85.7%)	5 (83.3%)	11 (84.6%)	0.906 *[1]
- 1-Partial loss	1 (14.3%)	1 (16.7%)	2 (15.4%)	
- Missing	2	1	3	

	Placebo N=9	Dexamethasone N=7	Total N=16	p-value
Neglect				
- 0-No neglect	7 (100.0%)	6 (100.0%)	13 (100.0%)	
- Missing	2	1	3	
Dysarthria				
- 0-Normal articulation	7 (100.0%)	6 (100.0%)	13 (100.0%)	
- Missing	2	1	3	
Best language				
- 0-No aphasia	5 (71.4%)	6 (100.0%)	11 (84.6%)	0.155 *[1]
- 1-Mild to moderate aphasia	2 (28.6%)	0 (0.0%)	2 (15.4%)	
- Missing	2	1	3	

*[1] = Chi^2-test, two-sided

10.6 Safety

10.6.1 Physical examination and vital signs

10.6.1.1 Vital signs

Table 10.6.1: Vital signs day 7 (FAS)

	Placebo N=19	Dexamethasone N=19	Total N=38	p-value
Weight (kg)				
- N	16	15	31	0.526 *2
- Mean +/- SD	76.2 +/-11.6	84.2 +/-18.4	80.1 +/-15.5	
- Median	77.5	81.3	80.0	
- p25, p75	70.0, 83.0	68.0, 98.0	68.0, 84.0	
- Min, Max	52.5, 100.0	65.0, 124.3	52.5, 124.3	
- Missing	3	4	7	
Temperature (°C)				
- N	17	19	36	0.172 *2
- Mean +/- SD	37.4 +/-0.6	37.1 +/-0.8	37.2 +/-0.7	
- Median	37.5	37.2	37.4	
- p25, p75	36.8, 37.8	36.4, 37.8	36.6, 37.8	
- Min, Max	36.6, 38.2	35.8, 39.0	35.8, 39.0	
- Missing	2	0	2	
Heart rate (bpm)				
- N	18	19	37	0.419 *2
- Mean +/- SD	80.2 +/-15.9	75.2 +/-14.2	77.6 +/-15.1	
- Median	76.0	80.0	76.0	
- p25, p75	68.0, 84.0	60.0, 88.0	66.0, 86.0	
- Min, Max	62.0, 124.0	52.0, 96.0	52.0, 124.0	
- Missing	1	0	1	
Systolic blood pressure (mmHg)				
- N	17	19	36	0.056 *2
- Mean +/- SD	127.2 +/-17.1	134.3 +/-11.0	130.9 +/-14.4	
- Median	121.0	130.0	130.0	
- p25, p75	120.0, 131.0	130.0, 140.0	120.0, 140.0	
- Min, Max	103.0, 175.0	110.0, 150.0	103.0, 175.0	
- Missing	2	0	2	
Diastolic blood pressure (mmHg)				
- N	17	19	36	0.061 *2
- Mean +/- SD	70.5 +/-11.1	77.8 +/-12.1	74.4 +/-12.1	
- Median	70.0	80.0	75.0	
- p25, p75	65.0, 80.0	70.0, 90.0	70.0, 80.0	
- Min, Max	41.0, 90.0	50.0, 100.0	41.0, 100.0	
- Missing	2	0	2	

*2 = U-test, two-sided

Table 10.6.2: Vital signs at discharge/day 30 (FAS)

	Placebo N=19	Dexamethasone N=19	Total N=38	p-value
Weight (kg)				
- N	14	14	28	0.800 *[2]
- Mean +/- SD	75.9 +/-16.0	83.4 +/-19.8	79.7 +/-18.1	
- Median	74.7	76.5	75.0	
- p25, p75	69.0, 86.0	68.0, 98.0	68.5, 86.5	
- Min, Max	39.0, 100.0	65.0, 123.0	39.0, 123.0	
- Missing	5	5	10	
Temperature (°C)				
- N	18	14	32	0.493 *[2]
- Mean +/- SD	36.9 +/-0.4	36.9 +/-0.8	36.9 +/-0.6	
- Median	36.9	36.7	36.8	
- p25, p75	36.6, 37.1	36.4, 37.2	36.5, 37.2	
- Min, Max	36.2, 37.7	36.0, 38.9	36.0, 38.9	
- Missing	1	5	6	
Heart rate (bpm)				
- N	18	15	33	0.842 *[2]
- Mean +/- SD	78.1 +/-16.3	76.3 +/-20.5	77.3 +/-18.0	
- Median	73.0	75.0	74.0	
- p25, p75	68.0, 80.0	60.0, 82.0	68.0, 80.0	
- Min, Max	56.0, 120.0	52.0, 140.0	52.0, 140.0	
- Missing	1	4	5	
Systolic blood pressure (mmHg)				
- N	18	15	33	1.000 *[2]
- Mean +/- SD	129.6 +/-17.1	127.7 +/-12.9	128.7 +/-15.1	
- Median	125.0	130.0	130.0	
- p25, p75	120.0, 140.0	120.0, 140.0	120.0, 140.0	
- Min, Max	110.0, 160.0	110.0, 150.0	110.0, 160.0	
- Missing	1	4	5	
Diastolic blood pressure (mmHg)				
- N	18	15	33	0.365 *[2]
- Mean +/- SD	74.2 +/-8.9	77.5 +/-11.1	75.7 +/-9.9	
- Median	72.5	80.0	78.0	
- p25, p75	70.0, 80.0	70.0, 82.0	70.0, 80.0	
- Min, Max	60.0, 90.0	60.0, 100.0	60.0, 100.0	
- Missing	1	4	5	

*[2] = U-test, two-sided

Table 10.6.3: Vital signs month 6 (FAS)

	Placebo N=19	Dexamethasone N=19	Total N=38	p-value
Weight (kg)				
- N	17	12	29	0.451 *[2]
- Mean +/- SD	73.4 +/-10.3	79.1 +/-19.4	75.8 +/-14.7	
- Median	75.5	78.0	76.0	
- p25, p75	70.0, 76.1	65.2, 88.5	66.4, 85.8	
- Min, Max	54.0, 90.0	55.0, 125.0	54.0, 125.0	
- Missing	2	7	9	
Temperature (°C)				
- N	13	12	25	0.722 *[2]
- Mean +/- SD	36.7 +/-0.4	36.6 +/-0.4	36.6 +/-0.4	
- Median	36.6	36.6	36.6	
- p25, p75	36.3, 37.0	36.4, 36.8	36.3, 36.8	
- Min, Max	36.2, 37.3	36.0, 37.1	36.0, 37.3	
- Missing	6	7	13	
Heart rate (bpm)				
- N	15	13	28	0.061 *[2]
- Mean +/- SD	77.5 +/-15.6	67.9 +/-7.1	73.1 +/-13.2	
- Median	78.0	66.0	72.0	
- p25, p75	66.0, 88.0	62.0, 72.0	63.0, 80.0	
- Min, Max	52.0, 116.0	60.0, 80.0	52.0, 116.0	
- Missing	4	6	10	
Systolic blood pressure (mmHg)				
- N	16	13	29	0.402 *[2]
- Mean +/- SD	133.1 +/-18.1	128.2 +/-14.4	130.9 +/-16.4	
- Median	130.0	125.0	130.0	
- p25, p75	122.5, 149.0	120.0, 135.0	120.0, 140.0	
- Min, Max	100.0, 165.0	100.0, 160.0	100.0, 165.0	
- Missing	3	6	9	
Diastolic blood pressure (mmHg)				
- N	16	13	29	0.546 *[2]
- Mean +/- SD	79.5 +/-12.3	77.3 +/-7.3	78.5 +/-10.2	
- Median	80.0	80.0	80.0	
- p25, p75	70.0, 86.0	70.0, 80.0	70.0, 85.0	
- Min, Max	60.0, 100.0	70.0, 90.0	60.0, 100.0	
- Missing	3	6	9	

*[2] = U-test, two-sided

Table 10.6.4: Vital signs month 12 (FAS)

	Placebo N=19	Dexamethasone N=19	Total N=38	p-value
Weight (kg)				
- N	13	12	25	0.446 *2
- Mean +/- SD	75.4 +/-8.3	82.0 +/-17.1	78.6 +/-13.4	
- Median	77.0	79.5	78.0	
- p25, p75	72.3, 80.0	69.8, 89.5	70.0, 84.0	
- Min, Max	59.0, 88.0	65.0, 127.5	59.0, 127.5	
- Missing	6	7	13	
Temperature (°C)				
- N	12	10	22	0.765 *2
- Mean +/- SD	36.6 +/-0.4	36.6 +/-0.4	36.6 +/-0.4	
- Median	36.6	36.4	36.6	
- p25, p75	36.3, 36.9	36.3, 37.0	36.3, 37.0	
- Min, Max	36.0, 37.1	36.0, 37.0	36.0, 37.1	
- Missing	7	9	16	
Heart rate (bpm)				
- N	14	12	26	0.336 *2
- Mean +/- SD	71.0 +/-7.7	74.3 +/-8.2	72.5 +/-7.9	
- Median	72.0	73.5	72.0	
- p25, p75	65.0, 78.0	70.0, 79.0	67.0, 78.0	
- Min, Max	56.0, 84.0	60.0, 88.0	56.0, 88.0	
- Missing	5	7	12	
Systolic blood pressure (mmHg)				
- N	14	12	26	0.564 *2
- Mean +/- SD	130.0 +/-13.5	129.4 +/-21.4	129.7 +/-17.2	
- Median	130.0	130.0	130.0	
- p25, p75	125.0, 140.0	119.0, 135.0	120.0, 140.0	
- Min, Max	100.0, 153.0	90.0, 175.0	90.0, 175.0	
- Missing	5	7	12	
Diastolic blood pressure (mmHg)				
- N	14	12	26	0.345 *2
- Mean +/- SD	83.1 +/-8.0	78.8 +/-11.2	81.1 +/-9.7	
- Median	82.5	80.0	80.0	
- p25, p75	80.0, 85.0	72.5, 90.0	77.0, 90.0	
- Min, Max	70.0, 100.0	60.0, 93.0	60.0, 100.0	
- Missing	5	7	12	

*2 = U-test, two-sided

10.6.1.2 Physical examination

Table 10.6.5: Physical examination day 7 (FAS)

	Placebo N=19	Dexamethasone N=19	Total N=38	p-value
General appearance				
- normal	15 (83.3%)	13 (68.4%)	28 (75.7%)	0.291 *1
- abnormal	3 (16.7%)	6 (31.6%)	9 (24.3%)	
- Missing	1	0	1	
Head & Neck				
- normal	18 (100.0%)	17 (89.5%)	35 (94.6%)	0.157 *1
- abnormal	0 (0.0%)	2 (10.5%)	2 (5.4%)	
- Missing	1	0	1	
Eyes & Ears				
- normal	17 (94.4%)	15 (78.9%)	32 (86.5%)	0.168 *1
- abnormal	1 (5.6%)	4 (21.1%)	5 (13.5%)	
- Missing	1	0	1	
Nose & Throat				
- normal	17 (94.4%)	18 (94.7%)	35 (94.6%)	0.969 *1
- abnormal	1 (5.6%)	1 (5.3%)	2 (5.4%)	
- Missing	1	0	1	
Chest				
- normal	15 (83.3%)	19 (100.0%)	34 (91.9%)	0.063 *1
- abnormal	3 (16.7%)	0 (0.0%)	3 (8.1%)	
- Missing	1	0	1	
Lungs				
- normal	14 (77.8%)	18 (94.7%)	32 (86.5%)	0.132 *1
- abnormal	4 (22.2%)	1 (5.3%)	5 (13.5%)	
- Missing	1	0	1	
Heart				
- normal	16 (88.9%)	19 (100.0%)	35 (94.6%)	0.135 *1
- abnormal	2 (11.1%)	0 (0.0%)	2 (5.4%)	
- Missing	1	0	1	
Abdomen				
- normal	17 (94.4%)	17 (89.5%)	34 (91.9%)	0.580 *1
- abnormal	1 (5.6%)	2 (10.5%)	3 (8.1%)	
- Missing	1	0	1	
Extremities & Joints				
- normal	14 (77.8%)	18 (94.7%)	32 (86.5%)	0.132 *1
- abnormal	4 (22.2%)	1 (5.3%)	5 (13.5%)	
- Missing	1	0	1	
Lymph Nodes				
- normal	18 (100.0%)	19 (100.0%)	37 (100.0%)	
- Missing	1	0	1	
Skin				
- normal	13 (72.2%)	17 (89.5%)	30 (81.1%)	0.181 *1
- abnormal	5 (27.8%)	2 (10.5%)	7 (18.9%)	
- Missing	1	0	1	

*2 = U-test, two-sided

Table 10.6.6: Physical examination discharge/day 30 (FAS)

	Placebo N=19	Dexamethasone N=19	Total N=38	p-value
General appearance				
- normal	16 (88.9%)	16 (94.1%)	32 (91.4%)	0.581 *1
- abnormal	2 (11.1%)	1 (5.9%)	3 (8.6%)	
- Missing	1	2	3	
Head & Neck				
- normal	18 (100.0%)	16 (94.1%)	34 (97.1%)	0.296 *1
- abnormal	0 (0.0%)	1 (5.9%)	1 (2.9%)	
- Missing	1	2	3	
Eyes & Ears				
- normal	18 (100.0%)	16 (94.1%)	34 (97.1%)	0.296 *1
- abnormal	0 (0.0%)	1 (5.9%)	1 (2.9%)	
- Missing	1	2	3	
Nose & Throat				
- normal	16 (88.9%)	16 (94.1%)	32 (91.4%)	0.581 *1
- abnormal	2 (11.1%)	1 (5.9%)	3 (8.6%)	
- Missing	1	2	3	
Chest				
- normal	17 (94.4%)	17 (100.0%)	34 (97.1%)	0.324 *1
- abnormal	1 (5.6%)	0 (0.0%)	1 (2.9%)	
- Missing	1	2	3	
Lungs				
- normal	16 (88.9%)	16 (94.1%)	32 (91.4%)	0.581 *1
- abnormal	2 (11.1%)	1 (5.9%)	3 (8.6%)	
- Missing	1	2	3	
Heart				
- normal	15 (83.3%)	17 (100.0%)	32 (91.4%)	0.078 *1
- abnormal	3 (16.7%)	0 (0.0%)	3 (8.6%)	
- Missing	1	2	3	
Abdomen				
- normal	18 (100.0%)	17 (100.0%)	35 (100.0%)	
- Missing	1	2	3	
Extremities & Joints				
- normal	17 (94.4%)	16 (94.1%)	33 (94.3%)	0.967 *1
- abnormal	1 (5.6%)	1 (5.9%)	2 (5.7%)	
- Missing	1	2	3	
Lymph Nodes				
- normal	18 (100.0%)	17 (100.0%)	35 (100.0%)	
- Missing	1	2	3	
Skin				
- normal	17 (94.4%)	15 (88.2%)	32 (91.4%)	0.512 *1
- abnormal	1 (5.6%)	2 (11.8%)	3 (8.6%)	
- Missing	1	2	3	

*1 = Chi^2-test, two-sided

Table 10.6.7: Physical examination month 6 (FAS)

	Placebo N=19	Dexamethasone N=19	Total N=38	p-value
General appearance				
- normal	14 (82.4%)	13 (100.0%)	27 (90.0%)	0.110 *1
- abnormal	3 (17.6%)	0 (0.0%)	3 (10.0%)	
- Missing	2	6	8	
Head & Neck				
- normal	15 (88.2%)	12 (92.3%)	27 (90.0%)	0.665 *1
- abnormal	1 (5.9%)	1 (7.7%)	2 (6.7%)	
- not done	1 (5.9%)	0 (0.0%)	1 (3.3%)	
- Missing	2	6	8	
Eyes & Ears				
- normal	14 (82.4%)	12 (92.3%)	26 (86.7%)	0.427 *1
- abnormal	3 (17.6%)	1 (7.7%)	4 (13.3%)	
- Missing	2	6	8	
Nose & Throat				
- normal	14 (82.4%)	13 (100.0%)	27 (90.0%)	0.110 *1
- abnormal	3 (17.6%)	0 (0.0%)	3 (10.0%)	
- Missing	2	6	8	
Chest				
- normal	17 (100.0%)	13 (100.0%)	30 (100.0%)	
- Missing	2	6	8	
Lungs				
- normal	16 (94.1%)	13 (100.0%)	29 (96.7%)	0.374 *1
- abnormal	1 (5.9%)	0 (0.0%)	1 (3.3%)	
- Missing	2	6	8	
Heart				
- normal	14 (82.4%)	13 (100.0%)	27 (90.0%)	0.110 *1
- abnormal	3 (17.6%)	0 (0.0%)	3 (10.0%)	
- Missing	2	6	8	
Abdomen				
- normal	14 (82.4%)	13 (100.0%)	27 (90.0%)	0.280 *1
- abnormal	2 (11.8%)	0 (0.0%)	2 (6.7%)	
- not done	1 (5.9%)	0 (0.0%)	1 (3.3%)	
- Missing	2	6	8	
Extremities & Joints				
- normal	14 (82.4%)	12 (92.3%)	26 (86.7%)	0.427 *1
- abnormal	3 (17.6%)	1 (7.7%)	4 (13.3%)	
- Missing	2	6	8	
Lymph Nodes				
- normal	16 (94.1%)	13 (100.0%)	29 (96.7%)	0.374 *1
- not done	1 (5.9%)	0 (0.0%)	1 (3.3%)	
- Missing	2	6	8	
Skin				
- normal	14 (82.4%)	12 (92.3%)	26 (86.7%)	0.427 *1
- abnormal	3 (17.6%)	1 (7.7%)	4 (13.3%)	
- Missing	2	6	8	

*1 = Chi^2-test, two-sided

Table 10.6.8: Physical examination month 12 (FAS)

	Placebo N=19	Dexamethasone N=19	Total N=38	p-value
General appearance				
- normal	14 (100.0%)	12 (100.0%)	26 (100.0%)	
- Missing	5	7	12	
Head & Neck				
- normal	13 (92.9%)	12 (100.0%)	25 (96.2%)	0.345 *[1]
- abnormal	1 (7.1%)	0 (0.0%)	1 (3.8%)	
- Missing	5	7	12	
Eyes & Ears				
- normal	13 (92.9%)	10 (83.3%)	23 (88.5%)	0.449 *[1]
- abnormal	1 (7.1%)	2 (16.7%)	3 (11.5%)	
- Missing	5	7	12	
Nose & Throat				
- normal	14 (100.0%)	12 (100.0%)	26 (100.0%)	
- Missing	5	7	12	
Chest				
- normal	14 (100.0%)	12 (100.0%)	26 (100.0%)	
- Missing	5	7	12	
Lungs				
- normal	14 (100.0%)	12 (100.0%)	26 (100.0%)	
- Missing	5	7	12	
Heart				
- normal	12 (85.7%)	12 (100.0%)	24 (92.3%)	0.173 *[1]
- abnormal	2 (14.3%)	0 (0.0%)	2 (7.7%)	
- Missing	5	7	12	
Abdomen				
- normal	13 (92.9%)	12 (100.0%)	25 (96.2%)	0.345 *[1]
- abnormal	1 (7.1%)	0 (0.0%)	1 (3.8%)	
- Missing	5	7	12	
Extremities & Joints				
- normal	12 (85.7%)	12 (100.0%)	24 (92.3%)	0.173 *[1]
- abnormal	2 (14.3%)	0 (0.0%)	2 (7.7%)	
- Missing	5	7	12	
Lymph Nodes				
- normal	14 (100.0%)	11 (91.7%)	25 (96.2%)	0.271 *[1]
- abnormal	0 (0.0%)	1 (8.3%)	1 (3.8%)	
- Missing	5	7	12	
Skin				
- normal	13 (92.9%)	10 (83.3%)	23 (88.5%)	0.449 *[1]
- abnormal	1 (7.1%)	2 (16.7%)	3 (11.5%)	
- Missing	5	7	12	

*[1] = Chi^2-test, two-sided

10.6.2 Adverse events

Table 10.6.9: Patients with (serious) adverse events

	Placebo		Dexamethasone		Total	
	# AE	# patients with AE	# AE	# patients with AE	# AE	# patients with AE
Number of adverse events	72	14 (73.7%)	52	17 (89.5%)	124	31 (81.6%)
Number of serious adverse events	10	6 (31.6%)	9	5 (26.3%)	19	11 (28.9%)

Table 10.6.10: Characteristics of serious adverse events

	Placebo		Dexamethasone		Total	
	# AE	# patients with AE	# AE	# patients with AE	# AE	# patients with AE
MedDRA SOC term						
- General disorders and administration site conditions	1	1 (5.3%)	1	1 (5.3%)	2	2 (5.3%)
- Hepatobiliary disorders	1	1 (5.3%)	0	0 (0.0%)	1	1 (2.6%)
- Infections and infestations	6	5 (26.3%)	1	1 (5.3%)	7	6 (15.8%)
- Investigations	0	0 (0.0%)	1	1 (5.3%)	1	1 (2.6%)
- Renal and urinary disorders	1	1 (5.3%)	1	1 (5.3%)	2	2 (5.3%)
- Respiratory, thoracic and mediastinal disorders	0	0 (0.0%)	2	2 (10.5%)	2	2 (5.3%)
- Skin and subcutaneous tissue disorders	0	0 (0.0%)	1	1 (5.3%)	1	1 (2.6%)
- Surgical and medical procedures	0	0 (0.0%)	2	1 (5.3%)	2	1 (2.6%)
- Vascular disorders	1	1 (5.3%)	0	0 (0.0%)	1	1 (2.6%)
Severity						
- mild	1	1 (5.3%)	1	1 (5.3%)	2	2 (5.3%)
- moderate	2	2 (10.5%)	5	3 (15.8%)	7	5 (13.2%)
- severe	7	3 (15.8%)	3	3 (15.8%)	10	6 (15.8%)
Causality to study drug						
- none	10	6 (31.6%)	5	4 (21.1%)	15	10 (26.3%)
- unlikely	0	0 (0.0%)	3	1 (5.3%)	3	1 (2.6%)
- not assessable	0	0 (0.0%)	1	1 (5.3%)	1	1 (2.6%)
Outcome						
- recovered completely	5	4 (21.1%)	2	1 (5.3%)	7	5 (13.2%)
- recovered with sequelae	0	0 (0.0%)	4	1 (5.3%)	4	1 (2.6%)
- death	5	2 (10.5%)	1	1 (5.3%)	6	3 (7.9%)
- unknown	0	0 (0.0%)	2	2 (10.5%)	2	2 (5.3%)

Table 10.6.11: Characteristics of adverse events

	Placebo		Dexamethasone		Total	
	# AE	# patients with AE	# AE	# patients with AE	# AE	# patients with AE
MedDRA SOC term						
- Blood and lymphatic system disorders	0	0 (0.0%)	2	2 (10.5%)	2	2 (5.3%)
- Cardiac disorders	1	1 (5.3%)	1	1 (5.3%)	2	2 (5.3%)
- Congenital, familial and genetic disorders	1	1 (5.3%)	0	0 (0.0%)	1	1 (2.6%)
- Ear and labyrinth disorders	0	0 (0.0%)	1	1 (5.3%)	1	1 (2.6%)
- Endocrine disorders	0	0 (0.0%)	1	1 (5.3%)	1	1 (2.6%)
- Eye disorders	1	1 (5.3%)	3	3 (15.8%)	4	4 (10.5%)
- Gastrointestinal disorders	5	3 (15.8%)	0	0 (0.0%)	5	3 (7.9%)
- General disorders and administration site conditions	13	7 (36.8%)	8	7 (36.8%)	21	14 (36.8%)
- Hepatobiliary disorders	1	1 (5.3%)	0	0 (0.0%)	1	1 (2.6%)
- Infections and infestations	13	9 (47.4%)	5	5 (26.3%)	18	14 (36.8%)
- Injury, poisoning and procedural complications	2	2 (10.5%)	2	1 (5.3%)	4	3 (7.9%)
- Investigations	8	5 (26.3%)	3	2 (10.5%)	11	7 (18.4%)
- Metabolism and nutrition disorders	3	3 (15.8%)	1	1 (5.3%)	4	4 (10.5%)
- Musculoskeletal and connective tissue disorders	3	3 (15.8%)	3	1 (5.3%)	6	4 (10.5%)
- Nervous system disorders	5	5 (26.3%)	8	6 (31.6%)	13	11 (28.9%)
- Psychiatric disorders	2	2 (10.5%)	2	2 (10.5%)	4	4 (10.5%)
- Renal and urinary disorders	1	1 (5.3%)	2	2 (10.5%)	3	3 (7.9%)
- Respiratory, thoracic and mediastinal disorders	2	2 (10.5%)	3	3 (15.8%)	5	5 (13.2%)
- Skin and subcutaneous tissue disorders	4	4 (21.1%)	2	2 (10.5%)	6	6 (15.8%)
- Social circumstances	0	0 (0.0%)	1	1 (5.3%)	1	1 (2.6%)
- Surgical and medical procedures	3	1 (5.3%)	2	1 (5.3%)	5	2 (5.3%)
- Vascular disorders	4	4 (21.1%)	2	2 (10.5%)	6	6 (15.8%)
Severity						
- mild	38	10 (52.6%)	28	15 (78.9%)	66	25 (65.8%)
- moderate	25	8 (42.1%)	18	9 (47.4%)	43	17 (44.7%)
- severe	9	5 (26.3%)	6	5 (26.3%)	15	10 (26.3%)
Causality to study drug						
- none	58	12 (63.2%)	38	14 (73.7%)	96	26 (68.4%)
- unlikely	10	4 (21.1%)	9	5 (26.3%)	19	9 (23.7%)
- possible	2	2 (10.5%)	3	3 (15.8%)	5	5 (13.2%)
- probable	0	0 (0.0%)	1	1 (5.3%)	1	1 (2.6%)
- not assessable	2	1 (5.3%)	1	1 (5.3%)	3	2 (5.3%)
Outcome						
- ongoing	12	5 (26.3%)	14	8 (42.1%)	26	13 (34.2%)
- recovered completely	50	12 (63.2%)	29	13 (68.4%)	79	25 (65.8%)
- recovered with sequelae	1	1 (5.3%)	4	1 (5.3%)	5	2 (5.3%)
- death	5	2 (10.5%)	1	1 (5.3%)	6	3 (7.9%)
- unknown	4	2 (10.5%)	4	4 (21.1%)	8	6 (15.8%)

10.6.2.1 Adverse events according to severity

Table 10.6.12: System Organ Class (SOC) according to severity - group: Placebo

	mild		moderate		severe		Total	
	#AE	# patients with AE	#AE	# patients with AE	#AE	# patients with AE	#AE	# patients with AE
System organ class								
- Cardiac disorders	0	0 (0.0%)	1	1 (5.3%)	0	0 (0.0%)	1	1 (5.3%)
- Congenital, familial and genetic disorders	1	1 (5.3%)	0	0 (0.0%)	0	0 (0.0%)	1	1 (5.3%)
- Eye disorders	1	1 (5.3%)	0	0 (0.0%)	0	0 (0.0%)	1	1 (5.3%)
- Gastrointestinal disorders	5	3 (15.8%)	0	0 (0.0%)	0	0 (0.0%)	5	3 (15.8%)
- General disorders and administration site conditions	6	2 (10.5%)	5	5 (26.3%)	2	2 (10.5%)	13	7 (36.8%)
- Hepatobiliary disorders	0	0 (0.0%)	0	0 (0.0%)	1	1 (5.3%)	1	1 (5.3%)
- Infections and infestations	4	3 (15.8%)	6	5 (26.3%)	3	2 (10.5%)	13	9 (47.4%)
- Injury, poisoning and procedural complications	2	2 (10.5%)	0	0 (0.0%)	0	0 (0.0%)	2	2 (10.5%)
- Investigations	7	5 (26.3%)	1	1 (5.3%)	0	0 (0.0%)	8	5 (26.3%)
- Metabolism and nutrition disorders	2	2 (10.5%)	1	1 (5.3%)	0	0 (0.0%)	3	3 (15.8%)
- Musculoskeletal and connective tissue disorders	1	1 (5.3%)	2	2 (10.5%)	0	0 (0.0%)	3	3 (15.8%)
- Nervous system disorders	3	3 (15.8%)	2	2 (10.5%)	0	0 (0.0%)	5	5 (26.3%)
- Psychiatric disorders	1	1 (5.3%)	1	1 (5.3%)	0	0 (0.0%)	2	2 (10.5%)
- Renal and urinary disorders	0	0 (0.0%)	0	0 (0.0%)	1	1 (5.3%)	1	1 (5.3%)
- Respiratory, thoracic and mediastinal disorders	1	1 (5.3%)	1	1 (5.3%)	0	0 (0.0%)	2	2 (10.5%)
- Skin and subcutaneous tissue disorders	3	3 (15.8%)	1	1 (5.3%)	0	0 (0.0%)	4	4 (21.1%)
- Surgical and medical procedures	0	0 (0.0%)	2	1 (5.3%)	1	1 (5.3%)	3	1 (5.3%)
- Vascular disorders	1	1 (5.3%)	2	2 (10.5%)	1	1 (5.3%)	4	4 (21.1%)

Table 10.6.13: System Organ Class (SOC) according to severity - group: Dexamethasone

	mild		moderate		severe		Total	
	#AE	# patients with AE	#AE	# patients with AE	#AE	# patients with AE	#AE	# patients with AE
System organ class								
- Blood and lymphatic system disorders	1	1 (5.3%)	1	1 (5.3%)	0	0 (0.0%)	2	2 (10.5%)
- Cardiac disorders	1	1 (5.3%)	0	0 (0.0%)	0	0 (0.0%)	1	1 (5.3%)
- Ear and labyrinth disorders	1	1 (5.3%)	0	0 (0.0%)	0	0 (0.0%)	1	1 (5.3%)
- Endocrine disorders	0	0 (0.0%)	1	1 (5.3%)	0	0 (0.0%)	1	1 (5.3%)
- Eye disorders	3	3 (15.8%)	0	0 (0.0%)	0	0 (0.0%)	3	3 (15.8%)
- General disorders and administration site conditions	6	5 (26.3%)	2	2 (10.5%)	0	0 (0.0%)	8	7 (36.8%)
- Infections and infestations	0	0 (0.0%)	3	3 (15.8%)	2	2 (10.5%)	5	5 (26.3%)
- Injury, poisoning and procedural complications	0	0 (0.0%)	2	1 (5.3%)	0	0 (0.0%)	2	1 (5.3%)
- Investigations	3	2 (10.5%)	0	0 (0.0%)	0	0 (0.0%)	3	2 (10.5%)
- Metabolism and nutrition disorders	1	1 (5.3%)	0	0 (0.0%)	0	0 (0.0%)	1	1 (5.3%)
- Musculoskeletal and connective tissue disorders	1	1 (5.3%)	2	1 (5.3%)	0	0 (0.0%)	3	1 (5.3%)
- Nervous system disorders	4	4 (21.1%)	3	3 (15.8%)	1	1 (5.3%)	8	6 (31.6%)
- Psychiatric disorders	2	2 (10.5%)	0	0 (0.0%)	0	0 (0.0%)	2	2 (10.5%)
- Renal and urinary disorders	1	1 (5.3%)	0	0 (0.0%)	1	1 (5.3%)	2	2 (10.5%)
- Respiratory, thoracic and mediastinal disorders	0	0 (0.0%)	1	1 (5.3%)	2	2 (10.5%)	3	3 (15.8%)
- Skin and subcutaneous tissue disorders	1	1 (5.3%)	1	1 (5.3%)	0	0 (0.0%)	2	2 (10.5%)
- Social circumstances	1	1 (5.3%)	0	0 (0.0%)	0	0 (0.0%)	1	1 (5.3%)
- Surgical and medical procedures	0	0 (0.0%)	2	1 (5.3%)	0	0 (0.0%)	2	1 (5.3%)
- Vascular disorders	2	2 (10.5%)	0	0 (0.0%)	0	0 (0.0%)	2	2 (10.5%)

Table 10.6.14: SOC/PT according to severity - group: Placebo

	mild		moderate		severe		Total	
	#AE	# patients with AE	#AE	# patients with AE	#AE	# patients with AE	#AE	# patients with AE
SOC: Cardiac disorders								
- Atrial fibrillation	0	0 (0.0%)	1	1 (5.3%)	0	0 (0.0%)	1	1 (5.3%)
SOC: Congenital_ familial and genetic disorders								
- Hydrocele	1	1 (5.3%)	0	0 (0.0%)	0	0 (0.0%)	1	1 (5.3%)
SOC: Eye disorders								
- Visual impairment	1	1 (5.3%)	0	0 (0.0%)	0	0 (0.0%)	1	1 (5.3%)
SOC: Gastrointestinal disorders								
- Constipation	1	1 (5.3%)	0	0 (0.0%)	0	0 (0.0%)	1	1 (5.3%)
- Diarrhoea	1	1 (5.3%)	0	0 (0.0%)	0	0 (0.0%)	1	1 (5.3%)
- Nausea	1	1 (5.3%)	0	0 (0.0%)	0	0 (0.0%)	1	1 (5.3%)
- Vomiting	2	2 (10.5%)	0	0 (0.0%)	0	0 (0.0%)	2	2 (10.5%)
SOC: General disorders and administration site conditions								
- Condition aggravated	0	0 (0.0%)	0	0 (0.0%)	1	1 (5.3%)	1	1 (5.3%)
- General physical health deterioration	0	0 (0.0%)	0	0 (0.0%)	1	1 (5.3%)	1	1 (5.3%)
- Infusion site extravasation	0	0 (0.0%)	1	1 (5.3%)	0	0 (0.0%)	1	1 (5.3%)
- Local swelling	1	1 (5.3%)	0	0 (0.0%)	0	0 (0.0%)	1	1 (5.3%)
- Oedema peripheral	1	1 (5.3%)	0	0 (0.0%)	0	0 (0.0%)	1	1 (5.3%)
- Pain	1	1 (5.3%)	0	0 (0.0%)	0	0 (0.0%)	1	1 (5.3%)
- Pyrexia	3	1 (5.3%)	4	4 (21.1%)	0	0 (0.0%)	7	4 (21.1%)
SOC: Hepatobiliary disorders								
- Cholangitis	0	0 (0.0%)	0	0 (0.0%)	1	1 (5.3%)	1	1 (5.3%)
SOC: Infections and infestations								
- Abscess	0	0 (0.0%)	0	0 (0.0%)	1	1 (5.3%)	1	1 (5.3%)
- Bacteraemia	1	1 (5.3%)	0	0 (0.0%)	0	0 (0.0%)	1	1 (5.3%)
- Bacterial infection	1	1 (5.3%)	0	0 (0.0%)	0	0 (0.0%)	1	1 (5.3%)
- Brain abscess	0	0 (0.0%)	1	1 (5.3%)	0	0 (0.0%)	1	1 (5.3%)
- Bronchitis	0	0 (0.0%)	1	1 (5.3%)	0	0 (0.0%)	1	1 (5.3%)
- Bronchopneumonia	0	0 (0.0%)	2	1 (5.3%)	0	0 (0.0%)	2	1 (5.3%)
- Herpes simplex	1	1 (5.3%)	1	1 (5.3%)	0	0 (0.0%)	2	2 (10.5%)
- Pneumonia	0	0 (0.0%)	1	1 (5.3%)	0	0 (0.0%)	1	1 (5.3%)
- Sepsis	0	0 (0.0%)	0	0 (0.0%)	2	2 (10.5%)	2	2 (10.5%)
- Urinary tract infection	1	1 (5.3%)	0	0 (0.0%)	0	0 (0.0%)	1	1 (5.3%)

	mild		moderate		severe		Total	
	#AE	# patients with AE	#AE	# patients with AE	#AE	# patients with AE	#AE	# patients with AE
SOC: Injury_ poisoning and procedural complications								
- Fall	2	2 (10.5%)	0	0 (0.0%)	0	0 (0.0%)	2	2 (10.5%)
SOC: Investigations								
- Alanine aminotransferase increased	0	0 (0.0%)	1	1 (5.3%)	0	0 (0.0%)	1	1 (5.3%)
- Blood acid phosphatase increased	1	1 (5.3%)	0	0 (0.0%)	0	0 (0.0%)	1	1 (5.3%)
- Blood creatinine increased	1	1 (5.3%)	0	0 (0.0%)	0	0 (0.0%)	1	1 (5.3%)
- Blood culture positive	1	1 (5.3%)	0	0 (0.0%)	0	0 (0.0%)	1	1 (5.3%)
- Body temperature increased	1	1 (5.3%)	0	0 (0.0%)	0	0 (0.0%)	1	1 (5.3%)
- C-reactive protein increased	1	1 (5.3%)	0	0 (0.0%)	0	0 (0.0%)	1	1 (5.3%)
- Lipase increased	1	1 (5.3%)	0	0 (0.0%)	0	0 (0.0%)	1	1 (5.3%)
- Weight decreased	1	1 (5.3%)	0	0 (0.0%)	0	0 (0.0%)	1	1 (5.3%)
SOC: Metabolism and nutrition disorders								
- Decreased appetite	1	1 (5.3%)	0	0 (0.0%)	0	0 (0.0%)	1	1 (5.3%)
- Hypokalaemia	1	1 (5.3%)	1	1 (5.3%)	0	0 (0.0%)	2	2 (10.5%)
SOC: Musculoskeletal and connective tissue disorders								
- Muscle contracture	0	0 (0.0%)	1	1 (5.3%)	0	0 (0.0%)	1	1 (5.3%)
- Rhabdomyolysis	1	1 (5.3%)	0	0 (0.0%)	0	0 (0.0%)	1	1 (5.3%)
- Rotator cuff syndrome	0	0 (0.0%)	1	1 (5.3%)	0	0 (0.0%)	1	1 (5.3%)
SOC: Nervous system disorders								
- Anosmia	1	1 (5.3%)	0	0 (0.0%)	0	0 (0.0%)	1	1 (5.3%)
- Convulsion	0	0 (0.0%)	1	1 (5.3%)	0	0 (0.0%)	1	1 (5.3%)
- Grand mal convulsion	0	0 (0.0%)	1	1 (5.3%)	0	0 (0.0%)	1	1 (5.3%)
- Headache	1	1 (5.3%)	0	0 (0.0%)	0	0 (0.0%)	1	1 (5.3%)
- Pleocytosis	1	1 (5.3%)	0	0 (0.0%)	0	0 (0.0%)	1	1 (5.3%)
SOC: Psychiatric disorders								
- Depression	0	0 (0.0%)	1	1 (5.3%)	0	0 (0.0%)	1	1 (5.3%)
- Insomnia	1	1 (5.3%)	0	0 (0.0%)	0	0 (0.0%)	1	1 (5.3%)
SOC: Renal and urinary disorders								
- Renal failure	0	0 (0.0%)	0	0 (0.0%)	1	1 (5.3%)	1	1 (5.3%)
SOC: Respiratory_ thoracic and mediastinal disorders								
- Hiccups	1	1 (5.3%)	0	0 (0.0%)	0	0 (0.0%)	1	1 (5.3%)
- Pulmonary embolism	0	0 (0.0%)	1	1 (5.3%)	0	0 (0.0%)	1	1 (5.3%)

	mild		moderate		severe		Total	
	#AE	# patients with AE	#AE	# patients with AE	#AE	# patients with AE	#AE	# patients with AE
SOC: Skin and subcutaneous tissue disorders								
- Drug eruption	1	1 (5.3%)	0	0 (0.0%)	0	0 (0.0%)	1	1 (5.3%)
- Erythema	1	1 (5.3%)	0	0 (0.0%)	0	0 (0.0%)	1	1 (5.3%)
- Rash	1	1 (5.3%)	0	0 (0.0%)	0	0 (0.0%)	1	1 (5.3%)
- Skin necrosis	0	0 (0.0%)	1	1 (5.3%)	0	0 (0.0%)	1	1 (5.3%)
SOC: Surgical and medical procedures								
- Endotracheal intubation	0	0 (0.0%)	0	0 (0.0%)	1	1 (5.3%)	1	1 (5.3%)
- Gastrostomy	0	0 (0.0%)	1	1 (5.3%)	0	0 (0.0%)	1	1 (5.3%)
- Tracheostomy	0	0 (0.0%)	1	1 (5.3%)	0	0 (0.0%)	1	1 (5.3%)
SOC: Vascular disorders								
- Deep vein thrombosis	0	0 (0.0%)	1	1 (5.3%)	0	0 (0.0%)	1	1 (5.3%)
- Haematoma	1	1 (5.3%)	0	0 (0.0%)	0	0 (0.0%)	1	1 (5.3%)
- Hypertension	0	0 (0.0%)	1	1 (5.3%)	0	0 (0.0%)	1	1 (5.3%)
- Thrombosis	0	0 (0.0%)	0	0 (0.0%)	1	1 (5.3%)	1	1 (5.3%)

Table 10.6.15: SOC/PT according to severity - group: Dexamethasone

	mild		moderate		severe		Total	
	#AE	# patients with AE	#AE	# patients with AE	#AE	# patients with AE	#AE	# patients with AE
SOC: Blood and lymphatic system disorders								
- Leukocytosis	0	0 (0.0%)	1	1 (5.3%)	0	0 (0.0%)	1	1 (5.3%)
- Lymphadenopathy	1	1 (5.3%)	0	0 (0.0%)	0	0 (0.0%)	1	1 (5.3%)
SOC: Cardiac disorders								
- Arrhythmia	1	1 (5.3%)	0	0 (0.0%)	0	0 (0.0%)	1	1 (5.3%)
SOC: Ear and labyrinth disorders								
- Tinnitus	1	1 (5.3%)	0	0 (0.0%)	0	0 (0.0%)	1	1 (5.3%)
SOC: Endocrine disorders								
- Diabetes insipidus	0	0 (0.0%)	1	1 (5.3%)	0	0 (0.0%)	1	1 (5.3%)
SOC: Eye disorders								
- Amblyopia	1	1 (5.3%)	0	0 (0.0%)	0	0 (0.0%)	1	1 (5.3%)
- Diplopia	1	1 (5.3%)	0	0 (0.0%)	0	0 (0.0%)	1	1 (5.3%)
- Eye movement disorder	1	1 (5.3%)	0	0 (0.0%)	0	0 (0.0%)	1	1 (5.3%)
SOC: General disorders and administration site conditions								
- Fatigue	2	2 (10.5%)	1	1 (5.3%)	0	0 (0.0%)	3	3 (15.8%)
- Inflammation	1	1 (5.3%)	0	0 (0.0%)	0	0 (0.0%)	1	1 (5.3%)
- Pain	1	1 (5.3%)	0	0 (0.0%)	0	0 (0.0%)	1	1 (5.3%)
- Pyrexia	2	2 (10.5%)	1	1 (5.3%)	0	0 (0.0%)	3	3 (15.8%)
SOC: Infections and infestations								
- Clostridium difficile colitis	0	0 (0.0%)	1	1 (5.3%)	0	0 (0.0%)	1	1 (5.3%)
- Gastroenteritis rotavirus	0	0 (0.0%)	1	1 (5.3%)	0	0 (0.0%)	1	1 (5.3%)
- Herpes simplex meningoencephalitis	0	0 (0.0%)	0	0 (0.0%)	1	1 (5.3%)	1	1 (5.3%)
- Herpes zoster	0	0 (0.0%)	1	1 (5.3%)	0	0 (0.0%)	1	1 (5.3%)
- Pneumonia	0	0 (0.0%)	0	0 (0.0%)	1	1 (5.3%)	1	1 (5.3%)
SOC: Injury_ poisoning and procedural complications								
- Fall	0	0 (0.0%)	1	1 (5.3%)	0	0 (0.0%)	1	1 (5.3%)
- Mouth injury	0	0 (0.0%)	1	1 (5.3%)	0	0 (0.0%)	1	1 (5.3%)

	mild		moderate		severe		Total	
	#AE	# patients with AE	#AE	# patients with AE	#AE	# patients with AE	#AE	# patients with AE
SOC: Investigations								
- Blood creatinine increased	1	1 (5.3%)	0	0 (0.0%)	0	0 (0.0%)	1	1 (5.3%)
- Blood potassium decreased	1	1 (5.3%)	0	0 (0.0%)	0	0 (0.0%)	1	1 (5.3%)
- Metyrapone challenge test	1	1 (5.3%)	0	0 (0.0%)	0	0 (0.0%)	1	1 (5.3%)
SOC: Metabolism and nutrition disorders								
- Hyperglycaemia	1	1 (5.3%)	0	0 (0.0%)	0	0 (0.0%)	1	1 (5.3%)
SOC: Musculoskeletal and connective tissue disorders								
- Back pain	0	0 (0.0%)	1	1 (5.3%)	0	0 (0.0%)	1	1 (5.3%)
- Pain in extremity	1	1 (5.3%)	1	1 (5.3%)	0	0 (0.0%)	2	1 (5.3%)
SOC: Nervous system disorders								
- Aphasia	1	1 (5.3%)	0	0 (0.0%)	0	0 (0.0%)	1	1 (5.3%)
- Convulsion	0	0 (0.0%)	2	2 (10.5%)	1	1 (5.3%)	3	3 (15.8%)
- Epileptic aura	1	1 (5.3%)	0	0 (0.0%)	0	0 (0.0%)	1	1 (5.3%)
- Headache	2	2 (10.5%)	0	0 (0.0%)	0	0 (0.0%)	2	2 (10.5%)
- Memory impairment	0	0 (0.0%)	1	1 (5.3%)	0	0 (0.0%)	1	1 (5.3%)
SOC: Psychiatric disorders								
- Depressed mood	1	1 (5.3%)	0	0 (0.0%)	0	0 (0.0%)	1	1 (5.3%)
- Insomnia	1	1 (5.3%)	0	0 (0.0%)	0	0 (0.0%)	1	1 (5.3%)
SOC: Renal and urinary disorders								
- Renal failure	1	1 (5.3%)	0	0 (0.0%)	1	1 (5.3%)	2	2 (10.5%)
SOC: Respiratory_ thoracic and mediastinal disorders								
- Pneumonia aspiration	0	0 (0.0%)	0	0 (0.0%)	1	1 (5.3%)	1	1 (5.3%)
- Respiratory failure	0	0 (0.0%)	1	1 (5.3%)	1	1 (5.3%)	2	2 (10.5%)
SOC: Skin and subcutaneous tissue disorders								
- Erythema	1	1 (5.3%)	0	0 (0.0%)	0	0 (0.0%)	1	1 (5.3%)
- Rash	0	0 (0.0%)	1	1 (5.3%)	0	0 (0.0%)	1	1 (5.3%)
SOC: Social circumstances								
- Stress at work	1	1 (5.3%)	0	0 (0.0%)	0	0 (0.0%)	1	1 (5.3%)
SOC: Surgical and medical procedures								
- Endotracheal intubation	0	0 (0.0%)	1	1 (5.3%)	0	0 (0.0%)	1	1 (5.3%)
- Mechanical ventilation	0	0 (0.0%)	1	1 (5.3%)	0	0 (0.0%)	1	1 (5.3%)

	mild		moderate		severe		Total	
	#AE	# patients with AE	#AE	# patients with AE	#AE	# patients with AE	#AE	# patients with AE
SOC: Vascular disorders								
- Flushing	1	1 (5.3%)	0	0 (0.0%)	0	0 (0.0%)	1	1 (5.3%)
- Hypertension	1	1 (5.3%)	0	0 (0.0%)	0	0 (0.0%)	1	1 (5.3%)

10.6.2.2 Adverse events according to causality to study drug

Table 10.6.16: System Organ Class (SOC) according to causality to study drug - group: Placebo

	none		unlikely		possible		not assessable		Total	
	#AE	# patients with AE	#AE	# patients with AE	#AE	# patients with AE	#AE	# patients with AE	#AE	# patients with AE
System organ class										
- Cardiac disorders	1	1 (5.3%)	0	0 (0.0%)	0	0 (0.0%)	0	0 (0.0%)	1	1 (5.3%)
- Congenital, familial and genetic disorders	1	1 (5.3%)	0	0 (0.0%)	0	0 (0.0%)	0	0 (0.0%)	1	1 (5.3%)
- Eye disorders	0	0 (0.0%)	1	1 (5.3%)	0	0 (0.0%)	0	0 (0.0%)	1	1 (5.3%)
- Gastrointestinal disorders	3	2 (10.5%)	0	0 (0.0%)	0	0 (0.0%)	2	1 (5.3%)	5	3 (15.8%)
- General disorders and administration site conditions	9	7 (36.8%)	3	1 (5.3%)	1	1 (5.3%)	0	0 (0.0%)	13	7 (36.8%)
- Hepatobiliary disorders	1	1 (5.3%)	0	0 (0.0%)	0	0 (0.0%)	0	0 (0.0%)	1	1 (5.3%)
- Infections and infestations	12	8 (42.1%)	1	1 (5.3%)	0	0 (0.0%)	0	0 (0.0%)	13	9 (47.4%)
- Injury, poisoning and procedural complications	2	2 (10.5%)	0	0 (0.0%)	0	0 (0.0%)	0	0 (0.0%)	2	2 (10.5%)
- Investigations	7	4 (21.1%)	1	1 (5.3%)	0	0 (0.0%)	0	0 (0.0%)	8	5 (26.3%)
- Metabolism and nutrition disorders	3	3 (15.8%)	0	0 (0.0%)	0	0 (0.0%)	0	0 (0.0%)	3	3 (15.8%)
- Musculoskeletal and connective tissue disorders	1	1 (5.3%)	2	2 (10.5%)	0	0 (0.0%)	0	0 (0.0%)	3	3 (15.8%)
- Nervous system disorders	4	4 (21.1%)	1	1 (5.3%)	0	0 (0.0%)	0	0 (0.0%)	5	5 (26.3%)
- Psychiatric disorders	2	2 (10.5%)	0	0 (0.0%)	0	0 (0.0%)	0	0 (0.0%)	2	2 (10.5%)
- Renal and urinary disorders	1	1 (5.3%)	0	0 (0.0%)	0	0 (0.0%)	0	0 (0.0%)	1	1 (5.3%)
- Respiratory, thoracic and mediastinal disorders	2	2 (10.5%)	0	0 (0.0%)	0	0 (0.0%)	0	0 (0.0%)	2	2 (10.5%)
- Skin and subcutaneous tissue disorders	3	3 (15.8%)	0	0 (0.0%)	1	1 (5.3%)	0	0 (0.0%)	4	4 (21.1%)
- Surgical and medical procedures	3	1 (5.3%)	0	0 (0.0%)	0	0 (0.0%)	0	0 (0.0%)	3	1 (5.3%)
- Vascular disorders	3	3 (15.8%)	1	1 (5.3%)	0	0 (0.0%)	0	0 (0.0%)	4	4 (21.1%)

Table 10.6.17: System Organ Class (SOC) according to causality to study drug - group: Dexamethasone

	none		unlikely		possible		probable		not assessable		Total	
	#AE	# patients with AE	#AE	# patients with AE	#AE	# patients with AE	#AE	# patients with AE	#AE	# patients with AE	#AE	# patients with AE
System organ class												
- Blood and lymphatic system disorders	1	1 (5.3%)	0	0 (0.0%)	1	1 (5.3%)	0	0 (0.0%)	0	0 (0.0%)	2	2 (10.5%)
- Cardiac disorders	1	1 (5.3%)	0	0 (0.0%)	0	0 (0.0%)	0	0 (0.0%)	0	0 (0.0%)	1	1 (5.3%)
- Ear and labyrinth disorders	1	1 (5.3%)	0	0 (0.0%)	0	0 (0.0%)	0	0 (0.0%)	0	0 (0.0%)	1	1 (5.3%)
- Endocrine disorders	0	0 (0.0%)	1	1 (5.3%)	0	0 (0.0%)	0	0 (0.0%)	0	0 (0.0%)	1	1 (5.3%)
- Eye disorders	3	3 (15.8%)	0	0 (0.0%)	0	0 (0.0%)	0	0 (0.0%)	0	0 (0.0%)	3	3 (15.8%)
- General disorders and administration site conditions	8	7 (36.8%)	0	0 (0.0%)	0	0 (0.0%)	0	0 (0.0%)	0	0 (0.0%)	8	7 (36.8%)
- Infections and infestations	3	3 (15.8%)	1	1 (5.3%)	0	0 (0.0%)	0	0 (0.0%)	1	1 (5.3%)	5	5 (26.3%)
- Injury, poisoning and procedural complications	2	1 (5.3%)	0	0 (0.0%)	0	0 (0.0%)	0	0 (0.0%)	0	0 (0.0%)	2	1 (5.3%)
- Investigations	2	2 (10.5%)	1	1 (5.3%)	0	0 (0.0%)	0	0 (0.0%)	0	0 (0.0%)	3	2 (10.5%)
- Metabolism and nutrition disorders	0	0 (0.0%)	0	0 (0.0%)	0	0 (0.0%)	1	1 (5.3%)	0	0 (0.0%)	1	1 (5.3%)
- Musculoskeletal and connective tissue disorders	3	1 (5.3%)	0	0 (0.0%)	0	0 (0.0%)	0	0 (0.0%)	0	0 (0.0%)	3	1 (5.3%)
- Nervous system disorders	7	5 (26.3%)	1	1 (5.3%)	0	0 (0.0%)	0	0 (0.0%)	0	0 (0.0%)	8	6 (31.6%)
- Psychiatric disorders	1	1 (5.3%)	0	0 (0.0%)	1	1 (5.3%)	0	0 (0.0%)	0	0 (0.0%)	2	2 (10.5%)
- Renal and urinary disorders	1	1 (5.3%)	1	1 (5.3%)	0	0 (0.0%)	0	0 (0.0%)	0	0 (0.0%)	2	2 (10.5%)
- Respiratory, thoracic and mediastinal disorders	2	2 (10.5%)	1	1 (5.3%)	0	0 (0.0%)	0	0 (0.0%)	0	0 (0.0%)	3	3 (15.8%)
- Skin and subcutaneous tissue disorders	1	1 (5.3%)	1	1 (5.3%)	0	0 (0.0%)	0	0 (0.0%)	0	0 (0.0%)	2	2 (10.5%)
- Social circumstances	1	1 (5.3%)	0	0 (0.0%)	0	0 (0.0%)	0	0 (0.0%)	0	0 (0.0%)	1	1 (5.3%)
- Surgical and medical procedures	0	0 (0.0%)	2	1 (5.3%)	0	0 (0.0%)	0	0 (0.0%)	0	0 (0.0%)	2	1 (5.3%)
- Vascular disorders	1	1 (5.3%)	0	0 (0.0%)	1	1 (5.3%)	0	0 (0.0%)	0	0 (0.0%)	2	2 (10.5%)

Table 10.6.18: SOC / PT according to causality to study drug - group: Placebo

	ongoing		recovered completely		recovered with sequelae		death		unknown		Total	
	#AE	# patients with AE	#AE	# patients with AE	#AE	# patients with AE	#AE	# patients with AE	#AE	# patients with AE	#AE	# patients with AE
SOC: Cardiac disorders												
- Atrial fibrillation	0	0 (0.0%)	0	0 (0.0%)	0	0 (0.0%)	0	0 (0.0%)	0	0 (0.0%)	1	1 (5.3%)
SOC: Congenital_ familial and genetic disorders												
- Hydrocele	0	0 (0.0%)	0	0 (0.0%)	0	0 (0.0%)	0	0 (0.0%)	0	0 (0.0%)	1	1 (5.3%)
SOC: Eye disorders												
- Visual impairment	1	1 (5.3%)	0	0 (0.0%)	0	0 (0.0%)	0	0 (0.0%)	0	0 (0.0%)	1	1 (5.3%)
SOC: Gastrointestinal disorders												
- Constipation	0	0 (0.0%)	0	0 (0.0%)	0	0 (0.0%)	0	0 (0.0%)	0	0 (0.0%)	1	1 (5.3%)
- Diarrhoea	0	0 (0.0%)	0	0 (0.0%)	0	0 (0.0%)	0	0 (0.0%)	0	0 (0.0%)	1	1 (5.3%)
- Nausea	0	0 (0.0%)	0	0 (0.0%)	1	1 (5.3%)	0	0 (0.0%)	0	0 (0.0%)	1	1 (5.3%)
- Vomiting	0	0 (0.0%)	0	0 (0.0%)	1	1 (5.3%)	0	0 (0.0%)	0	0 (0.0%)	2	2 (10.5%)
SOC: General disorders and administration site conditions												
- Condition aggravated	0	0 (0.0%)	0	0 (0.0%)	0	0 (0.0%)	0	0 (0.0%)	0	0 (0.0%)	1	1 (5.3%)
- General physical health deterioration	0	0 (0.0%)	0	0 (0.0%)	0	0 (0.0%)	0	0 (0.0%)	0	0 (0.0%)	1	1 (5.3%)
- Infusion site extravasation	0	0 (0.0%)	0	0 (0.0%)	0	0 (0.0%)	0	0 (0.0%)	0	0 (0.0%)	1	1 (5.3%)
- Local swelling	0	0 (0.0%)	0	0 (0.0%)	0	0 (0.0%)	0	0 (0.0%)	0	0 (0.0%)	1	1 (5.3%)
- Oedema peripheral	0	0 (0.0%)	0	0 (0.0%)	0	0 (0.0%)	0	0 (0.0%)	0	0 (0.0%)	1	1 (5.3%)
- Pain	0	0 (0.0%)	0	0 (0.0%)	0	0 (0.0%)	0	0 (0.0%)	0	0 (0.0%)	1	1 (5.3%)
- Pyrexia	3	1 (5.3%)	1	1 (5.3%)	0	0 (0.0%)	0	0 (0.0%)	0	0 (0.0%)	7	4 (21.1%)
SOC: Hepatobiliary disorders												
- Cholangitis	0	0 (0.0%)	0	0 (0.0%)	0	0 (0.0%)	0	0 (0.0%)	0	0 (0.0%)	1	1 (5.3%)
SOC: Infections and infestations												
- Abscess	0	0 (0.0%)	0	0 (0.0%)	0	0 (0.0%)	0	0 (0.0%)	0	0 (0.0%)	1	1 (5.3%)
- Bacteraemia	1	1 (5.3%)	0	0 (0.0%)	0	0 (0.0%)	0	0 (0.0%)	0	0 (0.0%)	1	1 (5.3%)
- Bacterial infection	0	0 (0.0%)	0	0 (0.0%)	0	0 (0.0%)	0	0 (0.0%)	0	0 (0.0%)	1	1 (5.3%)
- Brain abscess	0	0 (0.0%)	0	0 (0.0%)	0	0 (0.0%)	0	0 (0.0%)	0	0 (0.0%)	1	1 (5.3%)
- Bronchitis	0	0 (0.0%)	0	0 (0.0%)	0	0 (0.0%)	0	0 (0.0%)	0	0 (0.0%)	1	1 (5.3%)
- Bronchopneumonia	0	0 (0.0%)	0	0 (0.0%)	0	0 (0.0%)	0	0 (0.0%)	0	0 (0.0%)	2	1 (5.3%)
- Herpes simplex	0	0 (0.0%)	0	0 (0.0%)	0	0 (0.0%)	0	0 (0.0%)	0	0 (0.0%)	2	2 (10.5%)
- Pneumonia	0	0 (0.0%)	0	0 (0.0%)	0	0 (0.0%)	0	0 (0.0%)	0	0 (0.0%)	1	1 (5.3%)
- Sepsis	0	0 (0.0%)	0	0 (0.0%)	0	0 (0.0%)	0	0 (0.0%)	0	0 (0.0%)	2	2 (10.5%)
- Urinary tract infection	0	0 (0.0%)	0	0 (0.0%)	0	0 (0.0%)	0	0 (0.0%)	0	0 (0.0%)	1	1 (5.3%)

	ongoing		recovered completely		recovered with sequelae		death		unknown		Total	
	#AE	# patients with AE	#AE	# patients with AE	#AE	# patients with AE	#AE	# patients with AE	#AE	# patients with AE	#AE	# patients with AE
SOC: Injury_ poisoning and procedural complications												
- Fall	0	0 (0.0%)	0	0 (0.0%)	0	0 (0.0%)	0	0 (0.0%)	0	0 (0.0%)	2	2 (10.5%)
SOC: Investigations												
- Alanine aminotransferase increased	0	0 (0.0%)	0	0 (0.0%)	0	0 (0.0%)	0	0 (0.0%)	0	0 (0.0%)	1	1 (5.3%)
- Blood acid phosphatase increased	0	0 (0.0%)	0	0 (0.0%)	0	0 (0.0%)	0	0 (0.0%)	0	0 (0.0%)	1	1 (5.3%)
- Blood creatinine increased	0	0 (0.0%)	0	0 (0.0%)	0	0 (0.0%)	0	0 (0.0%)	0	0 (0.0%)	1	1 (5.3%)
- Blood culture positive	1	1 (5.3%)	0	0 (0.0%)	0	0 (0.0%)	0	0 (0.0%)	0	0 (0.0%)	1	1 (5.3%)
- Body temperature increased	0	0 (0.0%)	0	0 (0.0%)	0	0 (0.0%)	0	0 (0.0%)	0	0 (0.0%)	1	1 (5.3%)
- C-reactive protein increased	0	0 (0.0%)	0	0 (0.0%)	0	0 (0.0%)	0	0 (0.0%)	0	0 (0.0%)	1	1 (5.3%)
- Lipase increased	0	0 (0.0%)	0	0 (0.0%)	0	0 (0.0%)	0	0 (0.0%)	0	0 (0.0%)	1	1 (5.3%)
- Weight decreased	0	0 (0.0%)	0	0 (0.0%)	0	0 (0.0%)	0	0 (0.0%)	0	0 (0.0%)	1	1 (5.3%)
SOC: Metabolism and nutrition disorders												
- Decreased appetite	0	0 (0.0%)	0	0 (0.0%)	0	0 (0.0%)	0	0 (0.0%)	0	0 (0.0%)	1	1 (5.3%)
- Hypokalaemia	0	0 (0.0%)	0	0 (0.0%)	0	0 (0.0%)	0	0 (0.0%)	0	0 (0.0%)	2	2 (10.5%)
SOC: Musculoskeletal and connective tissue disorders												
- Muscle contracture	0	0 (0.0%)	0	0 (0.0%)	0	0 (0.0%)	0	0 (0.0%)	0	0 (0.0%)	1	1 (5.3%)
- Rhabdomyolysis	1	1 (5.3%)	0	0 (0.0%)	0	0 (0.0%)	0	0 (0.0%)	0	0 (0.0%)	1	1 (5.3%)
- Rotator cuff syndrome	1	1 (5.3%)	0	0 (0.0%)	0	0 (0.0%)	0	0 (0.0%)	0	0 (0.0%)	1	1 (5.3%)
SOC: Nervous system disorders												
- Anosmia	0	0 (0.0%)	0	0 (0.0%)	0	0 (0.0%)	0	0 (0.0%)	0	0 (0.0%)	1	1 (5.3%)
- Convulsion	0	0 (0.0%)	0	0 (0.0%)	0	0 (0.0%)	0	0 (0.0%)	0	0 (0.0%)	1	1 (5.3%)
- Grand mal convulsion	1	1 (5.3%)	0	0 (0.0%)	0	0 (0.0%)	0	0 (0.0%)	0	0 (0.0%)	1	1 (5.3%)
- Headache	0	0 (0.0%)	0	0 (0.0%)	0	0 (0.0%)	0	0 (0.0%)	0	0 (0.0%)	1	1 (5.3%)
- Pleocytosis	0	0 (0.0%)	0	0 (0.0%)	0	0 (0.0%)	0	0 (0.0%)	0	0 (0.0%)	1	1 (5.3%)
SOC: Psychiatric disorders												
- Depression	0	0 (0.0%)	0	0 (0.0%)	0	0 (0.0%)	0	0 (0.0%)	0	0 (0.0%)	1	1 (5.3%)
- Insomnia	0	0 (0.0%)	0	0 (0.0%)	0	0 (0.0%)	0	0 (0.0%)	0	0 (0.0%)	1	1 (5.3%)
SOC: Renal and urinary disorders												
- Renal failure	0	0 (0.0%)	0	0 (0.0%)	0	0 (0.0%)	0	0 (0.0%)	0	0 (0.0%)	1	1 (5.3%)

	ongoing		recovered completely		recovered with sequelae		death		unknown		Total	
	#AE	# patients with AE	#AE	# patients with AE	#AE	# patients with AE	#AE	# patients with AE	#AE	# patients with AE	#AE	# patients with AE
SOC: Respiratory_ thoracic and mediastinal disorders												
- Hiccups	0	0 (0.0%)	0	0 (0.0%)	0	0 (0.0%)	0	0 (0.0%)	0	0 (0.0%)	1	1 (5.3%)
- Pulmonary embolism	0	0 (0.0%)	0	0 (0.0%)	0	0 (0.0%)	0	0 (0.0%)	0	0 (0.0%)	1	1 (5.3%)
SOC: Skin and subcutaneous tissue disorders												
- Drug eruption	0	0 (0.0%)	0	0 (0.0%)	0	0 (0.0%)	0	0 (0.0%)	0	0 (0.0%)	1	1 (5.3%)
- Erythema	0	0 (0.0%)	1	1 (5.3%)	0	0 (0.0%)	0	0 (0.0%)	0	0 (0.0%)	1	1 (5.3%)
- Rash	0	0 (0.0%)	0	0 (0.0%)	0	0 (0.0%)	0	0 (0.0%)	0	0 (0.0%)	1	1 (5.3%)
- Skin necrosis	0	0 (0.0%)	0	0 (0.0%)	0	0 (0.0%)	0	0 (0.0%)	0	0 (0.0%)	1	1 (5.3%)
SOC: Surgical and medical procedures												
- Endotracheal intubation	0	0 (0.0%)	0	0 (0.0%)	0	0 (0.0%)	0	0 (0.0%)	0	0 (0.0%)	1	1 (5.3%)
- Gastrostomy	0	0 (0.0%)	0	0 (0.0%)	0	0 (0.0%)	0	0 (0.0%)	0	0 (0.0%)	1	1 (5.3%)
- Tracheostomy	0	0 (0.0%)	0	0 (0.0%)	0	0 (0.0%)	0	0 (0.0%)	0	0 (0.0%)	1	1 (5.3%)
SOC: Vascular disorders												
- Deep vein thrombosis	0	0 (0.0%)	0	0 (0.0%)	0	0 (0.0%)	0	0 (0.0%)	0	0 (0.0%)	1	1 (5.3%)
- Haematoma	0	0 (0.0%)	0	0 (0.0%)	0	0 (0.0%)	0	0 (0.0%)	0	0 (0.0%)	1	1 (5.3%)
- Hypertension	1	1 (5.3%)	0	0 (0.0%)	0	0 (0.0%)	0	0 (0.0%)	0	0 (0.0%)	1	1 (5.3%)
- Thrombosis	0	0 (0.0%)	0	0 (0.0%)	0	0 (0.0%)	0	0 (0.0%)	0	0 (0.0%)	1	1 (5.3%)

Table 10.6.19: SOC / PT according to causality to study drug - group: Dexamethasone

	ongoing		recovered completely		recovered with sequelae		death		unknown		Total	
	#AE	# patients with AE	#AE	# patients with AE	#AE	# patients with AE	#AE	# patients with AE	#AE	# patients with AE	#AE	# patients with AE
SOC: Blood and lymphatic system disorders												
- Leukocytosis	0	0 (0.0%)	1	1 (5.3%)	0	0 (0.0%)	0	0 (0.0%)	0	0 (0.0%)	1	1 (5.3%)
- Lymphadenopathy	0	0 (0.0%)	0	0 (0.0%)	0	0 (0.0%)	0	0 (0.0%)	0	0 (0.0%)	1	1 (5.3%)
SOC: Cardiac disorders												
- Arrhythmia	0	0 (0.0%)	0	0 (0.0%)	0	0 (0.0%)	0	0 (0.0%)	0	0 (0.0%)	1	1 (5.3%)
SOC: Ear and labyrinth disorders												
- Tinnitus	0	0 (0.0%)	0	0 (0.0%)	0	0 (0.0%)	0	0 (0.0%)	0	0 (0.0%)	1	1 (5.3%)
SOC: Endocrine disorders												
- Diabetes insipidus	1	1 (5.3%)	0	0 (0.0%)	0	0 (0.0%)	0	0 (0.0%)	0	0 (0.0%)	1	1 (5.3%)
SOC: Eye disorders												
- Amblyopia	0	0 (0.0%)	0	0 (0.0%)	0	0 (0.0%)	0	0 (0.0%)	0	0 (0.0%)	1	1 (5.3%)
- Diplopia	0	0 (0.0%)	0	0 (0.0%)	0	0 (0.0%)	0	0 (0.0%)	0	0 (0.0%)	1	1 (5.3%)
- Eye movement disorder	0	0 (0.0%)	0	0 (0.0%)	0	0 (0.0%)	0	0 (0.0%)	0	0 (0.0%)	1	1 (5.3%)
SOC: General disorders and administration site conditions												
- Fatigue	0	0 (0.0%)	0	0 (0.0%)	0	0 (0.0%)	0	0 (0.0%)	0	0 (0.0%)	3	3 (15.8%)
- Inflammation	0	0 (0.0%)	0	0 (0.0%)	0	0 (0.0%)	0	0 (0.0%)	0	0 (0.0%)	1	1 (5.3%)
- Pain	0	0 (0.0%)	0	0 (0.0%)	0	0 (0.0%)	0	0 (0.0%)	0	0 (0.0%)	1	1 (5.3%)
- Pyrexia	0	0 (0.0%)	0	0 (0.0%)	0	0 (0.0%)	0	0 (0.0%)	0	0 (0.0%)	3	3 (15.8%)
SOC: Infections and infestations												
- Clostridium difficile colitis	1	1 (5.3%)	0	0 (0.0%)	0	0 (0.0%)	0	0 (0.0%)	0	0 (0.0%)	1	1 (5.3%)
- Gastroenteritis rotavirus	0	0 (0.0%)	0	0 (0.0%)	0	0 (0.0%)	0	0 (0.0%)	0	0 (0.0%)	1	1 (5.3%)
- Herpes simplex meningoencephalitis	0	0 (0.0%)	0	0 (0.0%)	0	0 (0.0%)	1	1 (5.3%)	0	0 (0.0%)	1	1 (5.3%)
- Herpes zoster	0	0 (0.0%)	0	0 (0.0%)	0	0 (0.0%)	0	0 (0.0%)	0	0 (0.0%)	1	1 (5.3%)
- Pneumonia	0	0 (0.0%)	0	0 (0.0%)	0	0 (0.0%)	0	0 (0.0%)	0	0 (0.0%)	1	1 (5.3%)
SOC: Injury_ poisoning and procedural complications												
- Fall	0	0 (0.0%)	0	0 (0.0%)	0	0 (0.0%)	0	0 (0.0%)	0	0 (0.0%)	1	1 (5.3%)
- Mouth injury	0	0 (0.0%)	0	0 (0.0%)	0	0 (0.0%)	0	0 (0.0%)	0	0 (0.0%)	1	1 (5.3%)

	ongoing		recovered completely		recovered with sequelae		death		unknown		Total	
	#AE	# patients with AE	#AE	# patients with AE	#AE	# patients with AE	#AE	# patients with AE	#AE	# patients with AE	#AE	# patients with AE
SOC: Investigations												
- Blood creatinine increased	1	1 (5.3%)	0	0 (0.0%)	0	0 (0.0%)	0	0 (0.0%)	0	0 (0.0%)	1	1 (5.3%)
- Blood potassium decreased	0	0 (0.0%)	0	0 (0.0%)	0	0 (0.0%)	0	0 (0.0%)	0	0 (0.0%)	1	1 (5.3%)
- Metyrapone challenge test	0	0 (0.0%)	0	0 (0.0%)	0	0 (0.0%)	0	0 (0.0%)	0	0 (0.0%)	1	1 (5.3%)
SOC: Metabolism and nutrition disorders												
- Hyperglycaemia	0	0 (0.0%)	0	0 (0.0%)	1	1 (5.3%)	0	0 (0.0%)	0	0 (0.0%)	1	1 (5.3%)
SOC: Musculoskeletal and connective tissue disorders												
- Back pain	0	0 (0.0%)	0	0 (0.0%)	0	0 (0.0%)	0	0 (0.0%)	0	0 (0.0%)	1	1 (5.3%)
- Pain in extremity	0	0 (0.0%)	0	0 (0.0%)	0	0 (0.0%)	0	0 (0.0%)	0	0 (0.0%)	2	1 (5.3%)
SOC: Nervous system disorders												
- Aphasia	0	0 (0.0%)	0	0 (0.0%)	0	0 (0.0%)	0	0 (0.0%)	0	0 (0.0%)	1	1 (5.3%)
- Convulsion	1	1 (5.3%)	0	0 (0.0%)	0	0 (0.0%)	0	0 (0.0%)	0	0 (0.0%)	3	3 (15.8%)
- Epileptic aura	0	0 (0.0%)	0	0 (0.0%)	0	0 (0.0%)	0	0 (0.0%)	0	0 (0.0%)	1	1 (5.3%)
- Headache	0	0 (0.0%)	0	0 (0.0%)	0	0 (0.0%)	0	0 (0.0%)	0	0 (0.0%)	2	2 (10.5%)
- Memory impairment	0	0 (0.0%)	0	0 (0.0%)	0	0 (0.0%)	0	0 (0.0%)	0	0 (0.0%)	1	1 (5.3%)
SOC: Psychiatric disorders												
- Depressed mood	0	0 (0.0%)	1	1 (5.3%)	0	0 (0.0%)	0	0 (0.0%)	0	0 (0.0%)	1	1 (5.3%)
- Insomnia	0	0 (0.0%)	0	0 (0.0%)	0	0 (0.0%)	0	0 (0.0%)	0	0 (0.0%)	1	1 (5.3%)
SOC: Renal and urinary disorders												
- Renal failure	1	1 (5.3%)	0	0 (0.0%)	0	0 (0.0%)	0	0 (0.0%)	0	0 (0.0%)	2	2 (10.5%)
SOC: Respiratory_ thoracic and mediastinal disorders												
- Pneumonia aspiration	0	0 (0.0%)	0	0 (0.0%)	0	0 (0.0%)	0	0 (0.0%)	0	0 (0.0%)	1	1 (5.3%)
- Respiratory failure	1	1 (5.3%)	0	0 (0.0%)	0	0 (0.0%)	0	0 (0.0%)	0	0 (0.0%)	2	2 (10.5%)
SOC: Skin and subcutaneous tissue disorders												
- Erythema	1	1 (5.3%)	0	0 (0.0%)	0	0 (0.0%)	0	0 (0.0%)	0	0 (0.0%)	1	1 (5.3%)
- Rash	0	0 (0.0%)	0	0 (0.0%)	0	0 (0.0%)	0	0 (0.0%)	0	0 (0.0%)	1	1 (5.3%)
SOC: Social circumstances												
- Stress at work	0	0 (0.0%)	0	0 (0.0%)	0	0 (0.0%)	0	0 (0.0%)	0	0 (0.0%)	1	1 (5.3%)

	ongoing		recovered completely		recovered with sequelae		death		unknown		Total	
	#AE	# patients with AE	#AE	# patients with AE	#AE	# patients with AE	#AE	# patients with AE	#AE	# patients with AE	#AE	# patients with AE
SOC: Surgical and medical procedures												
- Endotracheal intubation	1	1 (5.3%)	0	0 (0.0%)	0	0 (0.0%)	0	0 (0.0%)	0	0 (0.0%)	1	1 (5.3%)
- Mechanical ventilation	1	1 (5.3%)	0	0 (0.0%)	0	0 (0.0%)	0	0 (0.0%)	0	0 (0.0%)	1	1 (5.3%)
SOC: Vascular disorders												
- Flushing	0	0 (0.0%)	1	1 (5.3%)	0	0 (0.0%)	0	0 (0.0%)	0	0 (0.0%)	1	1 (5.3%)
- Hypertension	0	0 (0.0%)	0	0 (0.0%)	0	0 (0.0%)	0	0 (0.0%)	0	0 (0.0%)	1	1 (5.3%)

10.6.2.3 Adverse events according to outcome

Table 10.6.20: System Organ Class (SOC) according to outcome - group: Placebo

	ongoing		recovered completely		recovered with sequelae		death		unknown		Total	
	#AE	# patients with AE	#AE	# patients with AE	#AE	# patients with AE	#AE	# patients with AE	#AE	# patients with AE	#AE	# patients with AE
System organ class												
- Cardiac disorders	0	0 (0.0%)	1	1 (5.3%)	0	0 (0.0%)	0	0 (0.0%)	0	0 (0.0%)	1	1 (5.3%)
- Congenital, familial and genetic disorders	0	0 (0.0%)	0	0 (0.0%)	0	0 (0.0%)	0	0 (0.0%)	1	1 (5.3%)	1	1 (5.3%)
- Eye disorders	1	1 (5.3%)	0	0 (0.0%)	0	0 (0.0%)	0	0 (0.0%)	0	0 (0.0%)	1	1 (5.3%)
- Gastrointestinal disorders	0	0 (0.0%)	5	3 (15.8%)	0	0 (0.0%)	0	0 (0.0%)	0	0 (0.0%)	5	3 (15.8%)
- General disorders and administration site conditions	1	1 (5.3%)	11	6 (31.6%)	0	0 (0.0%)	1	1 (5.3%)	0	0 (0.0%)	13	7 (36.8%)
- Hepatobiliary disorders	0	0 (0.0%)	0	0 (0.0%)	0	0 (0.0%)	1	1 (5.3%)	0	0 (0.0%)	1	1 (5.3%)
- Infections and infestations	0	0 (0.0%)	11	8 (42.1%)	0	0 (0.0%)	2	1 (5.3%)	0	0 (0.0%)	13	9 (47.4%)
- Injury, poisoning and procedural complications	0	0 (0.0%)	2	2 (10.5%)	0	0 (0.0%)	0	0 (0.0%)	0	0 (0.0%)	2	2 (10.5%)
- Investigations	3	1 (5.3%)	5	5 (26.3%)	0	0 (0.0%)	0	0 (0.0%)	0	0 (0.0%)	8	5 (26.3%)
- Metabolism and nutrition disorders	2	2 (10.5%)	1	1 (5.3%)	0	0 (0.0%)	0	0 (0.0%)	0	0 (0.0%)	3	3 (15.8%)
- Musculoskeletal and connective tissue disorders	1	1 (5.3%)	1	1 (5.3%)	0	0 (0.0%)	0	0 (0.0%)	1	1 (5.3%)	3	3 (15.8%)
- Nervous system disorders	1	1 (5.3%)	2	2 (10.5%)	1	1 (5.3%)	0	0 (0.0%)	1	1 (5.3%)	5	5 (26.3%)
- Psychiatric disorders	2	2 (10.5%)	0	0 (0.0%)	0	0 (0.0%)	0	0 (0.0%)	0	0 (0.0%)	2	2 (10.5%)
- Renal and urinary disorders	0	0 (0.0%)	0	0 (0.0%)	0	0 (0.0%)	1	1 (5.3%)	0	0 (0.0%)	1	1 (5.3%)
- Respiratory, thoracic and mediastinal disorders	0	0 (0.0%)	2	2 (10.5%)	0	0 (0.0%)	0	0 (0.0%)	0	0 (0.0%)	2	2 (10.5%)
- Skin and subcutaneous tissue disorders	0	0 (0.0%)	4	4 (21.1%)	0	0 (0.0%)	0	0 (0.0%)	0	0 (0.0%)	4	4 (21.1%)
- Surgical and medical procedures	0	0 (0.0%)	3	1 (5.3%)	0	0 (0.0%)	0	0 (0.0%)	0	0 (0.0%)	3	1 (5.3%)
- Vascular disorders	1	1 (5.3%)	2	2 (10.5%)	0	0 (0.0%)	0	0 (0.0%)	1	1 (5.3%)	4	4 (21.1%)

Table 10.6.21: System Organ Class (SOC) according to outcome - group: Dexamethasone

	ongoing		recovered completely		recovered with sequelae		death		unknown		Total	
	#AE	# patients with AE	#AE	# patients with AE	#AE	# patients with AE	#AE	# patients with AE	#AE	# patients with AE	#AE	# patients with AE
System organ class												
- Blood and lymphatic system disorders	0	0 (0.0%)	1	1 (5.3%)	0	0 (0.0%)	0	0 (0.0%)	1	1 (5.3%)	2	2 (10.5%)
- Cardiac disorders	1	1 (5.3%)	0	0 (0.0%)	0	0 (0.0%)	0	0 (0.0%)	0	0 (0.0%)	1	1 (5.3%)
- Ear and labyrinth disorders	1	1 (5.3%)	0	0 (0.0%)	0	0 (0.0%)	0	0 (0.0%)	0	0 (0.0%)	1	1 (5.3%)
- Endocrine disorders	0	0 (0.0%)	1	1 (5.3%)	0	0 (0.0%)	0	0 (0.0%)	0	0 (0.0%)	1	1 (5.3%)
- Eye disorders	1	1 (5.3%)	2	2 (10.5%)	0	0 (0.0%)	0	0 (0.0%)	0	0 (0.0%)	3	3 (15.8%)
- General disorders and administration site conditions	4	4 (21.1%)	3	3 (15.8%)	0	0 (0.0%)	0	0 (0.0%)	1	1 (5.3%)	8	7 (36.8%)
- Infections and infestations	0	0 (0.0%)	4	4 (21.1%)	0	0 (0.0%)	0	0 (0.0%)	1	1 (5.3%)	5	5 (26.3%)
- Injury, poisoning and procedural complications	0	0 (0.0%)	2	1 (5.3%)	0	0 (0.0%)	0	0 (0.0%)	0	0 (0.0%)	2	1 (5.3%)
- Investigations	0	0 (0.0%)	3	2 (10.5%)	0	0 (0.0%)	0	0 (0.0%)	0	0 (0.0%)	3	2 (10.5%)
- Metabolism and nutrition disorders	0	0 (0.0%)	1	1 (5.3%)	0	0 (0.0%)	0	0 (0.0%)	0	0 (0.0%)	1	1 (5.3%)
- Musculoskeletal and connective tissue disorders	0	0 (0.0%)	3	1 (5.3%)	0	0 (0.0%)	0	0 (0.0%)	0	0 (0.0%)	3	1 (5.3%)
- Nervous system disorders	4	4 (21.1%)	3	3 (15.8%)	0	0 (0.0%)	0	0 (0.0%)	1	1 (5.3%)	8	6 (31.6%)
- Psychiatric disorders	2	2 (10.5%)	0	0 (0.0%)	0	0 (0.0%)	0	0 (0.0%)	0	0 (0.0%)	2	2 (10.5%)
- Renal and urinary disorders	0	0 (0.0%)	1	1 (5.3%)	1	1 (5.3%)	0	0 (0.0%)	0	0 (0.0%)	2	2 (10.5%)
- Respiratory, thoracic and mediastinal disorders	0	0 (0.0%)	1	1 (5.3%)	1	1 (5.3%)	1	1 (5.3%)	0	0 (0.0%)	3	3 (15.8%)
- Skin and subcutaneous tissue disorders	0	0 (0.0%)	2	2 (10.5%)	0	0 (0.0%)	0	0 (0.0%)	0	0 (0.0%)	2	2 (10.5%)
- Social circumstances	1	1 (5.3%)	0	0 (0.0%)	0	0 (0.0%)	0	0 (0.0%)	0	0 (0.0%)	1	1 (5.3%)
- Surgical and medical procedures	0	0 (0.0%)	0	0 (0.0%)	2	1 (5.3%)	0	0 (0.0%)	0	0 (0.0%)	2	1 (5.3%)
- Vascular disorders	0	0 (0.0%)	2	2 (10.5%)	0	0 (0.0%)	0	0 (0.0%)	0	0 (0.0%)	2	2 (10.5%)

Table 10.6.22: SOC / PT according to outcome - group: Placebo

	ongoing		recovered completely		recovered with sequelae		death		unknown		Total	
	#AE	# patients with AE	#AE	# patients with AE	#AE	# patients with AE	#AE	# patients with AE	#AE	# patients with AE	#AE	# patients with AE
SOC: Cardiac disorders												
- Atrial fibrillation	0	0 (0.0%)	1	1 (5.3%)	0	0 (0.0%)	0	0 (0.0%)	0	0 (0.0%)	1	1 (5.3%)
SOC: Congenital_ familial and genetic disorders												
- Hydrocele	0	0 (0.0%)	0	0 (0.0%)	0	0 (0.0%)	0	0 (0.0%)	1	1 (5.3%)	1	1 (5.3%)
SOC: Eye disorders												
- Visual impairment	1	1 (5.3%)	0	0 (0.0%)	0	0 (0.0%)	0	0 (0.0%)	0	0 (0.0%)	1	1 (5.3%)
SOC: Gastrointestinal disorders												
- Constipation	0	0 (0.0%)	1	1 (5.3%)	0	0 (0.0%)	0	0 (0.0%)	0	0 (0.0%)	1	1 (5.3%)
- Diarrhoea	0	0 (0.0%)	1	1 (5.3%)	0	0 (0.0%)	0	0 (0.0%)	0	0 (0.0%)	1	1 (5.3%)
- Nausea	0	0 (0.0%)	1	1 (5.3%)	0	0 (0.0%)	0	0 (0.0%)	0	0 (0.0%)	1	1 (5.3%)
- Vomiting	0	0 (0.0%)	2	2 (10.5%)	0	0 (0.0%)	0	0 (0.0%)	0	0 (0.0%)	2	2 (10.5%)
SOC: General disorders and administration site conditions												
- Condition aggravated	0	0 (0.0%)	0	0 (0.0%)	0	0 (0.0%)	1	1 (5.3%)	0	0 (0.0%)	1	1 (5.3%)
- General physical health deterioration	1	1 (5.3%)	0	0 (0.0%)	0	0 (0.0%)	0	0 (0.0%)	0	0 (0.0%)	1	1 (5.3%)
- Infusion site extravasation	0	0 (0.0%)	1	1 (5.3%)	0	0 (0.0%)	0	0 (0.0%)	0	0 (0.0%)	1	1 (5.3%)
- Local swelling	0	0 (0.0%)	1	1 (5.3%)	0	0 (0.0%)	0	0 (0.0%)	0	0 (0.0%)	1	1 (5.3%)
- Oedema peripheral	0	0 (0.0%)	1	1 (5.3%)	0	0 (0.0%)	0	0 (0.0%)	0	0 (0.0%)	1	1 (5.3%)
- Pain	0	0 (0.0%)	1	1 (5.3%)	0	0 (0.0%)	0	0 (0.0%)	0	0 (0.0%)	1	1 (5.3%)
- Pyrexia	0	0 (0.0%)	7	4 (21.1%)	0	0 (0.0%)	0	0 (0.0%)	0	0 (0.0%)	7	4 (21.1%)
SOC: Hepatobiliary disorders												
- Cholangitis	0	0 (0.0%)	0	0 (0.0%)	0	0 (0.0%)	1	1 (5.3%)	0	0 (0.0%)	1	1 (5.3%)
SOC: Infections and infestations												
- Abscess	0	0 (0.0%)	0	0 (0.0%)	0	0 (0.0%)	1	1 (5.3%)	0	0 (0.0%)	1	1 (5.3%)
- Bacteraemia	0	0 (0.0%)	1	1 (5.3%)	0	0 (0.0%)	0	0 (0.0%)	0	0 (0.0%)	1	1 (5.3%)
- Bacterial infection	0	0 (0.0%)	1	1 (5.3%)	0	0 (0.0%)	0	0 (0.0%)	0	0 (0.0%)	1	1 (5.3%)
- Brain abscess	0	0 (0.0%)	1	1 (5.3%)	0	0 (0.0%)	0	0 (0.0%)	0	0 (0.0%)	1	1 (5.3%)
- Bronchitis	0	0 (0.0%)	1	1 (5.3%)	0	0 (0.0%)	0	0 (0.0%)	0	0 (0.0%)	1	1 (5.3%)
- Bronchopneumonia	0	0 (0.0%)	2	1 (5.3%)	0	0 (0.0%)	0	0 (0.0%)	0	0 (0.0%)	2	1 (5.3%)
- Herpes simplex	0	0 (0.0%)	2	2 (10.5%)	0	0 (0.0%)	0	0 (0.0%)	0	0 (0.0%)	2	2 (10.5%)
- Pneumonia	0	0 (0.0%)	1	1 (5.3%)	0	0 (0.0%)	0	0 (0.0%)	0	0 (0.0%)	1	1 (5.3%)
- Sepsis	0	0 (0.0%)	1	1 (5.3%)	0	0 (0.0%)	1	1 (5.3%)	0	0 (0.0%)	2	2 (10.5%)
- Urinary tract infection	0	0 (0.0%)	1	1 (5.3%)	0	0 (0.0%)	0	0 (0.0%)	0	0 (0.0%)	1	1 (5.3%)

	ongoing		recovered completely		recovered with sequelae		death		unknown		Total	
	#AE	# patients with AE	#AE	# patients with AE	#AE	# patients with AE	#AE	# patients with AE	#AE	# patients with AE	#AE	# patients with AE
SOC: Injury_ poisoning and procedural complications												
- Fall	0	0 (0.0%)	2	2 (10.5%)	0	0 (0.0%)	0	0 (0.0%)	0	0 (0.0%)	2	2 (10.5%)
SOC: Investigations												
- Alanine aminotransferase increased	1	1 (5.3%)	0	0 (0.0%)	0	0 (0.0%)	0	0 (0.0%)	0	0 (0.0%)	1	1 (5.3%)
- Blood acid phosphatase increased	1	1 (5.3%)	0	0 (0.0%)	0	0 (0.0%)	0	0 (0.0%)	0	0 (0.0%)	1	1 (5.3%)
- Blood creatinine increased	0	0 (0.0%)	1	1 (5.3%)	0	0 (0.0%)	0	0 (0.0%)	0	0 (0.0%)	1	1 (5.3%)
- Blood culture positive	0	0 (0.0%)	1	1 (5.3%)	0	0 (0.0%)	0	0 (0.0%)	0	0 (0.0%)	1	1 (5.3%)
- Body temperature increased	0	0 (0.0%)	1	1 (5.3%)	0	0 (0.0%)	0	0 (0.0%)	0	0 (0.0%)	1	1 (5.3%)
- C-reactive protein increased	0	0 (0.0%)	1	1 (5.3%)	0	0 (0.0%)	0	0 (0.0%)	0	0 (0.0%)	1	1 (5.3%)
- Lipase increased	1	1 (5.3%)	0	0 (0.0%)	0	0 (0.0%)	0	0 (0.0%)	0	0 (0.0%)	1	1 (5.3%)
- Weight decreased	0	0 (0.0%)	1	1 (5.3%)	0	0 (0.0%)	0	0 (0.0%)	0	0 (0.0%)	1	1 (5.3%)
SOC: Metabolism and nutrition disorders												
- Decreased appetite	1	1 (5.3%)	0	0 (0.0%)	0	0 (0.0%)	0	0 (0.0%)	0	0 (0.0%)	1	1 (5.3%)
- Hypokalaemia	1	1 (5.3%)	1	1 (5.3%)	0	0 (0.0%)	0	0 (0.0%)	0	0 (0.0%)	2	2 (10.5%)
SOC: Musculoskeletal and connective tissue disorders												
- Muscle contracture	0	0 (0.0%)	0	0 (0.0%)	0	0 (0.0%)	0	0 (0.0%)	1	1 (5.3%)	1	1 (5.3%)
- Rhabdomyolysis	0	0 (0.0%)	1	1 (5.3%)	0	0 (0.0%)	0	0 (0.0%)	0	0 (0.0%)	1	1 (5.3%)
- Rotator cuff syndrome	1	1 (5.3%)	0	0 (0.0%)	0	0 (0.0%)	0	0 (0.0%)	0	0 (0.0%)	1	1 (5.3%)
SOC: Nervous system disorders												
- Anosmia	0	0 (0.0%)	0	0 (0.0%)	0	0 (0.0%)	0	0 (0.0%)	1	1 (5.3%)	1	1 (5.3%)
- Convulsion	0	0 (0.0%)	1	1 (5.3%)	0	0 (0.0%)	0	0 (0.0%)	0	0 (0.0%)	1	1 (5.3%)
- Grand mal convulsion	0	0 (0.0%)	0	0 (0.0%)	1	1 (5.3%)	0	0 (0.0%)	0	0 (0.0%)	1	1 (5.3%)
- Headache	0	0 (0.0%)	1	1 (5.3%)	0	0 (0.0%)	0	0 (0.0%)	0	0 (0.0%)	1	1 (5.3%)
- Pleocytosis	1	1 (5.3%)	0	0 (0.0%)	0	0 (0.0%)	0	0 (0.0%)	0	0 (0.0%)	1	1 (5.3%)
SOC: Psychiatric disorders												
- Depression	1	1 (5.3%)	0	0 (0.0%)	0	0 (0.0%)	0	0 (0.0%)	0	0 (0.0%)	1	1 (5.3%)
- Insomnia	1	1 (5.3%)	0	0 (0.0%)	0	0 (0.0%)	0	0 (0.0%)	0	0 (0.0%)	1	1 (5.3%)
SOC: Renal and urinary disorders												
- Renal failure	0	0 (0.0%)	0	0 (0.0%)	0	0 (0.0%)	1	1 (5.3%)	0	0 (0.0%)	1	1 (5.3%)

	ongoing		recovered completely		recovered with sequelae		death		unknown		Total	
	#AE	# patients with AE	#AE	# patients with AE	#AE	# patients with AE	#AE	# patients with AE	#AE	# patients with AE	#AE	# patients with AE
SOC: Respiratory_ thoracic and mediastinal disorders												
- Hiccups	0	0 (0.0%)	1	1 (5.3%)	0	0 (0.0%)	0	0 (0.0%)	0	0 (0.0%)	1	1 (5.3%)
- Pulmonary embolism	0	0 (0.0%)	1	1 (5.3%)	0	0 (0.0%)	0	0 (0.0%)	0	0 (0.0%)	1	1 (5.3%)
SOC: Skin and subcutaneous tissue disorders												
- Drug eruption	0	0 (0.0%)	1	1 (5.3%)	0	0 (0.0%)	0	0 (0.0%)	0	0 (0.0%)	1	1 (5.3%)
- Erythema	0	0 (0.0%)	1	1 (5.3%)	0	0 (0.0%)	0	0 (0.0%)	0	0 (0.0%)	1	1 (5.3%)
- Rash	0	0 (0.0%)	1	1 (5.3%)	0	0 (0.0%)	0	0 (0.0%)	0	0 (0.0%)	1	1 (5.3%)
- Skin necrosis	0	0 (0.0%)	1	1 (5.3%)	0	0 (0.0%)	0	0 (0.0%)	0	0 (0.0%)	1	1 (5.3%)
SOC: Surgical and medical procedures												
- Endotracheal intubation	0	0 (0.0%)	1	1 (5.3%)	0	0 (0.0%)	0	0 (0.0%)	0	0 (0.0%)	1	1 (5.3%)
- Gastrostomy	0	0 (0.0%)	1	1 (5.3%)	0	0 (0.0%)	0	0 (0.0%)	0	0 (0.0%)	1	1 (5.3%)
- Tracheostomy	0	0 (0.0%)	1	1 (5.3%)	0	0 (0.0%)	0	0 (0.0%)	0	0 (0.0%)	1	1 (5.3%)
SOC: Vascular disorders												
- Deep vein thrombosis	0	0 (0.0%)	0	0 (0.0%)	0	0 (0.0%)	0	0 (0.0%)	1	1 (5.3%)	1	1 (5.3%)
- Haematoma	0	0 (0.0%)	1	1 (5.3%)	0	0 (0.0%)	0	0 (0.0%)	0	0 (0.0%)	1	1 (5.3%)
- Hypertension	1	1 (5.3%)	0	0 (0.0%)	0	0 (0.0%)	0	0 (0.0%)	0	0 (0.0%)	1	1 (5.3%)
- Thrombosis	0	0 (0.0%)	1	1 (5.3%)	0	0 (0.0%)	0	0 (0.0%)	0	0 (0.0%)	1	1 (5.3%)

Table 10.6.23: SOC / PT according to outcome - group: Dexamethasone

	ongoing		recovered completely		recovered with sequelae		death		unknown		Total	
	#AE	# patients with AE	#AE	# patients with AE	#AE	# patients with AE	#AE	# patients with AE	#AE	# patients with AE	#AE	# patients with AE
SOC: Blood and lymphatic system disorders												
- Leukocytosis	0	0 (0.0%)	1	1 (5.3%)	0	0 (0.0%)	0	0 (0.0%)	0	0 (0.0%)	1	1 (5.3%)
- Lymphadenopathy	0	0 (0.0%)	0	0 (0.0%)	0	0 (0.0%)	0	0 (0.0%)	1	1 (5.3%)	1	1 (5.3%)
SOC: Cardiac disorders												
- Arrhythmia	1	1 (5.3%)	0	0 (0.0%)	0	0 (0.0%)	0	0 (0.0%)	0	0 (0.0%)	1	1 (5.3%)
SOC: Ear and labyrinth disorders												
- Tinnitus	1	1 (5.3%)	0	0 (0.0%)	0	0 (0.0%)	0	0 (0.0%)	0	0 (0.0%)	1	1 (5.3%)
SOC: Endocrine disorders												
- Diabetes insipidus	0	0 (0.0%)	1	1 (5.3%)	0	0 (0.0%)	0	0 (0.0%)	0	0 (0.0%)	1	1 (5.3%)
SOC: Eye disorders												
- Amblyopia	1	1 (5.3%)	0	0 (0.0%)	0	0 (0.0%)	0	0 (0.0%)	0	0 (0.0%)	1	1 (5.3%)
- Diplopia	0	0 (0.0%)	1	1 (5.3%)	0	0 (0.0%)	0	0 (0.0%)	0	0 (0.0%)	1	1 (5.3%)
- Eye movement disorder	0	0 (0.0%)	1	1 (5.3%)	0	0 (0.0%)	0	0 (0.0%)	0	0 (0.0%)	1	1 (5.3%)
SOC: General disorders and administration site conditions												
- Fatigue	2	2 (10.5%)	0	0 (0.0%)	0	0 (0.0%)	0	0 (0.0%)	1	1 (5.3%)	3	3 (15.8%)
- Inflammation	1	1 (5.3%)	0	0 (0.0%)	0	0 (0.0%)	0	0 (0.0%)	0	0 (0.0%)	1	1 (5.3%)
- Pain	0	0 (0.0%)	1	1 (5.3%)	0	0 (0.0%)	0	0 (0.0%)	0	0 (0.0%)	1	1 (5.3%)
- Pyrexia	1	1 (5.3%)	2	2 (10.5%)	0	0 (0.0%)	0	0 (0.0%)	0	0 (0.0%)	3	3 (15.8%)
SOC: Infections and infestations												
- Clostridium difficile colitis	0	0 (0.0%)	1	1 (5.3%)	0	0 (0.0%)	0	0 (0.0%)	0	0 (0.0%)	1	1 (5.3%)
- Gastroenteritis rotavirus	0	0 (0.0%)	1	1 (5.3%)	0	0 (0.0%)	0	0 (0.0%)	0	0 (0.0%)	1	1 (5.3%)
- Herpes simplex meningoencephalitis	0	0 (0.0%)	0	0 (0.0%)	0	0 (0.0%)	0	0 (0.0%)	1	1 (5.3%)	1	1 (5.3%)
- Herpes zoster	0	0 (0.0%)	1	1 (5.3%)	0	0 (0.0%)	0	0 (0.0%)	0	0 (0.0%)	1	1 (5.3%)
- Pneumonia	0	0 (0.0%)	1	1 (5.3%)	0	0 (0.0%)	0	0 (0.0%)	0	0 (0.0%)	1	1 (5.3%)
SOC: Injury_ poisoning and procedural complications												
- Fall	0	0 (0.0%)	1	1 (5.3%)	0	0 (0.0%)	0	0 (0.0%)	0	0 (0.0%)	1	1 (5.3%)
- Mouth injury	0	0 (0.0%)	1	1 (5.3%)	0	0 (0.0%)	0	0 (0.0%)	0	0 (0.0%)	1	1 (5.3%)

	ongoing		recovered completely		recovered with sequelae		death		unknown		Total	
	#AE	# patients with AE	#AE	# patients with AE	#AE	# patients with AE	#AE	# patients with AE	#AE	# patients with AE	#AE	# patients with AE
SOC: Investigations												
- Blood creatinine increased	0	0 (0.0%)	1	1 (5.3%)	0	0 (0.0%)	0	0 (0.0%)	0	0 (0.0%)	1	1 (5.3%)
- Blood potassium decreased	0	0 (0.0%)	1	1 (5.3%)	0	0 (0.0%)	0	0 (0.0%)	0	0 (0.0%)	1	1 (5.3%)
- Metyrapone challenge test	0	0 (0.0%)	1	1 (5.3%)	0	0 (0.0%)	0	0 (0.0%)	0	0 (0.0%)	1	1 (5.3%)
SOC: Metabolism and nutrition disorders												
- Hyperglycaemia	0	0 (0.0%)	1	1 (5.3%)	0	0 (0.0%)	0	0 (0.0%)	0	0 (0.0%)	1	1 (5.3%)
SOC: Musculoskeletal and connective tissue disorders												
- Back pain	0	0 (0.0%)	1	1 (5.3%)	0	0 (0.0%)	0	0 (0.0%)	0	0 (0.0%)	1	1 (5.3%)
- Pain in extremity	0	0 (0.0%)	2	1 (5.3%)	0	0 (0.0%)	0	0 (0.0%)	0	0 (0.0%)	2	1 (5.3%)
SOC: Nervous system disorders												
- Aphasia	1	1 (5.3%)	0	0 (0.0%)	0	0 (0.0%)	0	0 (0.0%)	0	0 (0.0%)	1	1 (5.3%)
- Convulsion	1	1 (5.3%)	2	2 (10.5%)	0	0 (0.0%)	0	0 (0.0%)	0	0 (0.0%)	3	3 (15.8%)
- Epileptic aura	1	1 (5.3%)	0	0 (0.0%)	0	0 (0.0%)	0	0 (0.0%)	0	0 (0.0%)	1	1 (5.3%)
- Headache	0	0 (0.0%)	1	1 (5.3%)	0	0 (0.0%)	0	0 (0.0%)	1	1 (5.3%)	2	2 (10.5%)
- Memory impairment	1	1 (5.3%)	0	0 (0.0%)	0	0 (0.0%)	0	0 (0.0%)	0	0 (0.0%)	1	1 (5.3%)
SOC: Psychiatric disorders												
- Depressed mood	1	1 (5.3%)	0	0 (0.0%)	0	0 (0.0%)	0	0 (0.0%)	0	0 (0.0%)	1	1 (5.3%)
- Insomnia	1	1 (5.3%)	0	0 (0.0%)	0	0 (0.0%)	0	0 (0.0%)	0	0 (0.0%)	1	1 (5.3%)
SOC: Renal and urinary disorders												
- Renal failure	0	0 (0.0%)	1	1 (5.3%)	1	1 (5.3%)	0	0 (0.0%)	0	0 (0.0%)	2	2 (10.5%)
SOC: Respiratory_ thoracic and mediastinal disorders												
- Pneumonia aspiration	0	0 (0.0%)	0	0 (0.0%)	0	0 (0.0%)	1	1 (5.3%)	0	0 (0.0%)	1	1 (5.3%)
- Respiratory failure	0	0 (0.0%)	1	1 (5.3%)	1	1 (5.3%)	0	0 (0.0%)	0	0 (0.0%)	2	2 (10.5%)
SOC: Skin and subcutaneous tissue disorders												
- Erythema	0	0 (0.0%)	1	1 (5.3%)	0	0 (0.0%)	0	0 (0.0%)	0	0 (0.0%)	1	1 (5.3%)
- Rash	0	0 (0.0%)	1	1 (5.3%)	0	0 (0.0%)	0	0 (0.0%)	0	0 (0.0%)	1	1 (5.3%)
SOC: Social circumstances												
- Stress at work	1	1 (5.3%)	0	0 (0.0%)	0	0 (0.0%)	0	0 (0.0%)	0	0 (0.0%)	1	1 (5.3%)

	ongoing		recovered completely		recovered with sequelae		death		unknown		Total	
	#AE	# patients with AE	#AE	# patients with AE	#AE	# patients with AE	#AE	# patients with AE	#AE	# patients with AE	#AE	# patients with AE
SOC: Surgical and medical procedures												
- Endotracheal intubation	0	0 (0.0%)	0	0 (0.0%)	1	1 (5.3%)	0	0 (0.0%)	0	0 (0.0%)	1	1 (5.3%)
- Mechanical ventilation	0	0 (0.0%)	0	0 (0.0%)	1	1 (5.3%)	0	0 (0.0%)	0	0 (0.0%)	1	1 (5.3%)
SOC: Vascular disorders												
- Flushing	0	0 (0.0%)	1	1 (5.3%)	0	0 (0.0%)	0	0 (0.0%)	0	0 (0.0%)	1	1 (5.3%)
- Hypertension	0	0 (0.0%)	1	1 (5.3%)	0	0 (0.0%)	0	0 (0.0%)	0	0 (0.0%)	1	1 (5.3%)

10.7 Applied study medication

10.7.1 Full analysis set

Table 10.7.1: Acyclovir administration (FAS)

	Placebo<br>N=19	Dexamethasone<br>N=19	Total<br>N=38
Treatment days			
- N	19	19	38
- Mean +/- SD	16.1 +/-2.6	14.3 +/-4.0	15.2 +/-3.4
- Median	15.0	15.0	15.0
- p25, p75	15.0, 17.0	14.0, 15.0	14.0, 16.0
- Min, Max	13.0, 23.0	1.0, 21.0	1.0, 23.0
- Missing	0	0	0
Total amount (mg)			
- N	19	19	38
- Mean +/- SD	35605.3 +/-7384.4	31597.4 +/-10349.2	33601.3 +/-9097.1
- Median	33000.0	33000.0	33000.0
- p25, p75	31500.0, 39000.0	29250.0, 36000.0	30750.0, 37500.0
- Min, Max	21500.0, 50250.0	2250.0, 46500.0	2250.0, 50250.0
- Missing	0	0	0

Table 10.7.2: Dexamethasone/Placebo administration (FAS)

	Placebo<br>N=19	Dexamethasone<br>N=19	Total<br>N=38
Treatment days			
- 3	0 (0.0%)	1 (5.3%)	1 (2.6%)
- 4	19 (100.0%)	18 (94.7%)	37 (97.4%)

10.7.2 Per protocol set

Table 10.7.3: Acyclovir administration (PP set)

	Placebo N=9	Dexamethasone N=7	Total N=16
Treatment days			
- N	9	7	16
- Mean +/- SD	15.8 +/-1.9	14.9 +/-0.7	15.4 +/-1.5
- Median	15.0	15.0	15.0
- p25, p75	15.0, 16.0	14.0, 15.0	14.5, 16.0
- Min, Max	14.0, 20.0	14.0, 16.0	14.0, 20.0
- Missing	0	0	0
Total amount (mg)			
- N	9	7	16
- Mean +/- SD	34750.0 +/-3842.6	34628.6 +/-4704.1	34696.9 +/-4090.3
- Median	33750.0	33750.0	33750.0
- p25, p75	33000.0, 35250.0	31500.0, 36000.0	32250.0, 35625.0
- Min, Max	30750.0, 43500.0	30000.0, 44400.0	30000.0, 44400.0
- Missing	0	0	0

Table 10.7.4: Dexamethasone/Placebo administration (PP set)

	Placebo N=9	Dexamethasone N=7	Total N=16
Treatment days			
- 4	9 (100.0%)	7 (100.0%)	16 (100.0%)

10.8 Study termination

Table 10.8.1: Study termination (FAS)

	Placebo N=19	Dexamethasone N=19	Total N=38
Study termination			
- Completed entire course	14 (73.7%)	12 (63.2%)	26 (68.4%)
- Premature termination	5 (26.3%)	7 (36.8%)	12 (31.6%)
Reason for premature termination			
- Lost to Follow-Up	0 (0.0%)	4 (57.1%)	4 (33.3%)
- Withdrawal of consent	2 (40.0%)	1 (14.3%)	3 (25.0%)
- Serious Adverse Event	0 (0.0%)	1 (14.3%)	1 (8.3%)
- Death	2 (40.0%)	1 (14.3%)	3 (25.0%)
- Other reason	1 (20.0%)	0 (0.0%)	1 (8.3%)
- Missing	14	12	26
Days in study			
- N	19	19	38
- Mean +/- SD	333.5 +/-80.6	294.0 +/-181.2	313.8 +/-139.8
- Median	369.0	366.0	368.0
- p25, p75	281.0, 378.0	157.0, 390.0	232.0, 385.0
- Min, Max	166.0, 438.0	4.0, 665.0	4.0, 665.0
- Missing	0	0	0

10.9 Other analyses

10.9.1 Seizures

10.9.1.1 FAS

Table 10.9.1: Seizures since last visit (FAS)

	Placebo N=19	Dexamethasone N=19	Total N=38	p-value
Discharge/Day 30 Seizures				
- no	13 (72.2%)	11 (64.7%)	24 (68.6%)	0.632 *1
- yes	5 (27.8%)	6 (35.3%)	11 (31.4%)	
- Missing	1	2	3	
6 Months Seizures				
- no	15 (88.2%)	12 (92.3%)	27 (90.0%)	0.713 *1
- yes	2 (11.8%)	1 (7.7%)	3 (10.0%)	
- Missing	2	6	8	
12 Months Seizures				
- no	14 (100.0%)	11 (91.7%)	25 (96.2%)	0.271 *1
- yes	0 (0.0%)	1 (8.3%)	1 (3.8%)	
- Missing	5	7	12	

*1 = chi^2-test, two-sided

10.9.1.2 PP set

Table 10.9.2: Seizures since last visit (PP set)

	Placebo N=9	Dexamethasone N=7	Total N=16	p-value
Discharge/Day 30 Seizures				
- no	6 (75.0%)	3 (60.0%)	9 (69.2%)	0.569 *1
- yes	2 (25.0%)	2 (40.0%)	4 (30.8%)	
- Missing	1	2	3	
6 Months Seizures				
- no	9 (100.0%)	6 (100.0%)	15 (100.0%)	
- Missing	0	1	1	
12 Months Seizures				
- no	7 (100.0%)	6 (100.0%)	13 (100.0%)	
- Missing	2	1	3	

*1 = chi^2-test, two-sided

11 References

GACHE Clinical Trial Protocol. German Trial of Acyclovir and Corticosteroids in Herpes-Simplex-Virus-Encephalitis. Clinical Trial Protocol; Final Version 10-02-2009; EudraCT-Nr: 2005-003201-81.

GACHE Statistical Analysis Plan. German Trial of Acyclovir and Corticosteroids in Herpes-Simplex-Virus-Encephalitis. Statistical Analysis Plan; Version 08-04-2014; EudraCT-Nr: 2005-003201-81.

Müller HH, Schäfer H (2001). Adaptive group sequential designs for clinical trials: Combining the advantages of adaptive and of classical group sequential approaches. Biometrics 57, 886-891.

O'Brien PC; Fleming TR. (1979). A multiple testing procedure for clinical trials. Biometrics 35, 549–556.

Wilson, L. Hareendran, A. Structured Interview for the Modified Rankin Scale – Questionnaire and Guidelines. 2002.

12 Listings

12.1 Excluded patients

Patient Identification	Treatment group	Full analysis set (FAS)	Exclusion FAS	Per protocol set (PP)	Exclusion PP	Safety set	Exclusion Safety set
02-005	Dexamethasone	yes		no	No month 6 visit done	yes	
03-005	Dexamethasone	yes		no	Month 6 visit not within +-14 days	yes	
03-007	Placebo	yes		no	Inclusion/Exclusion criteria violated; Month 6 visit not within +-14 days; Excluded from PP set due to contradictory documentation of pre-encephalitis mRS	yes	
07-047	Dexamethasone	yes		no	Excluded from PP set due to contradictory documentation of exclusion criterion (Barthel Index < 95) and pre-encephalitis Barthel index	yes	
07-102	Dexamethasone	yes		no	No month 6 visit done	yes	
08-004	Dexamethasone	yes		no	No month 6 visit done	yes	
08-009	Placebo	yes		no	No month 6 visit done	yes	
11-001	Dexamethasone	yes		no	Less than 4 days dexamethasone/placebo treatment; No month 6 visit done	yes	
12-009	Dexamethasone	no	No study medication	no	Inclusion/Exclusion criteria violated; Less than 4 days dexamethasone/placebo treatment ; No month 6 visit done	no	No study medication
12-092	Dexamethasone	yes		no	Month 6 visit not within +-14 days	yes	
12-093	Dexamethasone	yes		no	No month 6 visit done	yes	
12-094	Placebo	yes		no	Patient not entered in randomizer; No month 6 visit done	yes	
13-001	Placebo	yes		no	Excluded from PP set due to contradictory documentation of pre-encephalitis mRS	yes	
16-019	Placebo	yes		no	Inclusion/Exclusion criteria violated; Month 6 visit not within +-14 days; Excluded from PP set due to contradictory documentation of exclusion criterion (Barthel Index < 95) and pre-encephalitis Barthel index	yes	
16-020	Dexamethasone	yes		no	Month 6 visit not within +-14 days	yes	

Patient Identification	Treatment group	Full analysis set (FAS)	Exclusion FAS	Per protocol set (PP)	Exclusion PP	Safety set	Exclusion Safety set
18-001	Placebo	yes		no	Month 6 visit not within +-14 days; Excluded from PP set due to contradictory documentation of exclusion criterion (Barthel Index < 95) and pre-encephalitis Barthel index	yes	
19-007	Placebo	yes		no	Excluded from PP set due to contradictory documentation of exclusion criterion (Barthel Index < 95) and pre-encephalitis Barthel index	yes	
21-005	Dexamethasone	yes		no	Month 6 visit not within +-14 days	yes	
21-011	Dexamethasone	yes		no	Month 6 visit not within +-14 days	yes	
21-012	Placebo	yes		no	Month 6 visit not within +-14 days	yes	
21-013	Placebo	yes		no	Month 6 visit not within +-14 days	yes	
22-001	Placebo	yes		no	Month 6 visit not within +-14 days; Excluded from PP set due to contradictory documentation of exclusion criterion (Barthel Index < 95) and pre-encephalitis Barthel index	yes	
26-051	Dexamethasone	yes		no	Month 6 visit not within +-14 days	yes	

12.2 Adverse events/serious adverse event

Patiet/ID	Sign/symptom	Onset date	Stop date	SAE	Severity	Causality to study drug	No action	Dosage change	Study drug interrupted	Study drug discontinued	New therapy added	Conc. therapy changed	Hospital-ization	Outcome
02-005/D	seizures	nk/052008	/ /	no	severe	none					yes			ongoing
02-005/D	Fever of unknown origin	02/06/2008	08/09/2008	no	moderate	none					yes			recovered completely
03-005/D	back pain	03/07/2008	03/07/2008	no	moderate	none					yes			recovered completely
03-005/D	abnormal fatigue	11/02/2009	17/02/2009	yes	moderate	none	yes							unknown
03-005/D	Leucocytosis 17,4	27/06/2008	02/07/2008	no	moderate	possible	yes							recovered completely
03-005/D	pain in left leg	03/07/2008	07/07/2008	no	moderate	none					yes			recovered completely
03-005/D	pain in the left leg	11/02/2009	17/02/2009	no	mild	none	yes							recovered completely
03-007/P	pulmonal infection	15/01/2012	19/01/2012	no	moderate	none					yes			recovered completely
03-007/P	pulmonal infection	30/04/2012	08/05/2012	yes	moderate	none					yes			recovered completely
03-007/P	obstipation	18/01/2012	20/01/2012	no	mild	none					yes			recovered completely
03-007/P	diarrhoea	27/01/2012	28/01/2012	no	mild	none					yes			recovered completely
03-007/P	hamatoma left eye	20/08/2012	10/01/2013	no	mild	none	yes							recovered completely
03-007/P	mild headeache	06/02/2012	06/02/2012	no	mild	none	yes							recovered completely
03-007/P	swelling left hand	23/01/2012	24/01/2012	no	mild	none					yes			recovered completely
03-007/P	burning pain	01/02/2012	01/02/2012	no	mild	none	yes							recovered completely
03-007/P	fever	03/02/2012	04/02/2012	no	mild	unlikely						yes		recovered completely
03-007/P	fever	17/01/2012	17/01/2012	no	mild	possible	yes							recovered completely
03-007/P	fever	23/01/2012	24/01/2012	no	mild	unlikely	yes							recovered completely
03-007/P	fever	27/01/2012	30/01/2012	no	moderate	unlikely					yes			recovered completely
03-007/P	reduced general condition	12/01/2012	/ /	no	severe	none	yes							ongoing
07-017/P	nausea	08/08/2008	08/08/2008	no	mild	not assessable					yes			recovered completely

Patiet/ID	Sign/symptom	Onset date	Stop date	SAE	Severity	Causality to study drug	No action	Dosage change	Study drug interrupted	Study drug discontinued	New therapy added	Conc. therapy changed	Hospital-ization	Outcome
07-017/P	vomitting	08/08/2008	08/08/2008	no	mild	not assessable					yes			recovered completely
07-102/D	Progression of symptoms and inflamation to contralateral side	05/11/2009	nk/nknk	yes	severe	not assessable				yes				unknown
08-004/D	Diabetes Insipidus	30/12/2009	10/01/2010	no	moderate	unlikely					yes			recovered completely
08-004/D	Fever (Central)	03/01/2010	12/01/2010	no	mild	none					yes			ongoing
08-009/P	bakteriemia	18/06/2010	28/06/2010	no	mild	unlikely						yes		recovered completely
08-009/P	positive bloodcultures	18/06/2010	28/06/2010	no	mild	unlikely						yes		recovered completely
08-009/P	Exanthema	22/06/2010	29/06/2010	no	mild	possible					yes			recovered completely
08-009/P	Rhabdomyolysis	21/06/2010	23/06/2010	no	mild	unlikely						yes		recovered completely
11-001/D	Flush	12/01/2009	13/01/2009	no	mild	possible	yes							recovered completely
12-063/D	Doppelbilder	11/10/2010	nk/032011	no	mild	none	yes							recovered completely
12-063/D	Pneumonie	01/04/2010	14/04/2010	no	severe	none	yes							recovered completely
12-063/D	respirat. Insuff.	01/04/2010	19/05/2010	no	severe	none	yes							recovered completely
12-064/P	lost of smell	nk/102010	nk/nknk	no	mild	none	yes							unknown
12-092/D	arrhytmia requiring pacemaker	18/03/2011	/ /	no	mild	none					yes			ongoing
12-094/P	Abscess	17/11/2011	20/11/2011	yes	severe	none	yes							death
12-094/P	Cholangitis	17/11/2011	20/11/2011	yes	severe	none	yes							death
12-094/P	renal failure	19/11/2011	20/11/2011	yes	severe	none	yes							death
12-094/P	sepsis	18/11/2011	20/11/2011	yes	severe	none	yes							death
13-001/P	Fall	10/11/2007	10/11/2007	no	mild	none	yes							recovered completely
13-001/P	hypertension	nk/122007	/ /	no	moderate	unlikely					yes			ongoing
13-001/P	CSF pleocytosis	14/11/2007	/ /	no	mild	none					yes			ongoing
13-001/P	shoulder impingement	22/11/2007	/ /	no	moderate	unlikely	yes							ongoing
13-001/P	vision problems	15/04/2008	/ /	no	mild	unlikely	yes							ongoing
13-001/P	weight loss	21/11/2007	28/11/2008	no	mild	none	yes							recovered completely
13-001/P	loss of appetite	21/11/2007	/ /	no	mild	none	yes							ongoing

Patiet/ID	Sign/symptom	Onset date	Stop date	SAE	Severity	Causality to study drug	No action	Dosage change	Study drug interrupted	Study drug discontinued	New therapy added	Conc. therapy changed	Hospital-ization	Outcome
13-002/P	Tachyarrhythmia absoluta	10/02/2011	17/02/2011	no	moderate	none					yes			recovered completely
13-002/P	drastic worsening of patents condition	nk/112011	24/11/2011	yes	severe	none	yes							death
13-002/P	drug eczema	18/02/2011	21/02/2011	no	mild	none						yes		recovered completely
13-002/P	Hydrocele testis	22/02/2011	/ /	no	mild	none	yes							unknown
13-002/P	recurrent pneumonia	11/02/2011	04/03/2011	no	moderate	none					yes			recovered completely
13-002/P	pulmonary embolism	16/02/2011	16/02/2011	no	moderate	none						yes		recovered completely
13-002/P	urinary tract Infection	31/03/2011	09/04/2011	no	mild	none					yes			recovered completely
13-002/P	deep vene thrombosis	15/02/2011	/ /	no	moderate	none						yes		unknown
13-002/P	slight contractures head/neck	18/08/2011	/ /	no	moderate	none	yes							unknown
13-003/D	Repeated epigastric epileptic auras	09/09/2011	/ /	no	mild	none					yes			ongoing
13-003/D	Fatigue	09/09/2011	/ /	no	mild	none					yes			ongoing
13-003/D	professional stress	09/09/2011	/ /	no	mild	none					yes			ongoing
13-004/D	Clostridium difficile enterocolitis	20/07/2011	02/08/2011	no	moderate	unlikely					yes			recovered completely
13-004/D	Headeach	01/12/2011	31/05/2012	no	mild	none	yes							recovered completely
16-009/D	Depressive mood disorder	11/11/2007	/ /	no	mild	possible					yes			ongoing
16-009/D	Hyperglycemia with requirement of subcutaneous insulin	03/11/2007	07/11/2007	no	mild	probable					yes			recovered completely
16-009/D	Pain, unspezified	14/11/2007	14/11/2007	no	mild	none					yes			recovered completely
16-009/D	Aspiration Pneumonia	23/11/2007	11/12/2007	yes	severe	none		yes						death
16-009/D	Inflammation left forearm	14/11/2007	/ /	no	mild	none					yes			ongoing
16-019/P	New HSV-lessions	10/01/2012	18/01/2012	yes	mild	none							yes	recovered completely
16-019/P	Hypokaliemia	09/05/2011	/ /	no	mild	none					yes			ongoing
16-019/P	Sleeping disorder	09/05/2011	/ /	no	mild	none					yes			ongoing
16-019/P	Edema right arm	16/05/2011	16/05/2011	no	mild	none	yes							recovered completely
16-019/P	Chambylobacter infection	12/05/2011	26/05/2011	no	mild	none					yes			recovered completely

Patiet/ID	Sign/symptom	Onset date	Stop date	SAE	Severity	Causality to study drug	No action	Dosage change	Study drug interrupted	Study drug discontinued	New therapy added	Conc. therapy changed	Hospital-ization	Outcome
16-020/D	Fall	06/06/2011	06/06/2011	no	moderate	none	yes							recovered completely
16-020/D	Planned Metopiron testing	23/03/2012	25/03/2012	yes	mild	none	yes							recovered completely
16-020/D	Extirpation Hypophysenadenoma	23/01/2012	30/01/2012	yes	moderate	none	yes							recovered completely
16-020/D	Lac eration upper lip	06/06/2011	13/06/2011	no	moderate	none	yes							recovered completely
16-021/P	Suspected brain abszess	12/01/2012	17/01/2012	yes	moderate	none	yes							recovered completely
19-010/D	seizure	24/06/2009	24/06/2009	no	moderate	unlikely					yes			recovered completely
19-010/D	renal failure	16/07/2009	06/08/2009	yes	severe	none							yes	recovered with sequelae
19-010/D	respiratory insufficency	25/06/2009	05/08/2009	yes	moderate	unlikely	yes							recovered with sequelae
19-010/D	bulbus deviation	01/07/2009	21/12/2009	no	mild	none	yes							recovered completely
19-010/D	ventilated mechanically	25/06/2009	05/08/2009	yes	moderate	unlikely	yes							recovered with sequelae
19-010/D	need to be intubated	25/06/2009	05/08/2009	yes	moderate	unlikely	yes							recovered with sequelae
20-004/P	depression	nk/112008	/ /	no	moderate	none					yes			ongoing
20-004/P	herpes simplex	nk/nk2009	nk/032009	no	moderate	none					yes			recovered completely
20-004/P	Hypopotassemia	29/07/2008	01/08/2008	no	moderate	none					yes			recovered completely
20-004/P	fever	29/07/2008	nk/082008	no	moderate	none					yes			recovered completely
21-004/P	Elevated bodytemperature (<39,5 C)	17/07/2008	19/07/2008	no	mild	none					yes			recovered completely
21-004/P	1. Grand mal/epilepsy	18/07/2008	18/07/2008	no	moderate	unlikely					yes			recovered with sequelae
21-004/P	Singultus	24/07/2008	04/08/2008	no	mild	none					yes			recovered completely
21-004/P	High fever (40 C)	03/08/2008	07/08/2008	no	moderate	none					yes			recovered completely
21-004/P	Sepsis	06/08/2008	10/08/2008	yes	severe	none					yes		yes	recovered completely
21-004/P	Thrombosis of V. jugularis int. lt.	06/08/2008	10/08/2008	yes	severe	none					yes		yes	recovered completely

Patiet/ID	Sign/symptom	Onset date	Stop date	SAE	Severity	Causality to study drug	No action	Dosage change	Study drug interrupted	Study drug discontinued	New therapy added	Conc. therapy changed	Hospital-ization	Outcome
21-005/D	Erythema (periumbilical)	24/04/2009	nk/052009	no	mild	unlikely					yes			recovered completely
21-005/D	Renal insufficiency	20/04/2009	nk/052009	no	mild	unlikely						yes		recovered completely
21-008/P	ongoing seizures (non-konvuls state)	24/09/2009	28/09/2009	no	moderate	none					yes			recovered completely
21-008/P	skin necrosis left hand	28/09/2009	03/11/2009	no	moderate	none					yes			recovered completely
21-008/P	Tracheotomy	07/10/2009	//122009	no	moderate	none					yes			recovered completely
21-008/P	PEG	22/10/2009	25/02/2010	no	moderate	none					yes			recovered completely
21-008/P	paravasate of phenytoin	28/09/2009	03/11/2009	no	moderate	none					yes			recovered completely
21-008/P	Intubation	24/09/2009	07/10/2009	no	severe	none					yes			recovered completely
21-011/D	creatinine increased	19/02/2010	21/02/2010	no	mild	unlikely		yes						recovered completely
21-011/D	low potassium	20/02/2010	23/02/2010	no	mild	none					yes			recovered completely
21-011/D	Diarrhea, Noro-Virusinfection	17/02/2010	22/02/2010	no	moderate	none	yes							recovered completely
21-013/P	rise of creatinine	07/03/2012	09/03/2012	no	mild	none						yes		recovered completely
26-010/P	GPT increased >330 U/L	17/03/2008	/ /	no	moderate	none						yes		ongoing
26-010/P	AP increased	20/03/2008	/ /	no	mild	none	yes							ongoing
26-010/P	bronchitis	13/03/2008	27/03/2008	no	moderate	none						yes		recovered completely
26-010/P	CRP increased >70 mg/l	14/03/2008	25/03/2008	no	mild	none	yes							recovered completely
26-010/P	fall	22/03/2008	22/03/2008	no	mild	none	yes							recovered completely
26-010/P	Lipase increased	20/03/2008	/ /	no	mild	none	yes							ongoing
26-010/P	recurrent fever	13/03/2008	27/03/2008	no	moderate	none						yes		recovered completely
26-010/P	skin rush	19/03/2008	22/03/2008	no	mild	none						yes		recovered completely
26-010/P	vomiting	16/03/2008	16/03/2008	no	mild	none					yes			recovered completely
26-013/D	sleep disorder	nk/102008	/ /	no	mild	none	yes							ongoing
26-013/D	Tinitus	12/03/2009	/ /	no	mild	none	yes							ongoing

Patiet/ID	Sign/symptom	Onset date	Stop date	SAE	Severity	Causality to study drug	No action	Dosage change	Study drug interrupted	Study drug discontinued	New therapy added	Conc. therapy changed	Hospital-ization	Outcome
26-030/D	motoric aphasia intermittend	13/10/2008	/ /	no	mild	none	yes							ongoing
26-030/D	Seizure (nonconvulsive)	10/10/2008	10/10/2008	no	moderate	none					yes			recovered completely
26-030/D	Fatigue intermittend	13/10/2008	/ /	no	mild	none	yes							ongoing
26-030/D	Herpes Zosta	nk/122008	nk/032009	no	moderate	none					yes			recovered completely
26-030/D	Swelling of the right supraclavicular lymp nodes	12/10/2009	nk/nknk	no	mild	none	yes							unknown
26-051/D	ambyoplia	//122011	/ /	no	mild	none	yes							ongoing
26-051/D	headache	//032011	nk/nknk	no	mild	none	yes							unknown
26-051/D	arterial hypertonia	//032011	nk/nknk	no	mild	none	yes							recovered completely
26-051/D	memory disorders	//062011	nk/nknk	no	moderate	none	yes							ongoing
26-051/D	Fever 39 grade C	16/02/2011	16/02/2011	no	mild	none	yes							recovered completely

13 Appendix

13.1 Original SAS® output for primary endpoint

Table of binary_mRS_LOCF_original by group

binary_mRS_LOCF_original(mRS) / Frequency / Percent / Col Pct	group(Group) Placebo	Dexamethasone	Total
<=2	12<br>31.58<br>63.16	12<br>31.58<br>63.16	24<br>63.16
>2	7<br>18.42<br>36.84	7<br>18.42<br>36.84	14<br>36.84
Total	19<br>50.00	19<br>50.00	38<br>100.00

Statistics for Table of binary_mRS_LOCF by group

Statistic	DF	Value	Prob
Chi-Square	1	0.0000	1.0000
Likelihood Ratio Chi-Square	1	0.0000	1.0000
Continuity Adj. Chi-Square	1	0.0000	1.0000
Mantel-Haenszel Chi-Square	1	0.0000	1.0000
Phi Coefficient		0.0000	
Contingency Coefficient		0.0000	
Cramer's V		0.0000	

Fisher's Exact Test	
Cell (1,1) Frequency (F)	12
Left-sided Pr <= F	0.6313
Right-sided Pr >= F	0.6313
Table Probability (P)	0.2626
Two-sided Pr <= P	1.0000

Sample Size = 38

Unresolved Data Issues

Derivation of scores

In cases where total scores and the respective single items were documented in the CRF, total scores will also be derived from single items of a questionnaire/index. The calculated score will be used in case of contradictions between calculated and documented total scores. If at least one single item is missing, the total score is set to missing.

Other unresolved issues

Patient	Unresolved Issue	Handling
**Pre-encephalitis Barthel Index**		
07-047	Pre-encephalitis Barthel Index = 10<br>Exclusion criterion ‘Barthel Index < 95’ documented as ‘no’.	Included in FAS.<br>Excluded from PP set due to contradictory documentation of exclusion criterion (Barthel Index < 95) and pre-encephalitis Barthel index.
16-019	Documented pre-encephalitis Barthel Index = 100.<br>From single items calculated pre-encephalitis Barthel Index = 90.<br>Exclusion criterion ‘Barthel Index < 95’ documented as ‘no’.	Included in FAS.<br>Score will be calculated from single items.<br>Excluded from PP set due to contradictory documentation of exclusion criterion (Barthel Index < 95) and pre-encephalitis Barthel index.
18-001	Pre-encephalitis Barthel Index = 25.<br>Exclusion criterion ‘Barthel Index < 95’ documented as ‘no’.	Included in FAS.<br>Excluded from PP set due to contradictory documentation of exclusion criterion (Barthel Index < 95) and pre-encephalitis Barthel index.
19-007	Documented pre-encephalitis Barthel Index = 0.<br>From single items calculated pre-encephalitis Barthel Index = 20.<br>Exclusion criterion ‘Barthel Index < 95’ documented as ‘no’.	Included in FAS.<br>Score will be calculated from single items.<br>Excluded from PP set due to contradictory documentation of exclusion criterion (Barthel Index < 95) and pre-encephalitis Barthel index.

22-001	Pre-encephalitis Barthel Index = 90.<br>Exclusion criterion ‘Barthel Index < 95’ documented as ‘no’.	Included in FAS.<br>Excluded from PP set due to contradictory documentation of exclusion criterion (Barthel Index < 95) and pre-encephalitis Barthel index.
**Pre-encephalitis mRS**		
03-007	Documented pre-encephalitis mRS = 2 at baseline.<br>Pre-encephalitis mRS > 2 according to mRS questionaire documentation at visit 4.<br>Exclusion criterion ‘mRS > 2’ documented as ‘no’.	Included in FAS.<br>Excluded from PP set due to contradictory documentation of pre-encephalitis mRS.
13-001	Documented pre-encephalitis mRS = 0 at baseline.<br>Pre-encephalitis mRS > 2 according to mRS questionaire documentation at visit 4.<br>Exclusion criterion ‘mRS > 2’ documented as ‘no’.	Included in FAS.<br>Excluded from PP set due to contradictory documentation of pre-encephalitis mRS.
**mRS visit 4**		
03-005	According to documentation, patient was not looking after family at home before encephalitis but patient’s ability to look after family at home was reduced after encephalitis.<br>Depending on handling of this item mRS is either 2 or 1.	mRS is set to 1.<br>Scoring will be done according to manual and question will be skipped since patient has not been looking after family before encephalitis.
12-033	According to documentation, patient was not working or seeking work before encephalitis but has a reduced level of work after encephalitis.<br>Depending on handling of this item mRS is either 2 or 1.	mRS is set to 1.<br>Scoring will be done according to manual and question will be skipped since patient has not been working or seeking work before encephalitis.
**mRS visit 5**		
12-033	According to documentation, patient was not working or seeking work before encephalitis but has a reduced level of work after encephalitis.<br>Depending on handling of this item mRS is either 2 or 1.	mRS is set to 1.<br>Scoring will be done according to manual and question will be skipped since patient has not been working or seeking work before encephalitis.

Others

08-004	Baseline: GCS<br>Implausible item 'Verbal' (documented response = 0; range: 1 to 5).<br>Documented total score = 9 (Eye opening = 3, motor response = 6).	Total GCS and GCS verbal score set to missing due to implausible item 'Verbal'.
07-017	Day 0: GCS<br>Implausible item 'Verbal' (documented response = 6; range: 1 to 5).<br>Implausible documented total score = 16 (range: 3 to 15).	Total GCS and GCS verbal score set to missing due to implausible item 'Verbal'.
19-007	Visit 4: NIH<br>Items 'Limb ataxia' and 'Sensory' are missing (CRF documentation: 'not scored').<br>Documented total score (= 19) ignores missing items.	Unknown total score due to unknown items.<br>Therefore the NIH total score will be set to missing.
19-010	Month 6: Barthel Index<br>Implausible item 'Stairs' (documented response Stairs = 15; allowed: 0,5,10).<br>Documented Barthel Index = 95.	Item 'Stairs' and Barthel Index set to missing due to implausible item 'Stairs'.

I hereby confirm that the above listed data issues cannot be resolved before database closure and will be handled as outlined in this document.

Prof. Dr. Meinhard Kieser<br>Responsible Biometrician	02.03.2015<br>Date and signature of the responsible Biometrician
Anja Sander<br>Representative of the responsible Biometrician	02.03.2015<br>Date and signature of the representative of the responsible Biometrician
Prof. Dr. med Uta Meyding-Lamadé<br>Coordinating Investigator	5.3.2015<br>Date and signature of the Coordinating Investigator
Prof. Dr. med. Werner Hacke<br>Sponsor	17.3 2015<br>Date and signature of the Sponsor
